# Trends and projections of global testicular cancer burden from 1990 to 2035

**DOI:** 10.3389/fonc.2026.1634710

**Published:** 2026-03-04

**Authors:** Changkun Mao, Chengpin Tao, Chao Yang, Jian Shen, Guangyuan Li

**Affiliations:** 1 Department of Urology, Lu’an Municipal People’s Hospital, Lu’an Hospital of Anhui Medical University, Lu’an, China; 2 Department of Urology, Anhui Provincial Children’s Hospital, Hefei, China; 3 Department of Urology, Children’s Hospital of Fudan University, Shanghai, China

**Keywords:** age-period-cohort, Bayesian, epidemiology, Global Burden of Disease, testicular cancer, trend analysis

## Abstract

**Background:**

Testicular cancer (TC) is the most common malignancy in young men, with incidence increasing globally, especially in high-income countries. Although survival has improved due to advances in diagnosis and treatment, disparities in TC burden remain. This study analyzes global, regional, and national trends in TC incidence, mortality, and disability-adjusted life years (DALYs) from 1990 to 2021, and projects future trends to 2035.

**Methods:**

Data were obtained from the Global Burden of Disease (GBD) 2021 database. Incidence, mortality, and DALY rates per 100,000 population were calculated with 95% uncertainty intervals (UIs). Trend analysis used Joinpoint regression and estimated annual percentage change (EAPC). Decomposition analysis identified drivers of burden changes. A Bayesian age-period-cohort (BAPC) model projected future burden.

**Results:**

In 2021, there were 91,507 TC cases, 11,388 deaths, and 560,921 DALYs globally. From 1990 to 2021, cases rose by 136%, deaths by 49%, and DALYs by 44%. Incidence increased from 1.45 to 2.31 per 100,000. The middle socio-demographic index (SDI) region showed the highest EAPCs for incidence (4.34%), mortality (1.07%), and DALYs (0.92%). The Caribbean had the fastest-growing incidence (EAPC = 5.71%). Nationally, the U.S. had the most cases (11,845), Monaco the highest incidence (32.89/100,000), and Qatar the steepest rise (EAPC = 10.25%). By 2035, incidence is projected to rise further, while mortality and DALY rates may decline.

**Conclusion:**

The global burden of TC has increased markedly since 1990, especially in middle-SDI regions and the Caribbean. Although some areas have seen improvements, rising incidence highlights the need for targeted prevention and optimized care strategies.

## 1 Introduction

Testicular cancer (TC), though relatively uncommon, represents the most frequent malignancy among adolescent and young adult (AYA) males (15–40 years) globally (1). Although its prognosis is generally favorable, survivors face significant challenges, including an increased risk of infertility, sexual dysfunction, and other side effects of treatment (2). While comprising less than 1% of all male cancers, its incidence has been steadily rising, particularly in high-income countries, contrasting with lower rates in most Asian and African regions (3). Advances in diagnosis and platinum-based chemotherapy have significantly improved survival rates; however, the overall disease burden—encompassing incidence, mortality, and disability-adjusted life years (DALYs)—continues to escalate, posing considerable challenges for healthcare systems and policy development (3, 4).

Previous epidemiological research over recent decades has consistently documented an increasing TC incidence and marked spatiotemporal disparities across nations (5). Nonetheless, a comprehensive, up-to-date assessment of the global, regional, and national TC burden, especially one that dynamically analyzes trends in incidence, mortality, and DALYs while also forecasting future trajectories using the latest available data, remains a critical need. Existing studies often lack the granularity or the predictive component necessary to fully inform targeted public health interventions. This study addresses this gap by leveraging the Global Burden of Disease (GBD) 2021 database to systematically evaluate TC burden across 204 countries and territories from 1990 to 2021. Furthermore, employing a Bayesian Age-Period-Cohort (BAPC) model, we project these trends to 2035. Our objective is to furnish robust, evidence-based insights to guide the formulation and prioritization of global TC prevention and control strategies, thereby mitigating its growing public health impact, particularly in identified high-risk or rapidly changing regions.

## 2 Methods

### 2.1 Data source and case definition

Data were extracted from the Global Burden of Disease (GBD) Study 2021, coordinated by the Institute for Health Metrics and Evaluation (IHME) (6). GBD 2021 provides systematic estimates of health loss for 371 diseases and injuries and 88 risk factors across 204 countries/territories and 21 GBD regions (6). Detailed GBD 2021 methodology and results are publicly available (https://vizhub.healthdata.org/gbd-results/).

Testicular cancer (TC) cases were identified using International Classification of Diseases (ICD) codes: ICD-10 (C62-C62.9, D29.2-D29.8, D40.1-D40.8) and ICD-9 (186-186.9, 222.0, 222.3, 236.4) (6). We retrieved annual TC incidence, mortality, and disability-adjusted life years (DALYs) for individuals aged 15 years and older from 1990 to 2021, as GBD data for TC in younger age groups were unavailable. Age subgroups (e.g., 15-19, 20-24, …, ≥75 years) were analyzed.

### 2.2 Socio-demographic index

The SDI, a composite measure ranging from 0 (least developed) to 1 (most developed), was used to stratify countries and regions. It is based on per capita income, average years of education, and total fertility rate (7). GBD 2021 categorizes locations into five SDI quintiles: low, low-middle, middle, high-middle, and high (7). Further details on SDI calculation are provided in the **Supplementary Materials**.

### 2.3 Statistical analysis

We calculated TC incidence, mortality, and DALY rates per 100,000 population with 95% uncertainty intervals (UIs). To quantify temporal trends from 1990 to 2021, the estimated annual percentage change (EAPC) with its 95% confidence interval (CI) was calculated using a log-transformed linear regression model (ln(rate) = α + β × year + ϵ). An EAPC > 0 indicates an increasing trend, EAPC < 0 a decreasing trend, and EAPC ≈ 0 a stable trend. Joinpoint regression analysis (Joinpoint Regression Program, Version 4.9.1.0; National Cancer Institute) was used to identify significant changes in trends over time, calculating the annual percentage change (APC) for each segment and the average annual percentage change (AAPC) over the entire period.

Decomposition analysis, using the Das Gupta method, was performed to attribute changes in the absolute number of TC incident cases, deaths, and DALYs between 1990 and 2021 to three factors: population growth, population aging (changes in age structure), and epidemiological changes (changes in age-specific rates). Details of this method are available in prior GBD publications (6).

Future trends in TC incidence, deaths, and DALYs, along with their age-standardized rates, were projected up to 2035 using a Bayesian age-period-cohort (BAPC) model. This model, implemented using the BAPC R package, incorporates age, period, and cohort effects and utilizes Integrated Nested Laplace Approximation (INLA) for efficient computation, leveraging GBD 2021 population projections. All statistical analyses were performed using R software (version 4.3.3). Results were visualized using tables and maps. A two-sided p-value < 0.05 was considered statistically significant.

## 3 Results

### 3.1 Global burden and temporal trends of testicular cancer

**Figure 1:**
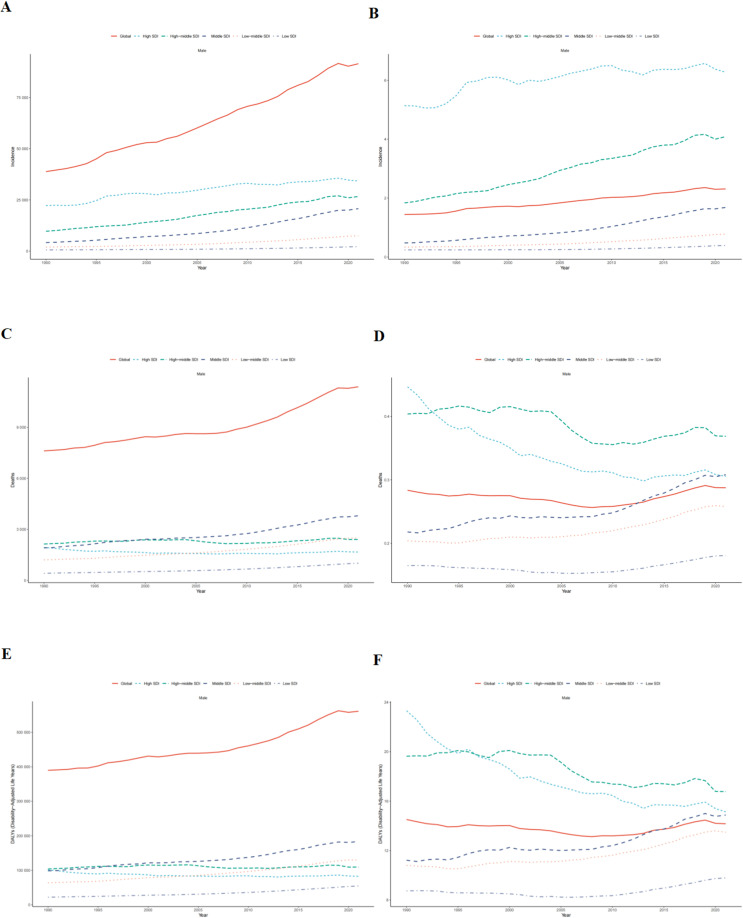
Trends in testicular cancer from 1990 to 2021 at the global level and across five SDI regions. **(A)** Number of incidence cases. **(B)** Incidence rate. **(C)** Number of death cases. **(D)** Mortality rate. **(E)** Number of DALYs. **(F)** DALYs rate.

**Figure 2:**
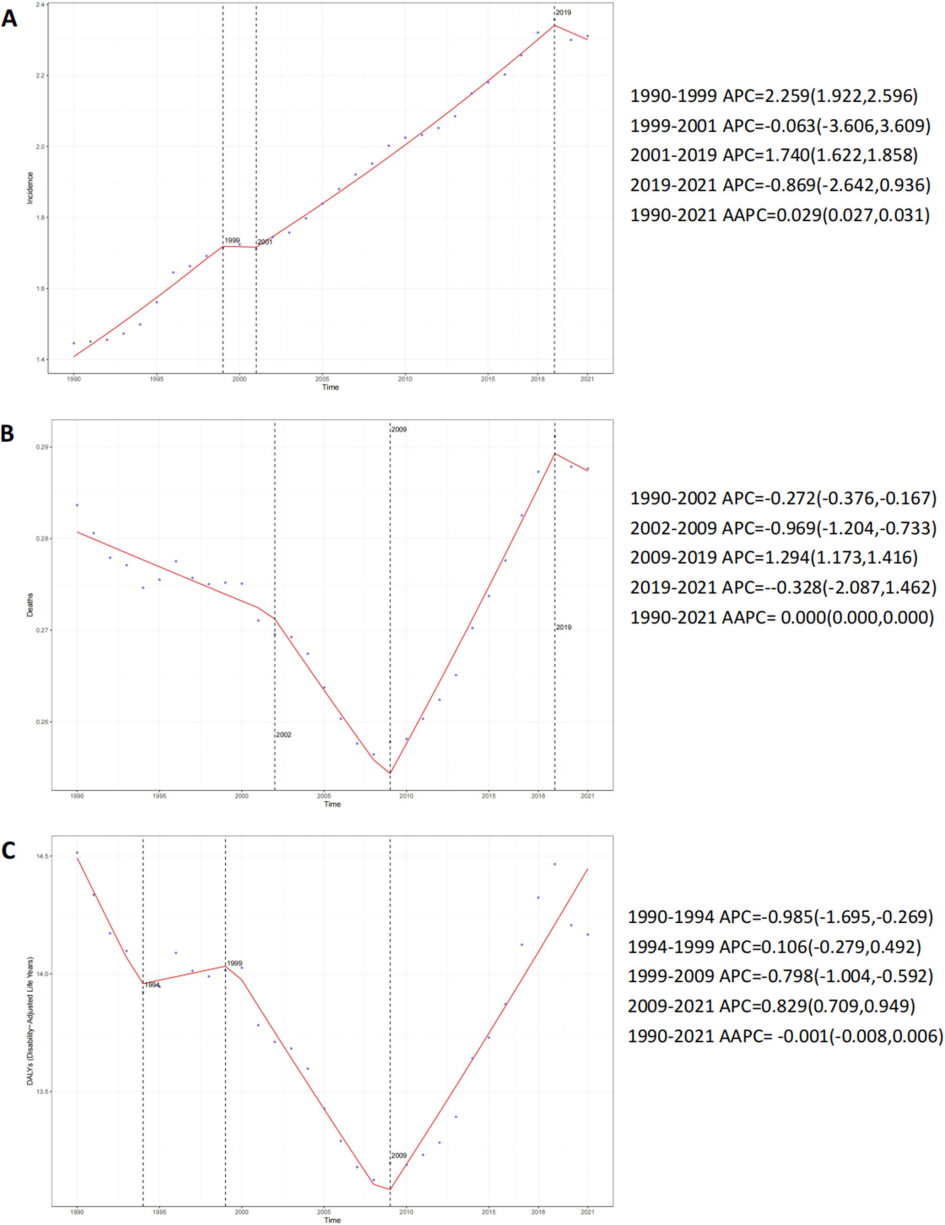
APC and AAPC in the global incidence rate, mortality rate, and DALYs rate of testicular cancer from 1990 to 2021. **(A)** Incidence rate. **(B)** Mortality rate. **(C)** DALYs rate.

Globally, the absolute number of TC incident cases surged by 135.64% (95% UI: 123.26-149.59), from 38,833 in 1990 to 91,507 in 2021. Concurrently, the incidence rate escalated by 59.84% (95% UI: 51.44-69.30), from 1.45 to 2.31 per 100,000 population (**Table 1**, **Figures 1A, B**). Joinpoint analysis identified the most rapid increase in incidence rate between 2001 and 2019 (APC = 1.74%, 95% CI: 1.62-1.86) (**Figure 2A**). The absolute number of deaths increased by 49.48% (95% UI: 36.41-63.63) to 11,388 in 2021. However, the mortality rate demonstrated a marginal overall increase of 1.40% (95% UI: -7.47-10.99) over the study period (**Supplementary Table S4**, **Figures 2B**, **1C, D**). Similarly, DALYs rose by 43.89% (95% UI: 31.27-57.22) to 560,921 in 2021, while the DALY rate experienced a slight decrease of 2.39% (95% UI: -10.95-6.64) (**Supplementary Table S2**, **Figures 2C**, **1E, F**).

**Table 1:** Incidence of testicular cancer between 1990 and 2021 at the global and regional level.

	Rate per 100 000 (95% UI)						
	1990		2021		1990-2021		
Location	Incident cases	Incident rate	Incident cases	Incident rate	Cases change^b^	Rate change^b^	EAPC^a^
Global	38833.49 (37572.49, 40260.79)	1.45 (1.40, 1.50)	91507.38 (87965.92, 95709.72)	2.31 (2.22, 2.42)	135.64 (123.26, 149.59)	59.84 (51.44, 69.30)	1.65 (1.58, 1.72)
SDI							
Low SDI	609.62 (436.72, 784.06)	0.24 (0.17, 0.31)	2199.93 (1796.68, 2609.39)	0.39 (0.32, 0.47)	260.87 (188.30, 360.87)	62.86 (30.11, 108.00)	1.47 (1.20, 1.74)
Low-middle SDI	1990.02 (1665.84, 2338.13)	0.34 (0.28, 0.40)	7520.04 (6729.04, 8437.75)	0.78 (0.70, 0.87)	277.89 (204.48, 379.72)	131.36 (86.42, 193.70)	2.87 (2.69, 3.06)
Middle SDI	4189.41 (3960.79, 4443.02)	0.48 (0.45, 0.51)	20757.02 (19265.04, 22330.50)	1.68 (1.56, 1.81)	395.46 (350.44, 442.16)	252.38 (220.36, 285.59)	4.34 (4.20, 4.48)
High-middle SDI	9724.91 (9159.69, 10359.35)	1.84 (1.73, 1.96)	26679.38 (24613.76, 28913.10)	4.09 (3.77, 4.43)	174.34 (144.66, 203.20)	122.50 (98.43, 145.91)	2.83 (2.71, 2.95)
High SDI	22263.58 (21595.73, 22992.35)	5.14 (4.98, 5.31)	34233.78 (32897.38, 35658.51)	6.27 (6.03, 6.53)	53.77 (46.64, 61.55)	22.12 (16.47, 28.30)	0.72 (0.55, 0.89)
Regions							
Andean Latin America	138.33 (102.68, 180.94)	0.73 (0.54, 0.96)	958.52 (728.76, 1235.76)	2.89 (2.20, 3.73)	592.93 (373.06, 936.51)	294.95 (169.63, 490.79)	4.68 (4.10, 5.27)
Australasia	739.65 (660.44, 831.07)	7.34 (6.56, 8.25)	1262.00 (1089.18, 1443.75)	8.23 (7.11, 9.42)	70.62 (42.11, 100.30)	12.12 (-6.62, 31.62)	0.11 (-0.24, 0.47)
Caribbean	32.78 (29.20, 36.78)	0.19 (0.17, 0.21)	315.64 (265.93, 369.00)	1.34 (1.13, 1.57)	862.85 (681.74, 1084.23)	614.78 (480.33, 779.13)	5.71 (4.50, 6.93)
Central Asia	305.10 (254.06, 373.65)	0.90 (0.75, 1.10)	305.10 (254.06, 373.65)	1.70 (1.44, 2.04)	164.46 (102.25, 242.96)	88.80 (44.38, 144.84)	2.02 (1.62, 2.41)
Central Europe	2597.05 (2425.10, 2840.76)	4.24 (3.96, 4.64)	5100.45 (4614.65, 5688.74)	9.08 (8.22, 10.13)	96.39 (74.48, 121.36)	114.22 (90.32, 141.45)	2.88 (2.69, 3.07)
Central Latin America	937.37 (893.22, 980.34)	1.15 (1.10, 1.21)	6322.08 (5745.31, 6925.56)	5.13 (4.66, 5.62)	574.45 (504.04, 646.54)	343.93 (297.59, 391.38)	5.14 (4.96, 5.33)
Central Sub-Saharan Africa	52.78(36.76,70.06)	0.19(0.13,0.26)	224.12(154.00,311.24)	0.33(0.23,0.46)	324.65(185.86,518.98)	69.22(13.92,146.67)	1.76(1.46,2.07)
East Asia	1996.35 (1669.71, 2341.07)	0.32 (0.27, 0.37)	7089.73 (5597.16, 9024.83)	0.94 (0.74, 1.20)	255.13 (160.07, 377.49)	195.77 (116.60, 297.67)	3.41 (3.20, 3.63)
Eastern Europe	2373.13(2183.45,2546.77)	2.24(2.06,2.41)	4538.16(4150.89,4897.50)	4.72(4.32,5.09)	91.23(69.31,116.80)	110.45(86.33,138.59)	2.26(1.98,2.53)
Eastern Sub-Saharan Africa	219.20(155.08,283.17)	0.23(0.16,0.30)	1067.40(839.18,1323.32)	0.51(0.40,0.63)	386.96(268.43,554.78)	118.16(65.06,193.34)	2.57(2.25,2.88)
High-income Asia Pacific	2392.32(2132.80,2701.63)	2.79(2.49,3.15)	2862.05(2619.33,3130.41)	3.14(2.87,3.43)	19.63(3.48,38.36)	12.43(-2.76,30.02)	0.12(-0.43,0.68)
High-income North America	7762.94(7494.19,8046.56)	5.64(5.45,5.85)	13696.00(13111.72,14330.17)	7.53(7.20,7.87)	76.43(67.22,85.72)	33.37(26.40,40.39)	0.94(0.84,1.04)
North Africa and Middle East	1709.82(1334.94,2164.81)	0.98(0.77,1.25)	12009.08(10179.89,14045.69)	3.71(3.15,4.34)	602.36(423.16,844.01)	277.35(181.07,407.19)	4.95(4.62,5.29)
Oceania	6.66(4.81,8.85)	0.20(0.14,0.26)	16.23(12.95,20.40)	0.23(0.18,0.28)	143.70(79.92,221.75)	14.96(-15.12,51.78)	0.49(0.28,0.71)
South Asia	2475.63(2042.23,2924.54)	0.44(0.36,0.51)	8348.89(7202.42,9574.41)	0.89(0.77,1.02)	237.24(162.63,338.05)	103.68(58.61,164.56)	2.34(2.02,2.65)
Southeast Asia	782.98(690.70,898.61)	0.34(0.30,0.39)	3181.29(2620.61,3811.10)	0.91(0.75,1.09)	306.31(225.75,390.02)	168.68(115.41,224.03)	3.09(3.01,3.17)
Southern Latin America	858.17(736.35,1001.49)	3.54(3.04,4.13)	3566.05(3111.04,4086.60)	10.78(9.41,12.36)	315.54(248.39,406.28)	204.80(155.55,271.36)	3.78(3.42,4.14)
Southern Sub-Saharan Africa	109.91(90.18,128.64)	0.43(0.35,0.51)	308.42(266.28,355.96)	0.79(0.68,0.91)	180.61(129.32,254.32)	81.98(48.71,129.78)	2.11(1.92,2.29)
Tropical Latin America	545.24(510.09,586.26)	0.72(0.68,0.78)	2906.86(2645.15,3161.16)	2.61(2.38,2.84)	433.13(379.16,494.08)	261.74(225.12,303.09)	4.42(4.32,4.53)
Western Europe	12684.73 (12105.15, 13313.37)	6.78 (6.47, 7.11)	16570.80 (15453.79, 17835.56)	7.71 (7.19, 8.30)	30.64 (20.22, 42.63)	13.78 (4.71, 24.23)	0.73 (0.46, 1.00)
Western Sub-Saharan Africa	113.35(91.52,135.81)	0.12(0.10,0.14)	356.72(275.74,444.81)	0.15(0.12,0.19)	214.71(145.14,310.16)	26.38(-1.56,64.71)	0.41(0.21,0.61)

EAPC, estimated annual percentage change; SDI, sociodemographic Index; UI, uncertainty interval.

a EAPC is expressed as 95% confidence interval. b Change shows the percentage change.

### 3.2 Stark regional and national disparities in TC burden

**Figure 3:**
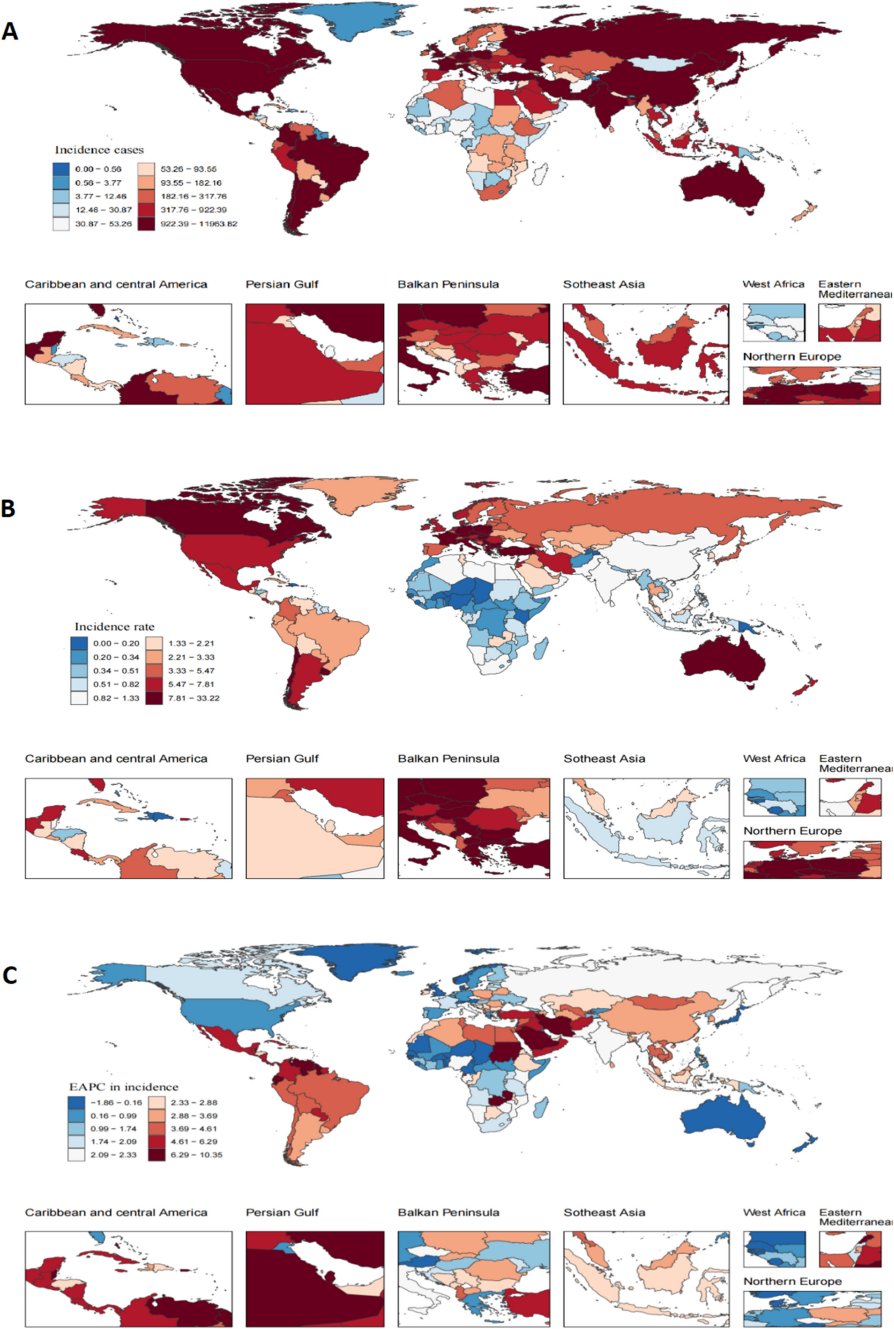
Testicular cancer incidence in 204 countries and territories in 2021. **(A)** Number of incidence cases. **(B)** Incidence rate. **(C)** EAPC in incidence rate from 1990 to 2021.

**Figure 4:**
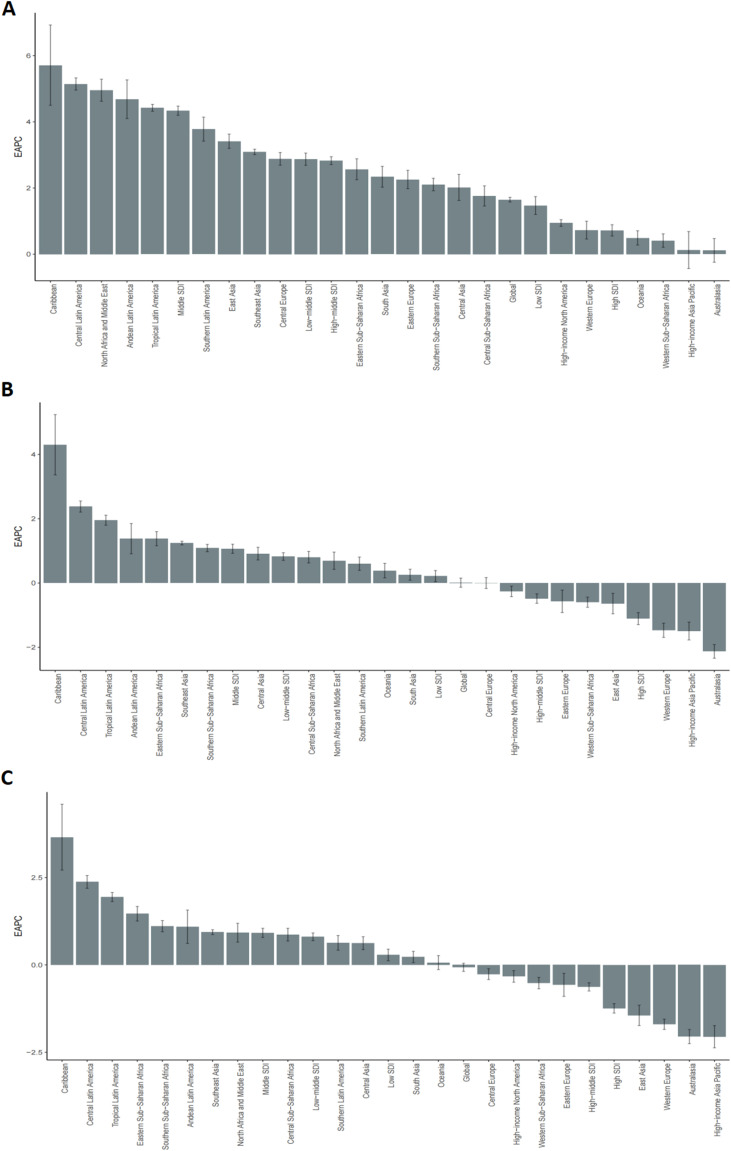
EAPC changes in testicular cancer incidence rate, mortality rate, and DALYs rate from 1990 to 2021 at the global level, across five SDI regions, and in 21 GBD regions. **(A)** Incidence rate. **(B)** Mortality rate. **(C)** DALYs rate.

**Figure 5:**
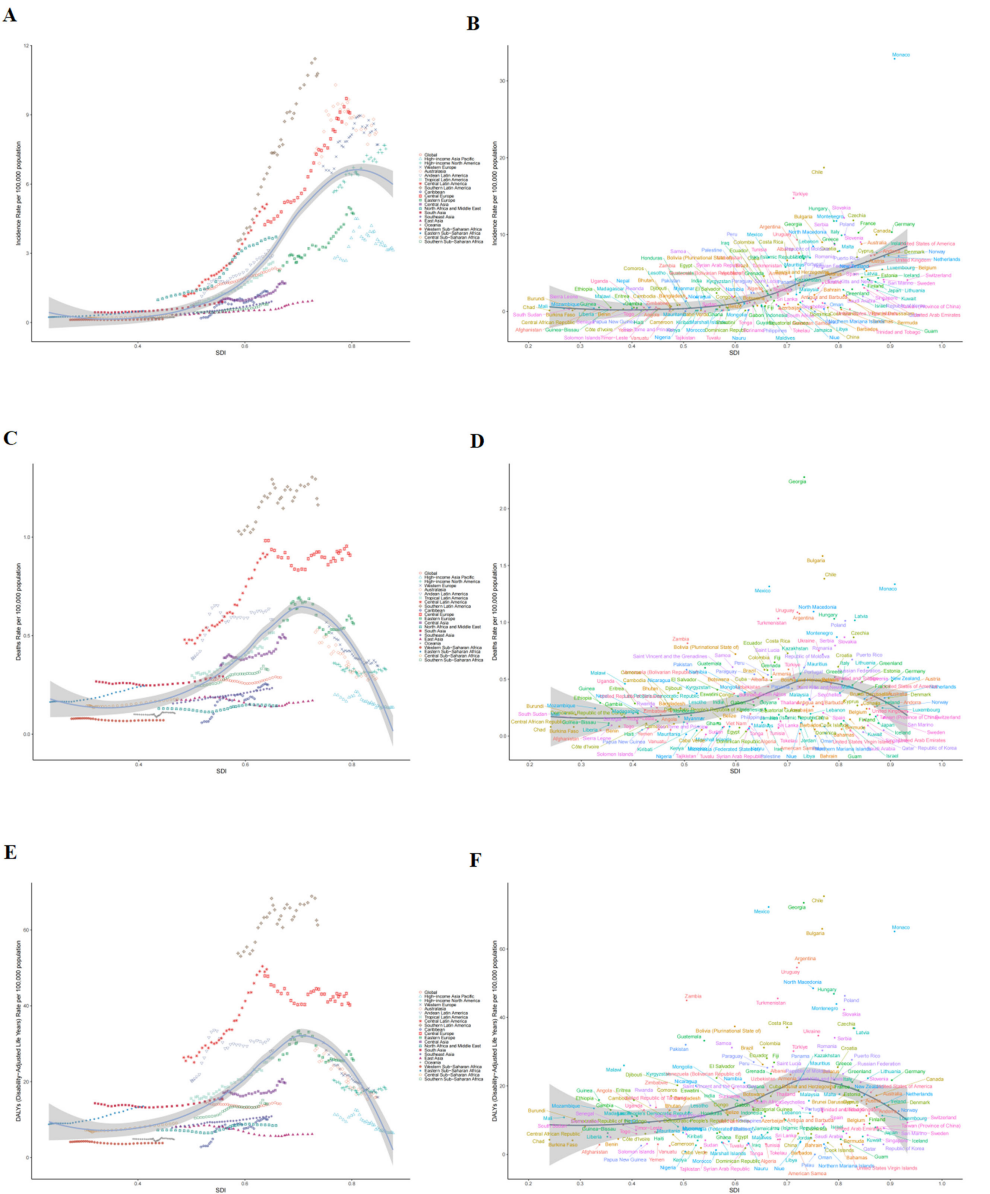
The relationship between testicular cancer incidence rate, mortality rate, and DALYs rate with SDI. **(A)** Global and regional testicular cancer incidence rates from 1990 to 2021. **(B)** Testicular cancer incidence rates in 204 countries in 2021. **(C)** Global and regional testicular cancer mortality rates from 1990 to 2021. **(D)** Testicular cancer mortality rates in 204 countries in 2021. **(E)** Global and regional testicular cancer DALYs rates from 1990 to 2021. **(F)** Testicular cancer DALYs rates in 204 countries in 2021.

Significant heterogeneity in TC burden and its temporal trends was evident across SDI regions, GBD regions, and individual nations (**Table 1**, **Supplementary Tables S2**, **S4**, **Figures 3**–**5**).

Incidence Hotspots and Fastest Increases: Middle-SDI regions bore the brunt of the increasing TC burden, exhibiting the highest EAPC in incidence rate (4.34%, 95% CI: 4.20-4.48). Among GBD regions, the Caribbean displayed the most accelerated rise in incidence rate (EAPC = 5.71%, 95% CI: 4.50-6.93). Nationally, while the USA recorded the highest absolute number of cases in 2021 (11,845), Monaco registered the highest incidence rate (32.89 per 100,000). Qatar demonstrated the most dramatic surge in incidence rate from 1990 to 2021 (EAPC = 10.25, 95% CI: 8.74-11.79) (**Supplementary Table S1**, **Figure 3**).

Divergent Mortality and DALY Trends: High-SDI regions achieved notable reductions in mortality rate (EAPC = -1.11%) and DALY rate (EAPC = -1.24%). In stark contrast, middle-SDI regions faced the most substantial increases in both mortality rate (EAPC = 1.07%) and DALY rate (EAPC = 0.92%). The Caribbean also led GBD regions in mortality rate (EAPC = 4.30%) and DALY rate (EAPC = 3.65%) escalations. India reported the highest absolute number of TC deaths and DALYs in 2021. Georgia and Chile presented the highest mortality rate and DALY rate at the national level, respectively (**Supplementary Tables S3**, **S5**, **Supplementary Figures S1**, **S2**, **S4**).

### 3.3 Age-specific patterns: a young man’s disease

**Figure 6:**
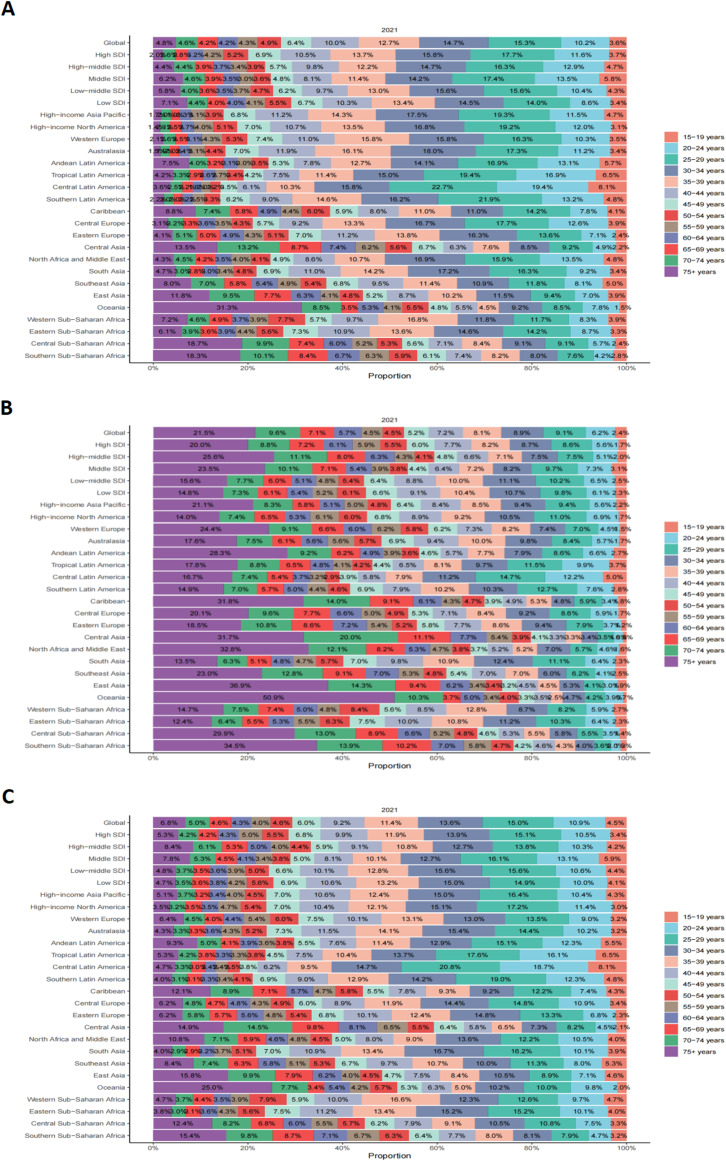
Age-specific percentage of testicular cancer incidence rate, mortality rate, and DALYs rate in 2021. **(A)** Incidence rate. **(B)** Mortality rate. **(C)** DALYs rate.

TC incidence rose across all analyzed age groups between 1990 and 2021, with the 20–24 year age cohort experiencing the largest percentage increase (68.77%). The incidence peak in 2021 was observed in the 25–29 year age group (ASIR 5.58 per 100,000) (**Figure 6A**, **Supplementary Figure 3A**, **S5B**). Mortality rates generally declined across most age groups, with the exception of a slight increase in the 20–24 year cohort (3.03%). The highest mortality burden consistently remained among men aged ≥75 years (**Figure 6B**, **Supplementary Figure S3B**, **S5D**). DALY patterns mirrored these age-specific trends (**Figure 6C**, **Supplementary Figure S3C**, **S5F**).

### 3.4 Drivers of burden change: population dynamics and epidemiological shifts

**Figure 7:**
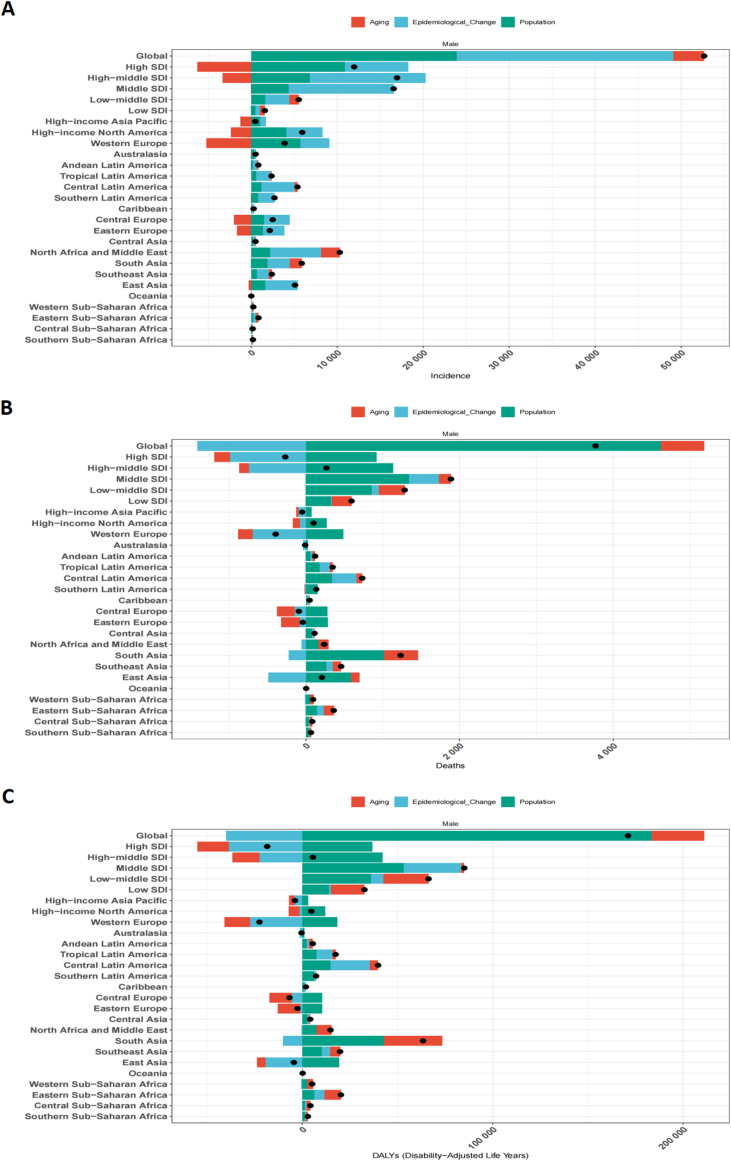
Combined effects of population aging, population growth, and epidemiological changes on testicular cancer from 1990 to 2021 at the global level, across five SDI regions, and in 21 GBD regions. The black dots represent the total change contributed by the three components. A positive value for each component indicates a positive contribution, while a negative value indicates a negative contribution. **(A)** Number of incidence cases. **(B)** Number of death cases. **(C)** Number of DALYs cases.

Decomposition analysis revealed that, globally, epidemiological changes (contributing 47.79%) and population growth (45.34%) were the predominant drivers of the increase in absolute TC incident cases from 1990 to 2021. Population aging played a lesser role (6.87%). For mortality and DALYs, population growth was a key factor increasing absolute numbers, whereas epidemiological shifts (likely reflecting improved survival due to treatment advances) contributed to a decrease in age-standardized rates in many regions (**Figure 7**, **Supplementary Tables S6-S8**).

### 3.5 Future outlook: projections to 2035

**Figure 8:**
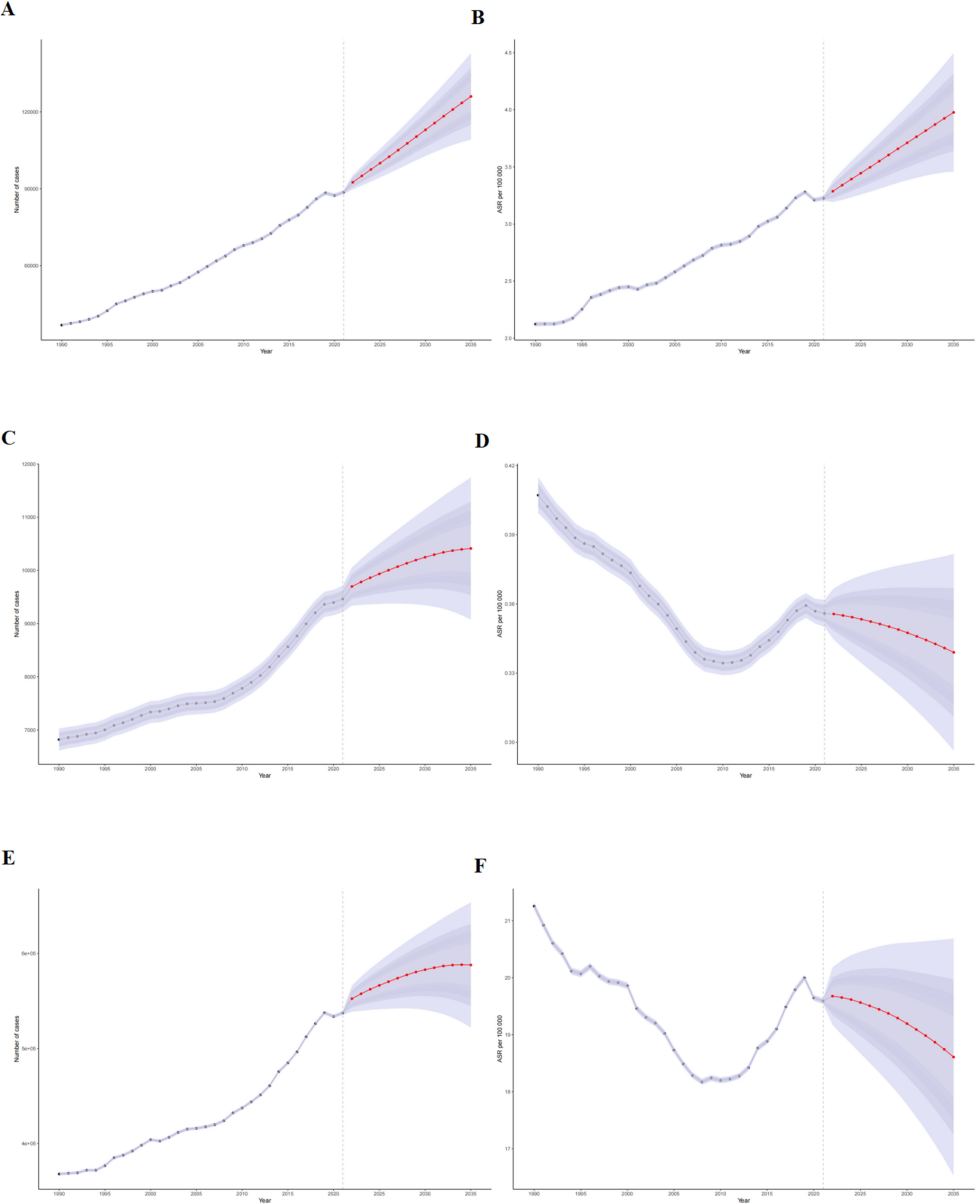
BAPC model projections of testicular cancer incidence, deaths, and DALYs along with their corresponding age-standardized rates through 2035. **(A)** Number of incidence cases. **(B)** Age-standardized incidence rate. **(C)** Number of death cases. **(D)** Age-standardized mortality rate. **(E)** Number of DALYs. **(F)** Age-standardized DALYs rate.

The BAPC model forecasts a continued escalation in the global number of TC incident cases, projected to reach 126,020 (95% UI: 109,145-142,894) by 2035, with the age-standardized incidence rate (ASIR) anticipated to rise to 4.05 per 100,000. Absolute deaths and DALYs are also projected to increase. However, age-standardized mortality rate (ASMR) and age-standardized DALY rate (ASDR) are forecasted to exhibit a slight downward trend globally by 2035 (**Figure 8**), suggesting ongoing, albeit modest, improvements in TC management relative to population changes and incidence increases.

## 4 Discussion

### 4.1 Global trends of testicular cancer

TC is the most common malignancy among young men aged 15 to 40 years. Considering the critical role of this age group in population growth, effective control of TC is not only essential for patient health but may also have implications for future population trends. Therefore, this study comprehensively analyzed the global epidemiological patterns of TC from 1990 to 2021 and projected trends to 2035, providing data support for the development of targeted prevention and control strategies.

From a global perspective, the incidence of TC increased steadily from 1990 to 2021, with a particularly pronounced rise between 2001 and 2019 (APC = 1.74%). This trend may be associated with the combined effects of demographic changes, improvements in healthcare systems, and established etiological risk factors. Global population growth and the expansion of the young adult male population—the age group at highest risk for TC—have contributed to the increasing number of incident cases, as demonstrated by our decomposition analysis (3). Concurrently, advances in diagnostic capacity, including the widespread use of imaging modalities and serum biomarkers (AFP, hCG, and LDH), have improved case ascertainment and early diagnosis (8). In addition to healthcare-related factors, well-established etiological determinants such as cryptorchidism, genetic susceptibility, and *in utero* hormonal influences remain central to TC development and may partly explain long-term incidence patterns (9). Lifestyle-related factors, including obesity, smoking, and alcohol consumption, have also been associated with increased TC risk (10). Environmental exposures, particularly endocrine-disrupting chemicals (EDCs) such as plasticizers and pesticides, have been proposed as potential contributors by interfering with endocrine development during fetal life, thereby increasing the risk of developing TC (11, 12).

Over the past three decades, TC mortality has shown an overall pattern of initial decline followed by a modest increase, with a peak in 2019. Although the absolute number of TC-related deaths increased by 49.5%, the age-standardized mortality rate rose only slightly (1.4%; EAPC = 0.01%), while the DALYs rate decreased by 2.4%, reflecting substantial improvements in disease management. Advances in treatment, particularly the widespread use of platinum-based chemotherapy (e.g., cisplatin), have markedly improved survival in advanced TC (13). In addition, multidisciplinary treatment strategies, including surgery (such as retroperitoneal lymph node dissection) and radiotherapy, have further contributed to mortality reduction (14). Mortality trends differed across age groups, with declines observed in most groups except those aged 20–24 years, and the greatest reduction seen among individuals aged 60–64 years. This pattern may partly reflect lower exposure to TC-specific risk factors at older ages and the influence of competing causes of death, which can affect mortality attribution (15). The mortality peak observed in 2019 may be associated with delayed diagnosis, suboptimal treatment adherence, and unequal access to healthcare services (16). Regional disparities in medical resource availability, particularly in low- and middle-income countries, likely further contribute to fluctuations in TC mortality (17).

### 4.2 Regional disparities by SDI and GBD regions

From the perspectives of SDI and GBD regions, marked regional disparities in TC incidence and mortality were observed over the past three decades. High-SDI regions, including Western Europe and North America, accounted for the largest number of TC cases (34,234 in 2021), but exhibited relatively modest growth (53.8%) compared with middle-SDI regions (252.4%; EAPC = 4.34%) and the Caribbean (614.8%; EAPC = 5.71%). These differences likely reflect the combined effects of genetic susceptibility, healthcare capacity, and socioeconomic development. Racial and ethnic variation contributes substantially to incidence differences. In the United States, non-Hispanic White men have the highest TC incidence (6.63 per 100,000 men per year), approximately five times that of non-Hispanic Black men (1.27 per 100,000 men per year) (11). Genetic susceptibility and family history are well-established TC risk factors, and population-level differences in genetic background may partly explain these patterns. In high-income regions, more complete cancer registries and widespread use of ultrasound and serum biomarkers have improved case detection and early diagnosis, contributing to higher reported incidence (3). In contrast, the rapid increase in incidence observed in middle-SDI regions and the Caribbean is more likely related to changing risk profiles accompanying urbanization and socioeconomic transition. Shifts toward “Westernized” lifestyles, including dietary changes, increased alcohol consumption, and tobacco or marijuana use, have been associated with elevated TC risk (11, 18). Environmental exposures, such as endocrine-disrupting chemicals, have also been proposed as potential contributors, although current evidence remains limited (11). Substantial regional differences were also evident in TC mortality and DALYs. High-SDI and upper-middle-SDI regions experienced significant declines in mortality, particularly in high-SDI regions (EAPC = −1.11%), reflecting early detection, higher public health awareness, and access to effective treatments, including platinum-based chemotherapy and multidisciplinary care (19). In contrast, mortality continued to rise in low- and middle-SDI regions (middle-SDI regions: EAPC = 1.07%), likely due to delayed diagnosis, limited diagnostic and treatment capacity, and barriers to healthcare access (20, 21). The Caribbean region showed the greatest increase in mortality (EAPC = 4.30%), underscoring severe constraints in healthcare resources. Consistent patterns were observed for DALYs, with reductions in high-SDI regions (EAPC = −1.24%) and increases in middle-SDI regions (EAPC = 0.92%). Differences in healthcare infrastructure, diagnostic availability, and cancer registry completeness likely contribute to cross-national variation and should be considered when interpreting regional comparisons.

### 4.3 National-level patterns and implications

At the national level, TC incidence, mortality, and DALYs varied substantially, reflecting differences in population size, healthcare capacity, and disease management. Countries with the highest TC burden were mainly the United States, China, Turkey, and India. In 2021, the United States reported the largest number of TC cases worldwide (11,845 cases), likely related to its large population, comprehensive cancer registry systems, and high diagnostic coverage (4). The high prevalence of obesity among men in the United States may further contribute to TC risk (19). Several countries, including Qatar, Belize, Ecuador, and Grenada, showed particularly rapid increases in incidence. These trends may be associated with recent socioeconomic transitions, population mobility, and changes in lifestyle and environmental exposures. In terms of mortality, India recorded the highest number of TC deaths in 2021 (1,823 deaths), which may reflect its large population base, regional disparities in healthcare access, and a higher proportion of late-stage diagnoses (20). Notably, Belize experienced marked increases in both mortality and DALYs (EAPC: 6.97 and 6.92, respectively), indicating substantial gaps in TC surveillance, early detection, and treatment capacity. Overall, national-level heterogeneity highlights the strong influence of socioeconomic conditions, healthcare resource allocation, and health awareness on TC burden. These findings underscore the need for context-specific prevention, early detection, and treatment strategies to reduce disparities and effectively mitigate TC burden across countries.

### 4.4 Decomposition analysis and future projections

Our decomposition analysis highlights population growth and epidemiological changes as the primary drivers of the increasing global burden of TC. The contribution of epidemiological changes likely reflects shifts in environmental exposures, genetic susceptibility, and lifestyle factors within populations (21). In contrast, population aging appears to mitigate TC burden in some regions, particularly in high-income countries, as TC incidence predominantly peaks among young men aged 15–40 years, while older age groups exhibit substantially lower incidence rates (4). Overall, global TC burden trends result from the combined effects of population growth, demographic transitions, improvements in diagnostic capacity and healthcare access, changes in established and emerging risk factors, and variability in cancer registry completeness across regions. These findings provide an evidence base for policymakers to prioritize early detection, expand access to standardized treatment, and strengthen cancer surveillance systems, especially in countries with high TC-related mortality and DALYs.

The BAPC model projects that, although the global incidence, mortality, and DALYs of TC will continue to increase through 2035, age-standardized mortality (ASMR) and DALYs rates (ASDR) are expected to decline. This divergence suggests that the increasing absolute number of deaths and DALYs is largely driven by population growth and population aging, whereas declining age-standardized rates likely reflect improvements in early detection and treatment outcomes, a pattern commonly observed in global cancer epidemiology (22). Declining ASMR and ASDR suggest continued improvements in survival, likely driven by advances in platinum-based chemotherapy, surgical techniques, and radiotherapy (13, 14). Given that TC is a highly treatable malignancy, timely diagnosis and standardized management remain key determinants of favorable prognosis (13). While these trends indicate progress in disease control, substantial challenges persist in low- and middle-SDI regions, where limited diagnostic capacity, delayed presentation, and constrained healthcare resources may sustain a disproportionate burden of TC. Future global control efforts should therefore prioritize strengthening early detection and treatment infrastructure in these high-risk settings.

### 4.5 Limitations

This study has several limitations that should be acknowledged. First, the quality and completeness of GBD data vary across countries and regions, particularly in low- and middle-SDI settings where cancer registration systems remain underdeveloped. This may result in underreporting or misclassification, potentially affecting the accuracy of incidence and mortality estimates. In addition, GBD estimates are partially derived from statistical modeling rather than purely registry-based observations, which may introduce uncertainty and should be considered when interpreting cross-national comparisons. Second, this analysis did not explicitly incorporate individual-level risk factors, such as environmental exposures, lifestyle behaviors, genetic predisposition, or ethnicity, which limits the ability to fully explain the observed epidemiological patterns of TC. Third, although the BAPC model is widely used for trend projection, its estimates are subject to uncertainty, especially in regions undergoing rapid social, economic, or healthcare transitions, where future trends may deviate from model assumptions. In addition, disruptions caused by the COVID-19 pandemic may have affected cancer diagnosis, treatment, and reporting in some regions, potentially influencing estimates in the most recent years of the study period. Moreover, long-term projections are inherently uncertain, and future structural changes in healthcare systems or unexpected external events may lead to deviations from model-based estimates.

## 5 Conclusion

The global burden of TC, especially its incidence, has significantly and persistently increased from 1990 to 2021, with projections indicating a continued rise by 2035. Middle-SDI regions and specific areas like the Caribbean are emerging as new frontiers with rapidly escalating TC burden, demanding urgent, targeted attention. While treatment advancements have led to relative improvements in mortality and DALY rates in some areas, the growing absolute number of cases poses a substantial and ongoing challenge to global health systems. Tailored public health strategies emphasizing early detection, equitable access to high-quality care, and comprehensive survivorship programs are critical to stem the rising tide of TC and alleviate its impact on young men worldwide.

## Data Availability

The original contributions presented in the study are included in the article/**Supplementary Material**. Further inquiries can be directed to the corresponding authors.
