# Supplementary material for: Trends and projections of global testicular cancer burden from 1990 to 2035
Source: Front Oncol. 2026 Mar 4;16:1634710. doi: 10.3389/fonc.2026.1634710 (PMC12995744; doi:10.3389/fonc.2026.1634710)

**Trends and Projections of Global Testicular Cancer Burden from 1990 to 2035**

**Supplementary Materials**

**Supplementary Table S1: Incidence of testicular cancer by country and territory, 1990–2021.**

**Supplementary Table S2: DALYs due to testicular cancer at the global and regional levels, 1990–2021.**

**Supplementary Table S3: DALYs due to testicular cancer by country and territory, 1990–2021.**

**Supplementary Table S4: Mortality from testicular cancer at the global and regional levels, 1990–2021.**

**Supplementary Table S5: Mortality from testicular cancer by country and territory, 1990–2021.**

**Supplementary Table S6: Decomposition analysis of testicular cancer incidence rates globally, across five SDI regions and 21 GBD regions.**

**Supplementary Table S7: Decomposition analysis of testicular cancer mortality rates globally, across five SDI regions and 21 GBD regions.**

**Supplementary Table S8: Decomposition analysis of testicular cancer DALYs rates globally, across five SDI regions and 21 GBD regions.**

**Supplementary Figure S1: Testicular cancer mortality in 204 countries and territories in 2021.**

**Supplementary Figure S2: Testicular cancer DALYs in 204 countries and territories in 2021.**

**Supplementary Figure S3: Age-specific proportions of testicular cancer incidence, mortality, and DALYs rates in 1990.**

**Supplementary Figure S4: Association between EAPC, disease burden, and SDI in 2021.**

**Supplementary Figure S5: Temporal trends in testicular cancer across different age groups globally, 1990–2021.**

**Supplementary Table S1: Incidence of testicular cancer by country and territory, 1990–2021.**

**Rate per 100 000 (95%UI)**


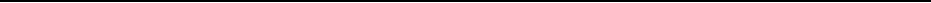

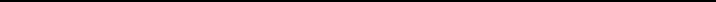

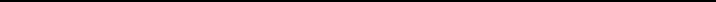


**1990 2021 1990-2021**

**location Incident cases Incident rate Incident cases Incident rate EAPCa**

| Afghanistan | 2.51(1.55,3.98) | 0.05(0.03,0.08) | 35.72(22.79,53.98) | 0.22(0.14,0.34) | 5.34(5.01,5.66) |
| --- | --- | --- | --- | --- | --- |
| Albania | 27.54(19.30,38.85) | 1.62(1.13,2.28) | 60.56(37.35,93.35) | 4.52(2.79,6.96) | 4.29(3.90,4.68) |
| Algeria | 52.29(36.41,71.79) | 0.41(0.28,0.56) | 250.85(166.76,368.61) | 1.12(0.74,1.64) | 3.29(3.23,3.34) |
| American Samoa | 0.00(0.00,0.01) | 0.02(0.01,0.02) | 0.01(0.01,0.02) | 0.05(0.04,0.08) | 4.70(3.70,5.71) |
| Andorra | 1.92(1.21,2.98) | 6.63(4.19,10.32) | 3.23(2.05,4.91) | 7.33(4.65,11.16) | 0.47(0.05,0.88) |
| Angola | 10.84(7.15,15.27) | 0.21(0.14,0.29) | 55.57(37.83,79.58) | 0.35(0.24,0.50) | 1.88(1.61,2.16) |
| Antigua and Barbuda | 0.04(0.04,0.05) | 0.15(0.13,0.17) | 0.56(0.47,0.67) | 1.28(1.07,1.53) | 7.20(5.76,8.66) |
| Argentina | 482.30(386.79,596.61) | 2.98(2.39,3.68) | 1681.51(1408.62,2001.39) | 7.58(6.35,9.02) | 3.24(2.90,3.58) |
| Armenia | 12.86(9.04,17.58) | 0.77(0.54,1.05) | 32.99(23.71,44.84) | 2.29(1.64,3.11) | 3.76(3.33,4.20) |

| Australia | 655.89(574.81,747.84) | 7.82(6.86,8.92) | 1119.61(950.93,1288.42) | 8.79(7.46,10.11) | 0.16(-0.23,0.55) |
| --- | --- | --- | --- | --- | --- |
| Austria | 258.02(221.21,298.46) | 6.91(5.93,7.99) | 287.85(238.67,335.20) | 6.50(5.39,7.56) | 0.07(-0.29,0.42) |
| Azerbaijan | 14.11(8.03,24.17) | 0.39(0.22,0.68) | 47.27(26.80,82.04) | 0.90(0.51,1.56) | 3.48(3.06,3.90) |
| Bahamas | 0.04(0.03,0.04) | 0.03(0.02,0.03) | 0.49(0.39,0.63) | 0.26(0.21,0.33) | 7.44(6.00,8.91) |
| Bahrain | 2.67(1.82,3.66) | 0.91(0.62,1.25) | 20.76(14.22,29.24) | 2.16(1.48,3.04) | 3.69(2.99,4.39) |
| Bangladesh | 184.35(105.50,268.58) | 0.33(0.19,0.48) | 693.24(437.40,1013.12) | 0.86(0.54,1.25) | 3.08(2.76,3.40) |
| Barbados | 0.15(0.13,0.17) | 0.12(0.11,0.14) | 0.82(0.62,1.06) | 0.57(0.43,0.74) | 4.33(2.51,6.18) |
| Belarus | 72.32(60.39,85.94) | 1.48(1.23,1.75) | 190.76(148.27,242.04) | 4.39(3.42,5.58) | 3.53(3.37,3.68) |
| Belgium | 222.92(186.31,264.49) | 4.56(3.81,5.41) | 298.06(244.77,361.23) | 5.28(4.33,6.40) | 0.52(0.31,0.72) |
| Belize | 0.05(0.04,0.06) | 0.05(0.05,0.06) | 1.78(1.47,2.09) | 0.83(0.69,0.98) | 8.71(7.18,10.26) |
| Benin | 4.25(3.14,5.59) | 0.18(0.13,0.24) | 11.43(8.08,15.76) | 0.17(0.12,0.24) | -0.73(-0.97,-0.49) |
| Bermuda | 0.04(0.03,0.05) | 0.14(0.11,0.16) | 0.44(0.35,0.56) | 1.44(1.14,1.83) | 7.64(6.26,9.04) |
| Bhutan | 0.91(0.46,1.44) | 0.27(0.14,0.43) | 3.06(1.82,4.91) | 0.78(0.46,1.25) | 3.72(3.45,3.99) |
| Bolivia (Plurinational State of) | 17.01(11.61,24.66) | 0.54(0.37,0.78) | 115.95(69.74,180.70) | 1.97(1.18,3.06) | 4.46(4.23,4.69) |
| Bosnia and Herzegovina | 40.92(31.29,54.05) | 1.82(1.39,2.40) | 54.95(35.96,75.80) | 3.40(2.23,4.70) | 2.84(2.13,3.55) |
| Botswana | 2.23(1.39,3.28) | 0.35(0.22,0.52) | 8.84(5.37,13.26) | 0.75(0.45,1.12) | 2.35(2.11,2.59) |
| Brazil | 531.71(497.01,572.73) | 0.72(0.68,0.78) | 2819.51(2556.47,3068.91) | 2.62(2.38,2.85) | 4.41(4.30,4.52) |
| Brunei Darussalam | 1.56(0.98,2.32) | 1.14(0.71,1.69) | 5.01(3.25,7.37) | 2.11(1.37,3.10) | 2.20(2.04,2.37) |
| Bulgaria | 221.25(190.60,254.45) | 5.17(4.45,5.95) | 296.15(232.92,366.36) | 9.01(7.09,11.14) | 2.35(1.92,2.79) |
| Burkina Faso | 7.13(4.41,9.84) | 0.15(0.10,0.21) | 15.93(10.51,22.43) | 0.14(0.10,0.20) | -0.57(-0.80,-0.33) |
| Burundi | 6.22(4.08,9.35) | 0.23(0.15,0.35) | 21.05(12.60,33.39) | 0.32(0.19,0.50) | 1.24(0.95,1.52) |
| Cabo Verde | 0.11(0.08,0.14) | 0.06(0.05,0.09) | 0.44(0.28,0.69) | 0.16(0.10,0.24) | 3.20(3.00,3.40) |
| Cambodia | 8.16(5.77,12.10) | 0.17(0.12,0.25) | 43.68(28.63,62.84) | 0.52(0.34,0.75) | 3.96(3.81,4.11) |
| Cameroon | 11.70(8.40,15.94) | 0.23(0.16,0.31) | 37.46(23.46,54.48) | 0.24(0.15,0.34) | -0.42(-0.66,-0.17) |
| Canada | 811.62(689.93,939.24) | 6.04(5.13,6.99) | 1849.56(1566.66,2133.20) | 10.01(8.48,11.54) | 1.89(1.70,2.07) |
| Central African Republic | 2.48(1.58,3.79) | 0.18(0.12,0.28) | 5.75(3.56,9.08) | 0.21(0.13,0.34) | 0.43(0.32,0.55) |
| Chad | 4.39(2.68,6.45) | 0.15(0.09,0.22) | 11.52(7.33,17.53) | 0.13(0.08,0.20) | -0.75(-0.94,-0.56) |
| Chile | 324.92(274.05,384.42) | 4.98(4.20,5.89) | 1732.04(1397.61,2120.37) | 18.72(15.11,22.92) | 4.51(4.05,4.97) |
| China | 1839.33(1521.07,2183.12) | 0.30(0.25,0.36) | 6695.73(5181.39,8656.06) | 0.92(0.71,1.19) | 3.50(3.27,3.72) |
| Colombia | 176.95(152.47,202.16) | 1.10(0.95,1.26) | 1107.58(867.24,1348.05) | 4.61(3.61,5.61) | 5.44(5.05,5.82) |
| Comoros | 0.63(0.34,0.92) | 0.27(0.15,0.40) | 2.41(1.63,3.45) | 0.65(0.44,0.93) | 2.35(1.84,2.86) |
| Congo | 3.06(1.89,4.34) | 0.26(0.16,0.37) | 14.28(8.56,22.82) | 0.53(0.32,0.85) | 2.41(2.27,2.55) |
| Cook Islands | 0.02(0.01,0.03) | 0.17(0.11,0.26) | 0.04(0.03,0.06) | 0.51(0.33,0.74) | 3.60(3.52,3.69) |
| Costa Rica | 23.48(19.85,27.17) | 1.54(1.30,1.78) | 152.27(125.55,183.25) | 6.64(5.48,7.99) | 4.99(4.77,5.20) |
| Côte d'Ivoire | 9.09(6.27,12.85) | 0.15(0.10,0.21) | 36.23(22.78,51.04) | 0.25(0.16,0.35) | 1.52(1.31,1.73) |
| Croatia | 128.56(106.22,157.16) | 5.44(4.49,6.65) | 159.50(125.38,200.30) | 7.81(6.14,9.81) | 1.83(1.42,2.25) |
| Cuba | 17.28(14.38,20.48) | 0.32(0.26,0.38) | 154.85(126.74,187.61) | 2.77(2.26,3.35) | 6.25(4.95,7.57) |
| Cyprus | 10.07(7.35,13.56) | 2.57(1.88,3.46) | 47.31(34.69,62.21) | 7.06(5.18,9.29) | 3.70(3.08,4.33) |
| Czechia | 334.60(282.18,395.16) | 6.70(5.65,7.91) | 620.39(480.74,806.38) | 11.85(9.19,15.41) | 2.28(1.94,2.62) |
| Democratic People's Republic of Korea | 35.94(23.15,53.27) | 0.37(0.24,0.55) | 76.23(48.53,113.68) | 0.58(0.37,0.87) | 1.44(1.16,1.72) |
| Democratic Republic of the Congo | 34.41(22.74,48.03) | 0.18(0.12,0.25) | 136.74(82.22,210.52) | 0.30(0.18,0.46) | 1.63(1.26,2.00) |
| Denmark | 199.78(170.78,230.96) | 7.88(6.74,9.11) | 212.82(176.58,254.40) | 7.30(6.06,8.73) | -0.08(-0.50,0.35) |
| Djibouti | 0.88(0.57,1.29) | 0.40(0.26,0.59) | 5.59(3.43,8.86) | 0.82(0.50,1.31) | 2.28(2.14,2.41) |
| Dominica | 0.02(0.02,0.03) | 0.06(0.05,0.07) | 0.13(0.09,0.19) | 0.39(0.27,0.55) | 6.20(5.41,6.99) |
| Dominican Republic | 2.00(1.50,2.66) | 0.06(0.04,0.08) | 6.87(4.28,10.20) | 0.12(0.08,0.18) | 2.63(1.78,3.48) |
| Ecuador | 8.72(7.57,10.03) | 0.18(0.15,0.20) | 235.14(181.06,301.02) | 2.62(2.02,3.35) | 8.41(6.03,10.84) |
| Egypt | 64.35(49.20,84.93) | 0.23(0.17,0.30) | 539.95(396.26,727.08) | 0.99(0.72,1.33) | 4.54(4.23,4.84) |
| El Salvador | 11.75(9.57,14.26) | 0.45(0.37,0.55) | 72.79(54.18,95.05) | 2.41(1.79,3.14) | 5.99(5.69,6.29) |
| Equatorial Guinea | 0.39(0.25,0.61) | 0.19(0.12,0.30) | 5.91(3.39,9.97) | 0.72(0.41,1.21) | 5.02(4.70,5.35) |
| Eritrea | 3.98(2.76,5.59) | 0.23(0.16,0.33) | 16.63(10.23,25.22) | 0.50(0.31,0.75) | 2.43(2.35,2.50) |
| Estonia | 23.29(18.87,28.46) | 3.18(2.57,3.88) | 27.50(22.81,32.90) | 4.44(3.69,5.32) | 1.11(0.72,1.50) |
| Eswatini | 0.88(0.61,1.25) | 0.23(0.16,0.33) | 2.63(1.68,3.83) | 0.46(0.30,0.68) | 2.17(2.07,2.27) |
| Ethiopia | 55.64(24.62,95.95) | 0.22(0.10,0.38) | 281.00(144.33,400.92) | 0.51(0.26,0.73) | 2.82(2.46,3.18) |
| Federated States of Micronesia | 0.05(0.03,0.07) | 0.09(0.07,0.13) | 0.09(0.06,0.12) | 0.17(0.12,0.24) | 2.09(2.04,2.15) |
| Fiji | 3.30(2.40,4.67) | 0.86(0.62,1.21) | 6.56(4.81,8.91) | 1.40(1.03,1.90) | 1.83(1.41,2.26) |
| Finland | 76.54(66.16,87.04) | 3.15(2.72,3.58) | 110.60(93.31,130.49) | 4.05(3.41,4.77) | 1.52(1.26,1.78) |
| France | 1941.79(1691.32,2234.62) | 6.90(6.01,7.94) | 3300.16(2734.49,3936.33) | 10.25(8.50,12.23) | 1.96(1.64,2.29) |
| Gabon | 1.59(1.04,2.41) | 0.33(0.21,0.50) | 5.88(3.72,9.28) | 0.67(0.43,1.06) | 2.11(1.94,2.28) |
| Gambia | 1.36(0.99,1.83) | 0.28(0.20,0.37) | 5.52(3.58,8.53) | 0.47(0.30,0.72) | 1.19(0.78,1.60) |
| Georgia | 101.92(68.87,159.29) | 3.89(2.63,6.08) | 118.64(92.21,149.59) | 6.86(5.33,8.65) | 1.47(0.78,2.17) |
| Germany | 3482.56(3129.55,3905.43) | 9.02(8.11,10.12) | 4389.86(3727.12,5083.53) | 10.32(8.76,11.95) | 0.41(0.06,0.76) |
| Ghana | 16.34(10.42,25.42) | 0.22(0.14,0.34) | 47.31(28.41,77.37) | 0.28(0.17,0.46) | 0.20(-0.07,0.47) |
| Greece | 403.62(353.14,464.07) | 7.88(6.89,9.06) | 436.03(375.65,506.53) | 8.82(7.60,10.25) | 0.48(-0.05,1.01) |
| Greenland | 1.33(0.95,1.84) | 4.44(3.15,6.12) | 0.85(0.56,1.23) | 2.89(1.92,4.18) | -0.87(-1.55,-0.18) |
| Grenada | 0.06(0.05,0.07) | 0.14(0.12,0.16) | 0.99(0.83,1.17) | 1.88(1.58,2.22) | 8.23(6.92,9.55) |
| Guam | 0.14(0.11,0.17) | 0.19(0.15,0.24) | 0.41(0.33,0.51) | 0.51(0.41,0.63) | 4.61(4.04,5.19) |
| Guatemala | 16.92(15.51,18.43) | 0.41(0.38,0.45) | 150.14(123.46,177.70) | 1.97(1.62,2.33) | 5.75(5.19,6.31) |
| Guinea | 9.67(6.34,13.70) | 0.33(0.22,0.47) | 33.96(21.43,51.10) | 0.52(0.33,0.78) | 1.22(1.09,1.35) |
| Guinea-Bissau | 0.89(0.63,1.25) | 0.18(0.13,0.26) | 1.90(1.30,2.79) | 0.19(0.13,0.28) | -0.27(-0.43,-0.12) |
| Guyana | 0.26(0.21,0.31) | 0.07(0.05,0.08) | 2.71(2.07,3.49) | 0.72(0.55,0.93) | 7.35(5.89,8.84) |
| Haiti | 2.43(1.50,3.69) | 0.08(0.05,0.12) | 12.46(7.32,19.53) | 0.20(0.12,0.31) | 3.52(3.28,3.77) |
| Honduras | 5.37(3.80,7.73) | 0.23(0.16,0.33) | 23.97(12.88,38.81) | 0.49(0.26,0.79) | 2.37(2.24,2.50) |
| Hungary | 326.28(285.08,371.12) | 6.53(5.71,7.43) | 538.48(422.48,666.79) | 11.77(9.23,14.57) | 1.74(1.37,2.12) |
| Iceland | 5.37(4.54,6.39) | 4.21(3.56,5.01) | 7.74(6.38,9.29) | 4.36(3.59,5.23) | 0.64(0.27,1.00) |
| India | 1883.76(1568.76,2225.14) | 0.42(0.35,0.50) | 5977.58(5094.12,6935.60) | 0.83(0.70,0.96) | 2.23(1.85,2.61) |
| Indonesia | 237.21(186.55,300.92) | 0.26(0.20,0.33) | 922.39(600.33,1377.46) | 0.65(0.43,0.98) | 2.84(2.74,2.94) |
| Iran (Islamic Republic of) | 222.17(163.90,306.39) | 0.76(0.56,1.05) | 2509.95(2124.72,2932.82) | 5.78(4.90,6.76) | 8.16(7.47,8.86) |
| Iraq | 57.09(36.61,86.25) | 0.60(0.39,0.91) | 492.28(311.64,747.77) | 2.30(1.46,3.50) | 4.79(4.54,5.05) |
| Ireland | 86.06(74.28,99.73) | 4.79(4.14,5.56) | 186.41(154.77,217.89) | 7.63(6.33,8.92) | 2.52(1.96,3.07) |
| Israel | 43.98(35.99,53.09) | 1.79(1.47,2.16) | 135.10(110.58,164.21) | 2.83(2.31,3.44) | 1.81(1.17,2.45) |
| Italy | 1624.58(1417.50,1838.01) | 5.89(5.14,6.67) | 2886.69(2548.60,3234.02) | 9.91(8.75,11.10) | 2.31(1.87,2.76) |
| Jamaica | 0.89(0.76,1.06) | 0.08(0.07,0.09) | 9.88(7.27,13.43) | 0.71(0.52,0.97) | 6.14(4.56,7.75) |
| Japan | 2261.05(2006.96,2571.55) | 3.66(3.24,4.16) | 2396.68(2173.75,2615.95) | 3.85(3.49,4.20) | -0.07(-0.67,0.52) |
| Jordan | 22.29(15.18,32.83) | 1.14(0.78,1.68) | 370.49(251.99,526.37) | 5.61(3.82,7.98) | 6.29(5.78,6.81) |
| Kazakhstan | 91.51(66.34,127.34) | 1.15(0.84,1.60) | 226.29(153.37,312.78) | 2.46(1.67,3.40) | 2.88(2.51,3.25) |
| Kenya | 6.84(4.18,9.34) | 0.06(0.04,0.08) | 30.63(23.01,40.00) | 0.12(0.09,0.16) | 2.12(1.87,2.37) |
| Kiribati | 0.03(0.02,0.04) | 0.08(0.06,0.12) | 0.08(0.05,0.11) | 0.13(0.09,0.19) | 1.50(1.46,1.54) |
| Kuwait | 18.51(14.81,22.54) | 1.88(1.51,2.29) | 85.62(66.49,108.68) | 3.49(2.71,4.42) | 0.92(-1.26,3.14) |
| Kyrgyzstan | 22.51(16.43,30.25) | 1.03(0.75,1.39) | 47.70(35.12,61.91) | 1.41(1.04,1.83) | 0.85(0.19,1.51) |
| Lao People's Democratic Republic | 3.18(2.13,4.95) | 0.16(0.10,0.24) | 12.75(8.52,18.49) | 0.34(0.23,0.50) | 2.78(2.53,3.03) |
| Latvia | 27.42(22.53,33.06) | 2.22(1.82,2.68) | 41.86(33.28,51.71) | 4.86(3.86,6.00) | 1.81(1.40,2.23) |
| Lebanon | 15.35(9.78,23.48) | 1.03(0.66,1.58) | 193.78(134.95,269.71) | 7.00(4.88,9.74) | 7.00(6.65,7.35) |
| Lesotho | 1.49(1.00,1.99) | 0.21(0.14,0.29) | 3.77(2.59,5.17) | 0.41(0.28,0.57) | 2.19(2.11,2.28) |
| Liberia | 2.45(1.74,3.38) | 0.20(0.14,0.27) | 6.09(3.51,9.67) | 0.22(0.13,0.35) | 0.66(0.42,0.89) |
| Libya | 9.31(6.26,13.50) | 0.42(0.28,0.61) | 41.51(27.16,60.77) | 1.17(0.77,1.72) | 3.87(3.32,4.42) |
| Lithuania | 33.57(28.12,40.24) | 1.93(1.62,2.32) | 52.68(43.01,63.33) | 4.19(3.42,5.04) | 1.76(1.34,2.19) |
| Luxembourg | 13.33(11.62,15.35) | 7.14(6.22,8.22) | 17.74(15.46,20.70) | 5.47(4.76,6.38) | -0.67(-1.02,-0.32) |
| Madagascar | 14.06(10.60,18.67) | 0.24(0.18,0.31) | 53.26(36.49,77.36) | 0.38(0.26,0.55) | 1.25(1.05,1.45) |
| Malawi | 21.01(14.46,28.29) | 0.44(0.30,0.59) | 87.80(58.98,130.21) | 0.93(0.62,1.37) | 2.43(2.28,2.57) |
| Malaysia | 55.98(43.27,73.18) | 0.63(0.49,0.82) | 285.24(220.92,369.31) | 1.73(1.34,2.24) | 3.29(3.09,3.50) |
| Maldives | 0.15(0.10,0.23) | 0.13(0.09,0.20) | 2.63(1.79,3.74) | 0.82(0.56,1.17) | 7.34(6.65,8.03) |
| Mali | 11.93(8.90,15.22) | 0.28(0.21,0.36) | 46.22(30.74,65.69) | 0.39(0.26,0.55) | 0.99(0.80,1.18) |
| Malta | 9.19(7.71,10.81) | 5.02(4.21,5.91) | 17.43(14.57,21.35) | 7.88(6.59,9.65) | 2.15(1.69,2.61) |
| Marshall Islands | 0.02(0.01,0.03) | 0.08(0.05,0.11) | 0.04(0.03,0.06) | 0.14(0.09,0.20) | 2.07(2.00,2.14) |
| Mauritania | 2.86(1.88,4.00) | 0.28(0.18,0.39) | 7.70(4.96,11.23) | 0.36(0.23,0.52) | 0.01(-0.24,0.26) |
| Mauritius | 3.05(2.76,3.37) | 0.56(0.50,0.61) | 13.85(12.22,15.44) | 2.21(1.95,2.46) | 4.53(3.64,5.42) |
| Mexico | 657.83(627.42,689.12) | 1.56(1.49,1.64) | 4456.72(3990.39,4995.01) | 7.06(6.32,7.92) | 5.02(4.84,5.20) |
| Monaco | 3.74(2.49,5.38) | 25.86(17.22,37.16) | 6.05(3.93,9.04) | 32.89(21.37,49.14) | 0.77(0.65,0.89) |
| Mongolia | 4.10(2.78,5.80) | 0.38(0.26,0.54) | 16.60(12.00,21.99) | 1.01(0.73,1.34) | 3.91(3.57,4.26) |
| Montenegro | 18.36(13.34,24.76) | 5.89(4.28,7.94) | 35.96(24.69,49.46) | 11.78(8.09,16.20) | 2.80(2.54,3.07) |
| Morocco | 14.71(9.84,20.98) | 0.12(0.08,0.17) | 52.13(33.06,85.78) | 0.28(0.18,0.46) | 2.73(2.60,2.86) |
| Mozambique | 14.63(9.38,20.69) | 0.23(0.15,0.32) | 63.18(38.56,98.10) | 0.42(0.26,0.66) | 2.13(2.04,2.22) |
| Myanmar | 34.94(24.72,48.76) | 0.18(0.12,0.24) | 97.32(66.05,136.47) | 0.36(0.24,0.50) | 2.29(2.08,2.49) |
| Namibia | 3.35(2.29,4.82) | 0.49(0.33,0.70) | 12.63(7.54,19.70) | 1.07(0.64,1.67) | 2.32(2.17,2.48) |
| Nauru | 0.01(0.00,0.01) | 0.11(0.08,0.15) | 0.01(0.01,0.01) | 0.15(0.11,0.22) | 0.87(0.59,1.14) |
| Nepal | 23.24(11.84,36.12) | 0.24(0.12,0.37) | 71.87(43.38,105.61) | 0.49(0.29,0.71) | 2.28(2.01,2.55) |
| Netherlands | 426.40(373.41,489.82) | 5.78(5.06,6.64) | 608.54(521.84,707.55) | 7.12(6.11,8.28) | 1.05(0.86,1.24) |
| New Zealand | 83.76(69.61,99.75) | 4.96(4.12,5.91) | 142.39(122.46,166.53) | 5.52(4.74,6.45) | -0.28(-0.77,0.22) |
| Nicaragua | 7.74(5.25,11.29) | 0.41(0.28,0.59) | 61.16(37.31,95.31) | 1.86(1.13,2.89) | 5.71(5.42,5.99) |
| Niger | 5.52(3.38,8.15) | 0.14(0.08,0.20) | 12.96(6.66,21.95) | 0.10(0.05,0.18) | -1.52(-1.78,-1.26) |
| Nigeria | 9.00(6.02,12.38) | 0.02(0.01,0.03) | 40.30(27.32,57.50) | 0.04(0.02,0.05) | 2.16(1.88,2.45) |
| Niue | 0.00(0.00,0.00) | 0.17(0.12,0.23) | 0.00(0.00,0.00) | 0.29(0.21,0.40) | 1.87(1.73,2.01) |
| North Macedonia | 40.14(30.36,51.34) | 4.01(3.03,5.13) | 93.55(67.36,123.03) | 8.47(6.10,11.14) | 3.25(2.82,3.69) |
| Northern Mariana Islands | 0.04(0.03,0.07) | 0.19(0.12,0.28) | 0.08(0.06,0.11) | 0.32(0.24,0.41) | 1.51(0.51,2.52) |
| Norway | 146.40(130.14,164.82) | 6.98(6.20,7.85) | 197.18(177.13,216.58) | 7.21(6.47,7.91) | -0.51(-1.50,0.49) |
| Oman | 2.88(1.80,4.39) | 0.25(0.15,0.38) | 30.87(20.86,44.39) | 1.07(0.72,1.53) | 5.51(4.99,6.04) |
| Pakistan | 383.37(283.96,483.95) | 0.66(0.49,0.83) | 1603.14(1091.78,2170.72) | 1.33(0.91,1.80) | 2.13(2.08,2.19) |
| Palau | 0.00(0.00,0.00) | 0.00(0.00,0.00) | 0.00(0.00,0.00) | 0.00(0.00,0.00) | 0.89(0.64,1.14) |
| Palestine | 8.48(5.52,12.80) | 0.82(0.54,1.24) | 63.33(45.86,87.89) | 2.42(1.75,3.36) | 3.37(2.83,3.91) |
| Panama | 6.92(5.96,8.09) | 0.57(0.49,0.67) | 61.60(49.24,75.37) | 2.85(2.28,3.48) | 4.94(4.40,5.48) |
| Papua New Guinea | 1.38(0.62,2.44) | 0.06(0.03,0.11) | 5.32(3.25,7.97) | 0.10(0.06,0.15) | 1.18(0.98,1.37) |
| Paraguay | 13.53(10.25,17.37) | 0.67(0.50,0.85) | 87.35(58.74,128.23) | 2.41(1.62,3.54) | 4.80(4.56,5.05) |
| Peru | 112.60(78.49,152.62) | 1.05(0.73,1.42) | 607.43(414.65,871.31) | 3.33(2.27,4.77) | 4.08(3.79,4.37) |
| Philippines | 132.44(110.45,152.32) | 0.42(0.35,0.48) | 358.78(286.17,448.16) | 0.62(0.50,0.78) | 0.97(0.70,1.25) |
| Poland | 734.40(661.66,812.46) | 3.95(3.56,4.37) | 1848.54(1609.69,2114.25) | 9.99(8.70,11.42) | 3.38(3.16,3.59) |
| Portugal | 143.37(124.56,166.42) | 2.93(2.54,3.40) | 235.37(199.06,277.31) | 4.67(3.95,5.50) | 1.62(1.28,1.96) |
| Puerto Rico | 7.20(6.08,8.44) | 0.41(0.35,0.48) | 99.74(79.01,125.00) | 6.38(5.05,7.99) | 8.14(6.93,9.36) |
| Qatar | 0.74(0.49,1.06) | 0.25(0.17,0.36) | 41.37(27.60,57.02) | 1.97(1.31,2.72) | 10.25(8.74,11.79) |
| Republic of Korea | 108.10(82.87,134.90) | 0.49(0.37,0.61) | 381.25(276.76,513.47) | 1.47(1.07,1.98) | 3.48(3.19,3.78) |
| Republic of Moldova | 39.45(35.08,44.10) | 1.86(1.65,2.08) | 58.05(49.75,66.72) | 3.39(2.91,3.90) | 2.07(1.63,2.52) |
| Romania | 291.11(253.75,333.21) | 2.53(2.20,2.89) | 515.97(406.79,639.74) | 5.60(4.42,6.95) | 3.24(2.86,3.63) |
| Russian Federation | 1723.08(1637.76,1821.44) | 2.44(2.32,2.58) | 3563.43(3246.38,3828.33) | 5.28(4.81,5.68) | 2.33(2.00,2.67) |
| Rwanda | 9.01(6.00,13.57) | 0.26(0.17,0.39) | 35.77(23.52,53.39) | 0.55(0.36,0.83) | 2.74(2.34,3.13) |
| Saint Kitts and Nevis | 0.03(0.02,0.03) | 0.13(0.12,0.15) | 0.45(0.33,0.63) | 1.54(1.13,2.14) | 7.12(5.78,8.46) |
| Saint Lucia | 0.11(0.10,0.13) | 0.17(0.15,0.19) | 2.02(1.61,2.45) | 2.27(1.82,2.76) | 8.07(6.55,9.62) |
| Saint Vincent and the Grenadines | 0.05(0.05,0.06) | 0.10(0.08,0.11) | 0.65(0.55,0.76) | 1.12(0.95,1.30) | 7.13(5.81,8.47) |
| Samoa | 1.02(0.65,1.57) | 1.15(0.73,1.77) | 2.16(1.37,3.35) | 1.96(1.24,3.05) | 1.54(1.44,1.63) |
| San Marino | 0.53(0.36,0.72) | 4.57(3.11,6.17) | 0.61(0.34,0.95) | 3.85(2.16,6.00) | -0.09(-0.56,0.38) |
| Sao Tome and Principe | 0.10(0.07,0.15) | 0.17(0.11,0.24) | 0.36(0.22,0.59) | 0.34(0.20,0.54) | 1.80(1.55,2.04) |
| Saudi Arabia | 23.26(14.73,34.91) | 0.26(0.17,0.39) | 457.47(279.56,717.49) | 2.08(1.27,3.26) | 7.70(7.25,8.15) |
| Senegal | 8.97(6.51,12.11) | 0.24(0.18,0.33) | 23.18(15.68,34.25) | 0.29(0.20,0.43) | 0.16(-0.05,0.37) |
| Serbia | 192.22(114.24,309.08) | 4.05(2.41,6.52) | 386.49(231.85,601.39) | 8.68(5.21,13.51) | 2.85(2.62,3.08) |
| Seychelles | 0.27(0.20,0.36) | 0.73(0.54,1.00) | 0.74(0.54,0.97) | 1.33(0.97,1.74) | 2.29(2.04,2.55) |
| Sierra Leone | 3.65(2.47,5.05) | 0.18(0.12,0.25) | 8.19(5.07,11.59) | 0.19(0.12,0.26) | -0.23(-0.60,0.13) |
| Singapore | 21.62(17.83,25.89) | 1.41(1.16,1.69) | 79.11(60.31,101.63) | 2.71(2.07,3.49) | 1.95(1.47,2.42) |
| Slovakia | 149.04(99.78,217.56) | 5.78(3.87,8.43) | 322.85(198.71,504.02) | 12.18(7.50,19.02) | 2.91(2.71,3.11) |
| Slovenia | 51.08(43.90,59.59) | 5.33(4.58,6.22) | 92.82(74.02,112.59) | 9.03(7.20,10.95) | 2.14(1.81,2.48) |
| Solomon Islands | 0.11(0.06,0.19) | 0.06(0.03,0.11) | 0.41(0.26,0.60) | 0.12(0.07,0.17) | 1.93(1.80,2.06) |
| Somalia | 7.82(3.88,13.19) | 0.19(0.09,0.32) | 24.30(10.29,43.51) | 0.22(0.09,0.39) | 0.63(0.56,0.71) |
| South Africa | 92.48(74.81,108.73) | 0.52(0.42,0.61) | 250.25(215.20,290.61) | 0.90(0.77,1.04) | 2.03(1.78,2.29) |
| South Sudan | 7.04(4.20,10.95) | 0.23(0.13,0.35) | 14.32(7.37,22.46) | 0.30(0.15,0.46) | 0.87(0.66,1.07) |
| Spain | 626.54(540.41,712.35) | 3.30(2.84,3.75) | 823.12(681.91,988.08) | 3.70(3.06,4.44) | 0.42(-0.05,0.89) |
| Sri Lanka | 32.88(24.61,43.14) | 0.38(0.29,0.50) | 112.67(76.04,158.39) | 1.05(0.71,1.47) | 3.25(3.13,3.36) |
| Sudan | 10.96(7.27,16.43) | 0.11(0.07,0.16) | 158.44(100.27,240.22) | 0.72(0.45,1.09) | 6.59(6.40,6.79) |
| Suriname | 0.33(0.23,0.43) | 0.17(0.12,0.22) | 2.27(1.58,3.25) | 0.79(0.55,1.14) | 4.80(3.80,5.81) |
| Sweden | 209.43(182.28,243.97) | 4.94(4.30,5.76) | 216.09(177.75,261.03) | 4.14(3.41,5.00) | 0.65(-0.05,1.34) |
| Switzerland | 228.08(197.20,267.25) | 6.74(5.83,7.90) | 182.16(149.53,216.19) | 4.09(3.36,4.86) | -1.84(-2.31,-1.36) |
| Syrian Arab Republic | 20.28(13.70,29.49) | 0.31(0.21,0.45) | 70.92(49.52,104.08) | 1.05(0.73,1.54) | 3.98(3.38,4.59) |
| Taiwan (Province of China) | 121.09(104.69,138.85) | 1.15(0.99,1.32) | 317.76(273.78,367.92) | 2.72(2.34,3.15) | 2.65(2.25,3.05) |
| Tajikistan | 0.65(0.42,0.99) | 0.02(0.02,0.04) | 1.74(0.92,2.89) | 0.03(0.02,0.06) | 1.12(0.75,1.49) |
| Thailand | 155.63(110.86,212.16) | 0.55(0.39,0.75) | 734.73(529.38,1020.53) | 2.28(1.64,3.17) | 4.54(4.32,4.76) |
| Timor-Leste | 0.66(0.42,0.96) | 0.16(0.10,0.24) | 2.07(1.43,3.02) | 0.29(0.20,0.43) | 1.93(1.52,2.34) |
| Togo | 3.91(2.80,5.23) | 0.22(0.16,0.29) | 10.02(6.08,14.63) | 0.24(0.15,0.36) | -0.40(-0.69,-0.12) |
| Tokelau | 0.00(0.00,0.00) | 0.14(0.09,0.20) | 0.00(0.00,0.00) | 0.32(0.23,0.43) | 2.65(2.51,2.79) |
| Tonga | 0.06(0.04,0.07) | 0.11(0.08,0.15) | 0.11(0.08,0.17) | 0.22(0.15,0.32) | 1.96(1.83,2.08) |
| Trinidad and Tobago | 0.68(0.60,0.76) | 0.11(0.10,0.13) | 7.73(5.66,10.17) | 1.11(0.81,1.46) | 7.63(6.01,9.27) |
| Tunisia | 27.23(19.60,37.48) | 0.64(0.46,0.89) | 121.59(78.92,178.42) | 2.06(1.34,3.03) | 3.74(3.59,3.89) |
| Turkey | 1109.04(762.64,1506.09) | 3.81(2.62,5.17) | 6184.54(4625.01,7853.15) | 14.75(11.03,18.73) | 4.96(4.57,5.35) |
| Turkmenistan | 12.44(9.31,16.36) | 0.68(0.51,0.90) | 85.35(52.82,135.69) | 3.24(2.00,5.15) | 3.66(2.82,4.50) |
| Tuvalu | 0.00(0.00,0.01) | 0.08(0.06,0.12) | 0.01(0.01,0.02) | 0.17(0.12,0.24) | 2.23(2.13,2.33) |
| Uganda | 21.56(14.61,29.88) | 0.25(0.17,0.35) | 108.41(72.34,160.60) | 0.51(0.34,0.75) | 1.77(1.54,2.00) |
| Ukraine | 453.99(326.86,569.90) | 1.86(1.34,2.34) | 603.88(402.14,839.79) | 3.03(2.02,4.21) | 1.50(1.24,1.76) |
| United Arab Emirates | 19.63(12.28,30.57) | 1.62(1.01,2.53) | 213.49(147.81,294.84) | 3.07(2.13,4.24) | 2.74(2.05,3.43) |
| United Kingdom | 2510.09(2425.07,2604.82) | 9.02(8.71,9.36) | 1950.07(1862.38,2031.11) | 5.86(5.59,6.10) | -0.97(-1.23,-0.70) |
| United Republic of Tanzania | 39.64(27.72,56.79) | 0.32(0.22,0.45) | 155.24(108.19,213.12) | 0.55(0.38,0.75) | 1.77(1.60,1.93) |
| United States of America | 6949.81(6720.07,7167.54) | 5.60(5.42,5.78) | 11845.37(11296.60,12404.34) | 7.25(6.91,7.59) | 0.82(0.70,0.94) |
| United States Virgin Islands | 0.03(0.02,0.04) | 0.06(0.04,0.08) | 0.13(0.08,0.18) | 0.30(0.19,0.44) | 6.86(6.35,7.38) |
| Uruguay | 50.91(40.98,63.11) | 3.33(2.68,4.13) | 152.30(123.66,187.94) | 9.33(7.58,11.51) | 3.25(3.00,3.51) |
| Uzbekistan | 45.00(31.28,63.78) | 0.43(0.30,0.62) | 230.31(167.53,318.88) | 1.35(0.98,1.87) | 3.89(3.33,4.45) |
| Vanuatu | 0.05(0.03,0.08) | 0.06(0.03,0.10) | 0.15(0.10,0.21) | 0.10(0.07,0.14) | 1.15(1.05,1.24) |
| Venezuela (Bolivarian Republic of) | 30.42(26.66,34.20) | 0.32(0.28,0.36) | 235.85(177.42,311.22) | 1.83(1.38,2.42) | 6.57(5.44,7.71) |
| Viet Nam | 117.30(81.72,163.50) | 0.35(0.25,0.49) | 590.02(397.85,850.33) | 1.18(0.80,1.70) | 3.86(3.72,3.99) |
| Yemen | 5.14(2.47,8.75) | 0.07(0.04,0.13) | 62.83(36.54,97.97) | 0.37(0.22,0.58) | 6.24(5.68,6.82) |
| Zambia | 10.08(7.26,13.37) | 0.26(0.19,0.34) | 166.88(98.85,262.16) | 1.73(1.02,2.71) | 7.72(6.44,9.01) |
| Zimbabwe | 9.48(6.92,12.36) | 0.19(0.14,0.24) | 30.29(19.93,43.34) | 0.40(0.27,0.58) | 2.26(1.66,2.86) |

Abbreviations: EAPC, estimated annual percentage change; UI, uncertainty interval.

a EAPC is expressed as 95% confidence interval

**Supplementary Table S2: DALYs due to testicular cancer at the global and regional levels, 1990–2021.**

**Rate per 100 000(95%UI)**


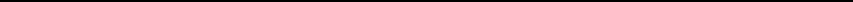

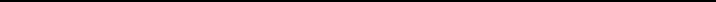

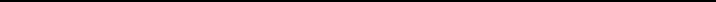

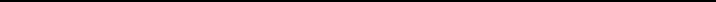


**1990 2021 1990-2021**

**Location Number of DALYs DALYs rate Number of DALYs DALYs rate Cases change^b^ Rate change^b^ EAPC^a^**

Global 389816.96(365898.80,413488.79) 14.51(13.62,15.40) 560921.46(528325.48,597363.24) 14.17(13.34,15.09) 43.89(31.27,57.22) -2.39(-10.95,6.64) -0.07(-0.19,0.05)

SDI

Low SDI 22044.15(15761.60,28534.49) 8.74(6.25,11.31) 54695.02(43961.86,65247.48) 9.78(7.86,11.67) 148.12(96.26,220.40) 11.98(-11.43,44.60) 0.28(0.12,0.45)

Low-middle SDI 63854.02(52805.11,75363.03) 10.80(8.93,12.75) 130174.29(114504.69,148363.09) 13.48(11.86,15.36) 103.86(60.11,163.41) 24.81(-1.97,61.27) 0.80(0.69,0.92)

Middle SDI 98392.25(92244.95,104763.89) 11.22(10.52,11.94) 183469.83(169718.20,197745.08) 14.88(13.76,16.04) 86.47(68.93,104.84) 32.62(20.15,45.69) 0.92(0.79,1.05)

High-middle SDI 103899.90(97041.25,110646.48) 19.63(18.34,20.91) 109438.27(101409.66,119084.67) 16.77(15.54,18.25) 5.33(-4.74,16.53) -14.57(-22.74,-5.49) -0.63(-0.75,-0.51)

High SDI 101043.07(96723.76,105564.72) 23.31(22.32,24.36) 82584.98(76395.96,90296.11) 15.13(14.00,16.55) -18.27(-22.45,-13.80) -35.09(-38.41,-31.54) -1.24(-1.38,-1.11)

Regions

Andean Latin America 3826.43(2816.79,5032.30) 20.27(14.92,26.65) 9347.11(7197.43,11766.14) 28.22(21.73,35.52) 144.28(70.86,249.02) 39.23(-2.62,98.93) 1.09(0.61,1.57)

Australasia 2847.67(2613.37,3078.38) 28.28(25.95,30.57) 2442.98(2118.41,2852.67) 15.94(13.82,18.61) -14.21(-24.58,-1.30) -43.63(-50.44,-35.14) -2.05(-2.25,-1.85)

Caribbean 487.17(439.74,553.95) 2.80(2.52,3.18) 2353.74(2006.70,2753.25) 10.03(8.55,11.73) 383.15(296.00,481.34) 258.67(193.98,331.57) 3.65(2.71,4.60)

Central Asia 5200.40(4353.55,6223.25) 15.33(12.83,18.34) 9344.79(7973.96,11108.53) 19.66(16.78,23.37) 79.69(40.21,132.37) 28.28(0.10,65.89) 0.62(0.44,0.81)

Central Europe 29347.84(27607.96,31421.21) 47.91(45.07,51.29) 22588.13(20501.06,24830.71) 40.22(36.50,44.21) -23.03(-31.32,-14.88) -16.05(-25.09,-7.15) -0.27(-0.42,-0.11)

Central Latin America 21418.17(20602.41,22215.94) 26.38(25.38,27.37) 61218.68(55574.13,66761.62) 49.64(45.06,54.13) 185.83(157.68,213.30) 88.13(69.61,106.22) 2.38(2.19,2.56)

Central Sub-Saharan Africa 1961.95(1350.04,2627.34) 7.20(4.96,9.64) 6213.36(4269.27,8684.97) 9.09(6.25,12.71) 216.69(113.14,369.34) 26.20(-15.06,87.03) 0.87(0.68,1.05)

East Asia 51002.73(42182.15,60107.65) 8.13(6.73,9.59) 46545.56(36155.77,58273.92) 6.18(4.80,7.74) -8.74(-34.13,24.16) -23.99(-45.14,3.41) -1.45(-1.74,-1.15)

Eastern Europe 27265.96(24778.26,29546.01) 25.76(23.41,27.91) 24717.89(21766.06,27656.47) 25.70(22.63,28.75) -9.35(-21.99,6.28) -0.23(-14.15,16.96) -0.57(-0.90,-0.24)

Eastern Sub-Saharan Africa 8294.11(5779.28,10945.73) 8.76(6.11,11.56) 28574.06(22165.97,35672.60) 13.52(10.49,16.88) 244.51(158.93,363.61) 54.34(16.00,107.70) 1.47(1.26,1.67)

High-income Asia Pacific 28574.06(22165.97,35672.60) 11.33(10.62,12.06) 5888.21(5327.12,6576.97) 6.45(5.84,7.21) -39.39(-43.82,-34.32) -43.05(-47.21,-38.27) -2.06(-2.37,-1.74)

High-income North America 28088.98(26684.56,29921.18) 20.42(19.40,21.75) 32858.21(30334.15,35878.81) 18.06(16.67,19.72) 16.98(11.59,22.67) -11.57(-15.65,-7.27) -0.33(-0.50,-0.16)

North Africa and Middle East 12571.69(10038.69,15568.65) 7.24(5.78,8.96) 27387.15(23388.71,32354.86) 8.47(7.23,10.01) 117.85(65.05,187.55) 17.04(-11.32,54.49) 0.92(0.65,1.19)

Oceania 166.61(115.07,224.86) 4.91(3.39,6.63) 342.15(267.06,434.21) 4.76(3.71,6.04) 105.36(52.49,178.55) -3.12(-28.07,31.41) 0.07(-0.13,0.27)

South Asia 81325.24(66476.96,96213.36) 14.31(11.70,16.93) 144760.74(123483.12,166638.67) 15.38(13.12,17.71) 78.00(36.24,134.38) 7.51(-17.72,41.56) 0.23(0.07,0.39)

Southeast Asia 18197.09(16034.81,20998.74) 7.87(6.94,9.09) 38006.06(31287.87,47439.53) 10.87(8.95,13.57) 108.86(65.90,159.11) 38.11(9.70,71.34) 0.94(0.87,1.01)

Southern Latin America 13058.67(11366.34,14792.41) 53.84(46.86,60.99) 20274.93(17924.80,22673.82) 61.32(54.21,68.57) 55.26(33.95,81.79) 13.88(-1.75,33.34) 0.63(0.42,0.84)

Southern Sub-Saharan Africa 2640.91(2166.03,3088.89) 10.39(8.52,12.15) 5520.12(4769.15,6398.52) 14.08(12.17,16.32) 109.02(71.00,166.33) 35.55(10.90,72.71) 1.11(0.95,1.27)

Tropical Latin America 11753.66(11082.34,12462.16) 15.58(14.69,16.52) 29227.63(26846.60,31324.33) 26.29(24.15,28.18) 148.67(129.23,170.03) 68.72(55.54,83.22) 1.94(1.81,2.07)

Western Europe 56698.55(53748.43,59859.09) 30.29(28.71,31.97) 34189.11(30685.68,38494.40) 15.91(14.28,17.91) -39.70(-44.48,-34.33) -47.48(-51.64,-42.81) -1.70(-1.85,-1.55)

Western Sub-Saharan Africa 3947.79(3197.72,4702.77) 4.11(3.33,4.90) 9120.85(7178.70,11345.83) 3.82(3.00,4.75) 131.04(80.50,197.57) -7.22(-27.52,19.49) -0.52(-0.68,-0.36)


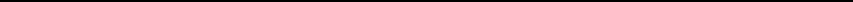
Abbreviations: EAPC, estimated annual percentage change; SDI, sociodemographic Index; UI, uncertainty interval.

^a^ EAPC is expressed as 95% confidence interval. ^b^ Change shows the percentage change.

**Supplementary Table S3: DALYs due to testicular cancer by country and territory, 1990–2021.**


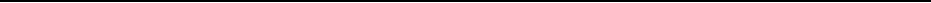
 **Rate per 100 000 (95%UI)**


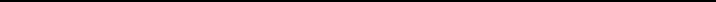

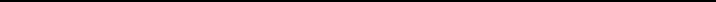
**1990 2021 1990-2021**

**location Number of DALYs DALYs rate Number of DALYs DALYs rate EAPC a**

| Afghanistan | 53.91(32.96,85.46) | 1.11(0.68,1.76) | 454.26(292.83,681.40) | 2.85(1.84,4.27) | 3.58(3.36,3.81) |
| --- | --- | --- | --- | --- | --- |
| Albania | 450.91(314.79,649.92) | 26.51(18.50,38.21) | 312.31(189.71,472.99) | 23.29(14.15,35.28) | 0.01(-0.21,0.23) |
| Algeria | 379.58(267.53,509.62) | 2.96(2.09,3.98) | 677.79(464.88,979.28) | 3.02(2.07,4.36) | 0.17(0.08,0.27) |
| American Samoa | 0.08(0.05,0.11) | 0.31(0.21,0.44) | 0.20(0.13,0.28) | 0.78(0.52,1.09) | 4.10(3.06,5.14) |
| Andorra | 6.39(4.13,9.36) | 22.12(14.32,32.41) | 6.00(3.83,9.22) | 13.63(8.69,20.95) | -1.32(-1.48,-1.17) |
| Angola | 418.48(274.94,592.50) | 8.08(5.31,11.44) | 1510.26(1015.60,2121.96) | 9.52(6.40,13.37) | 0.77(0.58,0.96) |
| Antigua and Barbuda | 0.66(0.59,0.73) | 2.25(2.03,2.51) | 4.84(4.22,5.53) | 11.06(9.63,12.63) | 5.23(3.88,6.59) |
| Argentina | 7836.36(6395.87,9515.90) | 48.36(39.47,58.73) | 12408.40(10723.40,14190.08) | 55.94(48.34,63.97) | 0.79(0.59,1.00) |
| Armenia | 191.21(135.04,257.36) | 11.41(8.06,15.36) | 271.22(196.46,362.20) | 18.80(13.62,25.11) | 1.57(1.20,1.95) |

| Australia | 2355.20(2149.75,2576.24) | 28.10(25.65,30.73) | 1996.32(1717.53,2365.62) | 15.66(13.48,18.56) | -1.99(-2.19,-1.78) |
| --- | --- | --- | --- | --- | --- |
| Austria | 1234.60(1107.18,1354.44) | 33.07(29.66,36.28) | 654.47(557.32,768.86) | 14.77(12.58,17.35) | -2.22(-2.41,-2.02) |
| Azerbaijan | 294.97(166.73,500.94) | 8.25(4.67,14.02) | 546.08(293.43,931.95) | 10.38(5.58,17.72) | 1.30(1.10,1.51) |
| Bahamas | 0.64(0.56,0.73) | 0.51(0.44,0.58) | 5.62(4.50,7.14) | 3.00(2.40,3.81) | 5.62(4.44,6.82) |
| Bahrain | 13.90(9.79,18.63) | 4.74(3.34,6.35) | 38.38(26.51,53.05) | 3.99(2.75,5.51) | 0.06(-0.66,0.78) |
| Bangladesh | 6594.14(3752.06,9527.94) | 11.76(6.69,16.99) | 10487.85(6593.86,15200.77) | 12.95(8.14,18.77) | 0.19(0.02,0.36) |
| Barbados | 2.11(1.92,2.33) | 1.73(1.57,1.92) | 6.99(5.33,8.87) | 4.85(3.70,6.16) | 2.70(1.06,4.37) |
| Belarus | 729.93(627.98,839.45) | 14.89(12.81,17.13) | 830.51(655.88,1032.76) | 19.13(15.11,23.79) | 0.40(0.11,0.69) |
| Belgium | 947.07(821.15,1075.32) | 19.37(16.80,22.00) | 636.24(534.66,770.63) | 11.26(9.47,13.64) | -1.68(-1.87,-1.48) |
| Belize | 1.05(0.95,1.16) | 1.11(1.00,1.23) | 23.53(20.54,26.89) | 11.04(9.64,12.62) | 6.92(5.26,8.60) |
| Benin | 150.53(113.64,198.95) | 6.37(4.81,8.42) | 300.76(212.10,408.99) | 4.53(3.19,6.16) | -1.64(-1.89,-1.39) |
| Bermuda | 0.39(0.33,0.45) | 1.35(1.15,1.56) | 1.56(1.25,2.00) | 5.07(4.06,6.51) | 4.11(3.15,5.08) |
| Bhutan | 33.23(16.49,52.88) | 9.97(4.95,15.86) | 50.72(29.71,80.73) | 12.92(7.57,20.57) | 1.04(0.89,1.18) |
| Bolivia (Plurinational State of) | 622.30(425.12,918.41) | 19.72(13.47,29.10) | 2203.77(1334.74,3418.19) | 37.37(22.63,57.97) | 2.18(1.94,2.43) |
| Bosnia and Herzegovina | 534.98(416.79,705.03) | 23.80(18.54,31.37) | 295.67(201.38,408.28) | 18.31(12.47,25.29) | -0.64(-0.94,-0.34) |
| Botswana | 67.72(41.63,100.33) | 10.72(6.59,15.89) | 185.02(113.25,277.85) | 15.65(9.58,23.50) | 1.08(0.96,1.20) |
| Brazil | 11472.66(10801.94,12181.97) | 15.63(14.72,16.60) | 28251.02(25918.19,30385.83) | 26.27(24.10,28.25) | 1.91(1.78,2.05) |
| Brunei Darussalam | 26.62(16.61,39.33) | 19.37(12.09,28.61) | 41.24(27.41,58.55) | 17.32(11.52,24.60) | -0.11(-0.28,0.06) |
| Bulgaria | 3093.36(2712.67,3541.10) | 72.28(63.39,82.74) | 2165.46(1749.31,2605.31) | 65.87(53.21,79.25) | 0.20(-0.14,0.55) |
| Burkina Faso | 246.28(153.28,341.90) | 5.34(3.33,7.42) | 453.30(296.46,637.87) | 4.12(2.69,5.79) | -1.20(-1.48,-0.92) |
| Burundi | 249.68(160.91,374.55) | 9.24(5.96,13.86) | 687.72(416.74,1126.31) | 10.39(6.29,17.01) | 0.56(0.31,0.81) |
| Cabo Verde | 2.29(1.66,3.04) | 1.36(0.99,1.81) | 5.39(3.44,8.51) | 1.91(1.22,3.02) | 1.19(1.13,1.25) |
| Cambodia | 283.32(199.96,416.13) | 5.81(4.10,8.53) | 858.21(566.07,1224.89) | 10.26(6.76,14.64) | 1.97(1.92,2.01) |
| Cameroon | 394.20(280.49,533.46) | 7.66(5.45,10.37) | 920.52(593.33,1361.04) | 5.80(3.74,8.58) | -1.33(-1.53,-1.14) |
| Canada | 2421.74(2154.86,2728.77) | 18.01(16.03,20.29) | 3187.55(2710.15,3775.47) | 17.24(14.66,20.42) | 0.08(-0.23,0.39) |
| Central African Republic | 101.01(63.75,157.23) | 7.52(4.74,11.70) | 219.43(134.96,341.84) | 8.16(5.02,12.71) | 0.23(0.14,0.33) |
| Chad | 162.79(98.86,237.67) | 5.56(3.38,8.12) | 378.79(239.96,569.89) | 4.29(2.72,6.46) | -1.15(-1.39,-0.90) |
| Chile | 4588.78(4054.96,5261.63) | 70.34(62.16,80.66) | 6974.71(5782.26,8198.40) | 75.40(62.51,88.62) | 0.27(-0.04,0.58) |
| China | 49458.82(40567.13,58497.16) | 8.15(6.68,9.64) | 44760.47(34566.40,56646.59) | 6.15(4.75,7.78) | -1.48(-1.78,-1.19) |
| Colombia | 3796.36(3379.01,4268.08) | 23.65(21.05,26.59) | 7500.28(6125.05,8942.38) | 31.19(25.47,37.19) | 1.53(1.19,1.87) |
| Comoros | 22.66(12.29,33.63) | 9.87(5.35,14.65) | 63.42(43.36,90.26) | 17.07(11.67,24.29) | 1.20(0.62,1.79) |
| Congo | 111.13(67.58,158.54) | 9.46(5.75,13.49) | 348.17(211.01,562.18) | 12.99(7.87,20.97) | 1.05(0.92,1.19) |
| Cook Islands | 0.24(0.15,0.36) | 2.45(1.55,3.61) | 0.29(0.19,0.41) | 3.40(2.24,4.77) | 1.19(1.11,1.28) |
| Costa Rica | 267.12(236.53,299.85) | 17.55(15.54,19.70) | 850.73(716.87,1000.69) | 37.11(31.27,43.65) | 2.53(2.29,2.77) |
| Côte d'Ivoire | 314.01(216.70,448.78) | 5.03(3.47,7.19) | 869.71(557.91,1234.26) | 6.04(3.88,8.58) | 0.37(0.16,0.58) |
| Croatia | 904.39(749.51,1072.57) | 38.25(31.70,45.37) | 520.57(419.89,645.04) | 25.49(20.56,31.59) | -0.85(-1.20,-0.51) |
| Cuba | 189.73(164.49,216.76) | 3.48(3.01,3.97) | 951.71(785.00,1146.26) | 17.00(14.02,20.48) | 4.47(3.31,5.63) |
| Cyprus | 68.22(51.52,90.13) | 17.43(13.17,23.03) | 95.53(72.11,126.56) | 14.27(10.77,18.90) | -0.85(-1.06,-0.64) |
| Czechia | 2784.11(2425.47,3224.95) | 55.76(48.57,64.59) | 1929.58(1514.93,2389.32) | 36.87(28.95,45.66) | -0.75(-1.05,-0.45) |
| Democratic People's Republic of Korea | 690.09(440.97,1015.37) | 7.13(4.55,10.49) | 850.90(541.70,1308.03) | 6.49(4.13,9.98) | -0.47(-0.61,-0.32) |
| Democratic Republic of the Congo | 1265.65(833.18,1783.11) | 6.71(4.42,9.46) | 3918.76(2362.88,5923.10) | 8.63(5.20,13.04) | 0.91(0.66,1.16) |
| Denmark | 823.40(728.46,928.33) | 32.49(28.74,36.63) | 420.19(350.15,508.79) | 14.41(12.01,17.45) | -2.70(-2.90,-2.49) |
| Djibouti | 30.12(19.37,43.44) | 13.80(8.87,19.90) | 137.04(83.76,213.24) | 20.20(12.34,31.43) | 1.19(1.02,1.37) |
| Dominica | 0.44(0.36,0.55) | 1.23(0.99,1.53) | 2.04(1.43,2.89) | 5.99(4.19,8.49) | 5.29(4.64,5.94) |
| Dominican Republic | 52.98(40.17,70.71) | 1.51(1.15,2.02) | 102.69(64.67,151.62) | 1.85(1.17,2.74) | 0.96(0.37,1.54) |
| Ecuador | 233.55(206.56,260.69) | 4.71(4.16,5.25) | 2522.93(2008.17,3147.26) | 28.09(22.36,35.05) | 5.39(3.27,7.55) |
| Egypt | 700.21(545.05,913.70) | 2.47(1.92,3.22) | 2134.69(1600.17,2895.82) | 3.90(2.93,5.30) | 1.72(1.48,1.96) |
| El Salvador | 322.71(265.82,389.52) | 12.46(10.26,15.04) | 674.96(508.93,872.90) | 22.33(16.83,28.87) | 2.29(2.14,2.44) |
| Equatorial Guinea | 15.11(9.59,23.82) | 7.42(4.71,11.70) | 102.37(59.52,169.89) | 12.47(7.25,20.69) | 1.94(1.79,2.10) |
| Eritrea | 162.04(110.88,228.51) | 9.52(6.51,13.43) | 532.28(324.35,796.85) | 15.88(9.68,23.78) | 1.70(1.60,1.80) |
| Estonia | 216.49(182.83,257.99) | 29.51(24.93,35.17) | 104.00(87.06,123.24) | 16.81(14.07,19.92) | -2.42(-2.65,-2.18) |
| Eswatini | 26.34(18.24,37.73) | 6.87(4.76,9.85) | 65.25(41.49,94.52) | 11.52(7.32,16.68) | 1.63(1.44,1.82) |
| Ethiopia | 2337.69(1037.03,4041.52) | 9.23(4.09,15.95) | 7132.86(3553.78,10216.32) | 12.99(6.47,18.61) | 1.12(0.92,1.32) |
| Federated States of Micronesia | 1.54(1.07,2.13) | 2.90(2.01,4.02) | 1.99(1.33,2.73) | 3.80(2.54,5.22) | 0.90(0.89,0.92) |
| Fiji | 78.90(57.60,111.60) | 20.49(14.96,28.99) | 125.77(91.18,169.39) | 26.86(19.47,36.18) | 1.28(0.89,1.67) |
| Finland | 422.68(377.29,472.45) | 17.39(15.53,19.44) | 246.60(206.40,293.20) | 9.02(7.55,10.72) | -1.58(-1.80,-1.35) |
| France | 8563.89(7714.11,9415.50) | 30.42(27.40,33.44) | 6050.05(5014.78,7385.70) | 18.80(15.58,22.95) | -1.08(-1.28,-0.89) |
| Gabon | 50.58(33.14,75.45) | 10.39(6.81,15.50) | 114.38(72.81,180.03) | 13.09(8.33,20.60) | 0.54(0.43,0.65) |
| Gambia | 44.19(31.89,60.21) | 9.01(6.50,12.27) | 132.17(86.01,204.86) | 11.21(7.30,17.38) | 0.20(-0.19,0.59) |
| Georgia | 1342.66(901.74,2092.37) | 51.27(34.43,79.89) | 1270.36(1005.74,1601.84) | 73.47(58.17,92.64) | 1.16(0.54,1.78) |
| Germany | 18137.84(16908.30,19485.70) | 46.99(43.80,50.48) | 9220.00(7910.18,10669.90) | 21.68(18.60,25.09) | -2.06(-2.35,-1.78) |
| Ghana | 530.90(339.57,826.76) | 7.14(4.57,11.13) | 1035.69(628.96,1722.17) | 6.21(3.77,10.33) | -0.88(-1.07,-0.68) |
| Greece | 1438.85(1298.01,1590.25) | 28.09(25.34,31.05) | 1077.22(952.62,1229.04) | 21.80(19.28,24.87) | -0.59(-0.82,-0.36) |
| Greenland | 20.84(15.14,28.58) | 69.35(50.36,95.09) | 6.22(4.23,8.91) | 21.11(14.35,30.24) | -3.50(-4.16,-2.84) |
| Grenada | 1.35(1.21,1.50) | 3.16(2.83,3.51) | 12.39(10.60,14.46) | 23.57(20.16,27.51) | 6.53(5.41,7.66) |
| Guam | 1.45(1.18,1.77) | 1.99(1.61,2.43) | 3.58(2.96,4.32) | 4.44(3.67,5.36) | 3.85(3.19,4.52) |
| Guatemala | 596.24(551.90,646.94) | 14.44(13.36,15.67) | 2538.09(2157.98,2932.15) | 33.23(28.25,38.38) | 3.27(2.85,3.69) |
| Guinea | 358.21(228.91,507.86) | 12.25(7.83,17.37) | 1036.54(658.89,1525.01) | 15.90(10.11,23.39) | 0.68(0.58,0.77) |
| Guinea-Bissau | 36.38(25.59,51.61) | 7.46(5.24,10.58) | 62.83(42.92,92.75) | 6.24(4.26,9.21) | -0.93(-1.10,-0.75) |
| Guyana | 8.14(6.68,9.85) | 2.11(1.73,2.55) | 58.33(44.33,74.75) | 15.46(11.75,19.82) | 6.30(4.90,7.72) |
| Haiti | 96.33(58.69,146.48) | 3.11(1.89,4.72) | 405.21(232.61,630.22) | 6.43(3.69,10.00) | 2.90(2.65,3.15) |
| Honduras | 175.66(122.98,254.35) | 7.51(5.25,10.87) | 479.97(263.04,781.84) | 9.77(5.36,15.92) | 0.80(0.71,0.88) |
| Hungary | 3377.32(3047.74,3740.75) | 67.62(61.02,74.89) | 2145.87(1741.18,2588.51) | 46.90(38.06,56.58) | -1.13(-1.39,-0.87) |
| Iceland | 16.24(14.13,18.66) | 12.74(11.09,14.64) | 12.54(10.39,15.51) | 7.06(5.85,8.74) | -1.28(-1.54,-1.02) |
| India | 60753.92(49943.09,72370.62) | 13.69(11.25,16.30) | 94225.88(80510.61,110088.36) | 13.01(11.12,15.20) | -0.14(-0.36,0.08) |
| Indonesia | 6719.77(5325.48,8499.97) | 7.28(5.77,9.21) | 15789.71(10295.30,23532.75) | 11.18(7.29,16.67) | 1.30(1.20,1.40) |
| Iran (Islamic Republic of) | 815.33(624.03,1062.37) | 2.80(2.14,3.65) | 4360.73(3651.83,5266.21) | 10.05(8.42,12.14) | 6.04(5.19,6.90) |
| Iraq | 420.14(272.43,616.04) | 4.43(2.87,6.50) | 1369.69(889.19,2031.50) | 6.41(4.16,9.51) | 1.31(1.25,1.36) |
| Ireland | 409.88(368.39,459.36) | 22.83(20.52,25.59) | 337.80(283.91,401.14) | 13.82(11.62,16.42) | -1.04(-1.29,-0.79) |
| Israel | 261.52(223.90,307.31) | 10.65(9.12,12.52) | 324.00(270.77,390.68) | 6.78(5.67,8.18) | -1.32(-1.71,-0.92) |
| Italy | 6507.56(6074.34,7018.96) | 23.61(22.03,25.46) | 5982.86(5274.19,6782.61) | 20.54(18.11,23.29) | 0.23(-0.07,0.53) |
| Jamaica | 13.91(11.94,16.31) | 1.20(1.03,1.40) | 101.96(75.31,135.07) | 7.34(5.42,9.72) | 4.85(3.42,6.30) |
| Japan | 8252.93(7766.79,8816.49) | 13.34(12.56,14.25) | 4917.04(4457.58,5470.89) | 7.90(7.16,8.79) | -1.89(-2.27,-1.52) |
| Jordan | 121.64(85.90,179.07) | 6.22(4.40,9.16) | 732.62(501.66,1065.86) | 11.10(7.60,16.15) | 2.44(2.05,2.82) |
| Kazakhstan | 1600.89(1162.78,2240.13) | 20.15(14.64,28.20) | 2023.00(1404.18,2761.65) | 22.01(15.28,30.04) | 0.10(-0.21,0.42) |
| Kenya | 182.92(108.80,253.90) | 1.59(0.95,2.21) | 664.10(494.81,864.45) | 2.67(1.99,3.48) | 1.74(1.66,1.82) |
| Kiribati | 1.16(0.80,1.70) | 3.15(2.16,4.60) | 2.42(1.64,3.48) | 4.08(2.77,5.87) | 1.01(0.95,1.08) |
| Kuwait | 41.35(33.70,50.09) | 4.21(3.43,5.10) | 128.49(96.05,171.31) | 5.23(3.91,6.97) | -0.47(-2.65,1.76) |
| Kyrgyzstan | 462.20(344.37,610.21) | 21.18(15.78,27.96) | 534.36(401.22,681.69) | 15.77(11.84,20.12) | -1.20(-1.65,-0.74) |
| Lao People's Democratic Republic | 118.15(77.81,189.85) | 5.77(3.80,9.27) | 301.25(198.20,449.82) | 8.12(5.34,12.12) | 1.26(1.15,1.36) |
| Latvia | 386.49(324.43,458.43) | 31.28(26.26,37.10) | 317.45(255.44,391.04) | 36.85(29.65,45.40) | -0.20(-0.52,0.12) |
| Lebanon | 75.51(50.69,111.51) | 5.09(3.42,7.52) | 334.14(233.20,465.89) | 12.07(8.43,16.83) | 3.36(3.10,3.62) |
| Lesotho | 44.93(30.07,60.34) | 6.45(4.32,8.66) | 111.73(77.42,155.14) | 12.28(8.51,17.06) | 2.30(2.20,2.40) |
| Liberia | 89.35(62.45,122.81) | 7.25(5.07,9.96) | 143.57(82.75,228.22) | 5.19(2.99,8.24) | -0.69(-0.91,-0.47) |
| Libya | 50.96(35.92,71.84) | 2.32(1.63,3.27) | 124.52(84.18,184.51) | 3.52(2.38,5.22) | 1.73(1.50,1.95) |
| Lithuania | 279.47(237.86,327.48) | 16.09(13.69,18.85) | 279.54(231.46,328.22) | 22.24(18.41,26.11) | 0.53(0.13,0.93) |
| Luxembourg | 67.69(61.29,74.66) | 36.25(32.82,39.98) | 36.84(32.24,42.25) | 11.36(9.94,13.03) | -3.54(-3.71,-3.37) |
| Madagascar | 514.58(387.43,677.49) | 8.67(6.52,11.41) | 1563.17(1068.75,2243.06) | 11.10(7.59,15.92) | 0.59(0.42,0.75) |
| Malawi | 805.50(556.20,1088.58) | 16.70(11.53,22.57) | 2461.37(1663.30,3581.69) | 25.98(17.56,37.81) | 1.41(1.34,1.48) |
| Malaysia | 1127.25(878.05,1453.52) | 12.64(9.85,16.30) | 2580.75(2045.09,3229.14) | 15.68(12.43,19.62) | 0.55(0.32,0.78) |
| Maldives | 3.89(2.65,5.68) | 3.42(2.33,4.99) | 18.16(12.66,25.67) | 5.70(3.97,8.06) | 2.42(2.00,2.85) |
| Mali | 436.41(326.73,555.46) | 10.24(7.67,13.03) | 1297.06(858.48,1841.34) | 10.82(7.16,15.36) | 0.16(-0.03,0.34) |
| Malta | 47.54(41.67,53.94) | 25.98(22.77,29.47) | 38.95(33.21,46.75) | 17.60(15.01,21.13) | -0.67(-0.93,-0.41) |
| Marshall Islands | 0.56(0.37,0.82) | 2.40(1.60,3.52) | 1.02(0.66,1.44) | 3.53(2.29,5.01) | 1.34(1.23,1.45) |
| Mauritania | 98.45(64.75,139.30) | 9.67(6.36,13.68) | 137.86(91.53,202.23) | 6.38(4.23,9.35) | -1.96(-2.16,-1.76) |
| Mauritius | 47.06(43.75,50.83) | 8.59(7.98,9.28) | 125.66(115.96,136.89) | 20.02(18.48,21.81) | 3.10(2.12,4.10) |
| Mexico | 15296.69(14725.43,15951.64) | 36.37(35.02,37.93) | 45581.21(40757.45,50926.68) | 72.25(64.60,80.72) | 2.48(2.24,2.72) |
| Monaco | 12.24(8.23,17.64) | 84.61(56.88,121.96) | 11.98(7.82,17.54) | 65.11(42.49,95.33) | -0.89(-0.98,-0.80) |
| Mongolia | 123.83(83.21,174.16) | 11.49(7.72,16.16) | 261.19(192.59,348.46) | 15.86(11.69,21.16) | 1.37(1.25,1.48) |
| Montenegro | 105.37(78.38,141.00) | 33.78(25.13,45.21) | 134.09(95.26,177.56) | 43.93(31.21,58.17) | 0.85(0.60,1.11) |
| Morocco | 154.11(105.27,214.63) | 1.22(0.83,1.70) | 240.44(157.86,388.04) | 1.28(0.84,2.07) | 0.28(0.15,0.41) |
| Mozambique | 549.46(350.83,780.42) | 8.59(5.49,12.21) | 2010.37(1194.28,3181.79) | 13.44(7.99,21.28) | 1.73(1.63,1.82) |
| Myanmar | 1188.46(842.95,1674.74) | 5.96(4.23,8.40) | 1901.42(1272.18,2684.39) | 6.98(4.67,9.86) | 0.42(0.35,0.48) |
| Namibia | 108.08(73.18,159.62) | 15.74(10.66,23.25) | 246.81(149.83,384.59) | 20.95(12.72,32.64) | 0.68(0.53,0.83) |
| Nauru | 0.16(0.12,0.23) | 3.07(2.18,4.26) | 0.18(0.13,0.25) | 3.26(2.28,4.57) | 0.18(0.14,0.22) |
| Nepal | 851.28(438.62,1319.40) | 8.72(4.49,13.52) | 1380.10(822.78,2047.63) | 9.32(5.56,13.83) | 0.20(-0.03,0.43) |
| Netherlands | 1789.31(1615.68,1996.97) | 24.26(21.91,27.08) | 1269.60(1085.97,1482.49) | 14.86(12.71,17.35) | -1.53(-1.66,-1.39) |
| New Zealand | 492.48(429.53,565.56) | 29.17(25.44,33.50) | 446.66(390.84,510.71) | 17.30(15.14,19.78) | -2.30(-2.77,-1.83) |
| Nicaragua | 188.47(128.33,269.54) | 9.88(6.73,14.13) | 658.58(407.66,1018.52) | 20.00(12.38,30.93) | 2.77(2.51,3.03) |
| Niger | 212.09(129.35,319.59) | 5.32(3.25,8.02) | 403.17(200.61,685.78) | 3.24(1.61,5.51) | -2.30(-2.62,-1.98) |
| Nigeria | 305.49(205.91,428.60) | 0.68(0.46,0.95) | 907.46(622.18,1236.30) | 0.82(0.56,1.12) | 0.78(0.60,0.97) |
| Niue | 0.04(0.03,0.05) | 3.19(2.32,4.50) | 0.03(0.02,0.04) | 3.79(2.81,5.17) | 0.57(0.42,0.71) |
| North Macedonia | 561.11(430.66,709.56) | 56.03(43.00,70.85) | 535.69(395.55,698.00) | 48.51(35.82,63.20) | -0.11(-0.29,0.06) |
| Northern Mariana Islands | 0.53(0.35,0.82) | 2.26(1.47,3.45) | 0.77(0.59,0.97) | 3.01(2.34,3.81) | 1.32(0.45,2.21) |
| Norway | 448.26(409.51,492.46) | 21.36(19.51,23.47) | 346.27(302.88,399.84) | 12.65(11.07,14.61) | -2.24(-2.98,-1.50) |
| Oman | 15.55(9.56,23.25) | 1.33(0.82,1.99) | 55.46(38.12,78.53) | 1.91(1.32,2.71) | 2.17(1.70,2.63) |
| Pakistan | 13092.67(9875.76,16599.11) | 22.48(16.95,28.50) | 38616.19(27092.06,53004.55) | 32.03(22.47,43.96) | 1.01(0.87,1.14) |
| Palau | 0.00(0.00,0.00) | 0.00(0.00,0.01) | 0.00(0.00,0.00) | 0.00(0.00,0.01) | -0.13(-0.28,0.02) |
| Palestine | 54.09(35.90,78.67) | 5.26(3.49,7.65) | 171.88(126.21,234.88) | 6.57(4.82,8.97) | 0.77(0.35,1.18) |
| Panama | 116.32(105.35,128.58) | 9.62(8.71,10.63) | 449.05(367.73,530.44) | 20.76(17.00,24.52) | 2.46(2.02,2.90) |
| Papua New Guinea | 40.96(17.12,74.56) | 1.92(0.80,3.49) | 139.58(83.58,212.87) | 2.57(1.54,3.92) | 0.93(0.78,1.07) |
| Paraguay | 281.00(214.83,361.88) | 13.82(10.56,17.79) | 976.61(685.21,1420.29) | 26.97(18.92,39.23) | 2.81(2.52,3.11) |
| Peru | 2970.58(2092.29,4050.98) | 27.60(19.44,37.64) | 4620.41(3193.40,6560.49) | 25.32(17.50,35.95) | -0.29(-0.51,-0.08) |
| Philippines | 2930.10(2488.71,3352.09) | 9.23(7.84,10.56) | 6168.67(4964.62,7586.67) | 10.74(8.65,13.21) | 0.43(0.30,0.56) |
| Poland | 9238.72(8848.51,9674.08) | 49.70(47.60,52.04) | 8570.57(7644.60,9355.12) | 46.31(41.31,50.55) | 0.03(-0.24,0.29) |
| Portugal | 1249.25(1112.46,1383.78) | 25.51(22.72,28.26) | 662.02(567.67,770.39) | 13.14(11.27,15.29) | -2.20(-2.41,-1.99) |
| Puerto Rico | 75.53(66.04,86.20) | 4.31(3.77,4.92) | 432.21(355.65,524.26) | 27.63(22.74,33.52) | 4.87(3.80,5.96) |
| Qatar | 2.84(1.97,3.94) | 0.96(0.66,1.33) | 60.56(40.11,87.57) | 2.88(1.91,4.17) | 6.49(5.19,7.81) |
| Republic of Korea | 1277.58(1019.32,1560.38) | 5.75(4.59,7.02) | 766.10(557.15,1035.09) | 2.96(2.15,4.00) | -2.54(-2.96,-2.11) |
| Republic of Moldova | 556.22(508.21,606.91) | 26.21(23.95,28.60) | 402.48(352.82,453.81) | 23.53(20.63,26.53) | -0.17(-0.45,0.11) |
| Romania | 4380.41(3951.22,4854.14) | 38.03(34.31,42.15) | 2795.85(2318.76,3348.02) | 30.37(25.18,36.36) | -0.36(-0.58,-0.14) |
| Russian Federation | 18038.08(17111.16,19045.67) | 25.50(24.19,26.93) | 15858.05(14438.98,17331.37) | 23.51(21.41,25.70) | -0.95(-1.40,-0.50) |
| Rwanda | 374.90(247.85,561.97) | 10.69(7.07,16.02) | 923.40(613.05,1353.53) | 14.30(9.49,20.96) | 1.06(0.81,1.31) |
| Saint Kitts and Nevis | 0.69(0.63,0.76) | 3.39(3.09,3.74) | 5.44(4.14,7.42) | 18.55(14.12,25.32) | 4.55(3.41,5.71) |
| Saint Lucia | 2.44(2.22,2.68) | 3.66(3.34,4.02) | 22.72(18.82,27.09) | 25.60(21.20,30.52) | 5.88(4.58,7.19) |
| Saint Vincent and the Grenadines | 1.12(1.01,1.25) | 2.05(1.85,2.29) | 9.12(7.99,10.40) | 15.66(13.71,17.85) | 5.75(4.48,7.04) |
| Samoa | 24.01(15.42,37.29) | 27.10(17.40,42.09) | 34.28(21.53,52.23) | 31.20(19.59,47.53) | 0.43(0.38,0.47) |
| San Marino | 1.56(1.07,2.10) | 13.29(9.11,17.98) | 1.21(0.71,1.86) | 7.65(4.45,11.73) | -1.14(-1.45,-0.84) |
| Sao Tome and Principe | 3.10(2.02,4.29) | 5.17(3.36,7.15) | 6.44(3.94,10.08) | 5.92(3.63,9.27) | 0.04(-0.24,0.33) |
| Saudi Arabia | 292.80(183.51,444.18) | 3.30(2.07,5.01) | 1355.85(843.79,2160.31) | 6.15(3.83,9.81) | 2.44(2.15,2.73) |
| Senegal | 305.72(218.96,410.91) | 8.21(5.88,11.04) | 561.68(381.76,843.66) | 7.05(4.79,10.59) | -0.78(-0.96,-0.60) |
| Serbia | 1897.57(1144.31,3105.56) | 40.02(24.14,65.50) | 1516.20(949.42,2308.81) | 34.07(21.33,51.88) | -0.41(-0.63,-0.19) |
| Seychelles | 5.08(3.79,6.83) | 13.97(10.42,18.78) | 7.50(5.53,9.54) | 13.49(9.96,17.18) | 0.34(0.14,0.54) |
| Sierra Leone | 125.94(83.47,174.15) | 6.17(4.09,8.54) | 220.36(134.19,316.76) | 5.01(3.05,7.20) | -0.89(-1.14,-0.63) |
| Singapore | 158.23(136.77,185.65) | 10.31(8.92,12.10) | 163.83(127.97,208.61) | 5.62(4.39,7.16) | -2.29(-2.83,-1.75) |
| Slovakia | 1222.42(841.52,1741.65) | 47.37(32.61,67.49) | 1120.46(718.45,1655.92) | 42.27(27.11,62.47) | -0.01(-0.13,0.11) |
| Slovenia | 327.84(290.76,365.66) | 34.22(30.35,38.16) | 217.04(177.41,268.30) | 21.11(17.26,26.10) | -1.64(-1.97,-1.31) |
| Solomon Islands | 3.67(1.77,6.26) | 2.09(1.00,3.56) | 10.83(6.71,16.21) | 3.11(1.93,4.65) | 1.31(1.22,1.39) |
| Somalia | 319.46(151.63,544.10) | 7.72(3.66,13.15) | 936.61(383.98,1702.00) | 8.48(3.47,15.40) | 0.47(0.42,0.53) |
| South Africa | 2129.67(1727.03,2487.01) | 11.86(9.62,13.85) | 4029.75(3476.11,4650.80) | 14.46(12.48,16.69) | 0.76(0.63,0.90) |
| South Sudan | 252.32(150.52,397.77) | 8.11(4.84,12.79) | 417.30(217.15,670.12) | 8.60(4.48,13.81) | 0.18(-0.03,0.39) |
| Spain | 3611.23(3252.97,3996.98) | 19.00(17.12,21.03) | 1962.65(1646.00,2343.97) | 8.82(7.39,10.53) | -2.37(-2.53,-2.20) |
| Sri Lanka | 613.22(471.33,802.48) | 7.12(5.47,9.32) | 762.93(515.91,1050.61) | 7.10(4.80,9.78) | -0.27(-0.44,-0.10) |
| Sudan | 156.43(104.55,235.04) | 1.55(1.03,2.33) | 910.85(582.19,1366.03) | 4.13(2.64,6.19) | 3.77(3.52,4.03) |
| Suriname | 8.54(5.97,11.12) | 4.39(3.07,5.72) | 39.62(27.72,55.63) | 13.86(9.70,19.46) | 3.37(2.43,4.32) |
| Sweden | 712.97(627.24,819.60) | 16.82(14.80,19.34) | 402.34(331.77,484.34) | 7.71(6.36,9.28) | -1.26(-1.79,-0.72) |
| Switzerland | 1018.02(904.28,1135.94) | 30.09(26.73,33.58) | 415.83(344.83,507.50) | 9.34(7.75,11.40) | -3.90(-4.07,-3.73) |
| Syrian Arab Republic | 148.56(102.05,213.08) | 2.29(1.57,3.28) | 188.76(132.32,280.15) | 2.80(1.96,4.15) | 0.73(0.54,0.93) |
| Taiwan (Province of China) | 853.83(762.71,946.35) | 8.10(7.24,8.98) | 934.20(815.63,1082.64) | 7.99(6.97,9.26) | -0.20(-0.64,0.24) |
| Tajikistan | 14.85(9.44,22.36) | 0.56(0.35,0.84) | 32.06(17.08,52.49) | 0.62(0.33,1.02) | 0.57(0.33,0.81) |
| Thailand | 2544.56(1871.28,3418.36) | 9.04(6.65,12.14) | 4579.47(3256.62,6012.69) | 14.23(10.12,18.68) | 1.25(1.07,1.44) |
| Timor-Leste | 22.56(14.34,33.22) | 5.59(3.55,8.23) | 46.45(31.75,68.01) | 6.57(4.49,9.62) | 0.38(-0.06,0.83) |
| Togo | 131.33(93.39,178.20) | 7.38(5.25,10.02) | 247.44(151.94,360.40) | 6.03(3.70,8.78) | -1.31(-1.58,-1.04) |
| Tokelau | 0.03(0.02,0.04) | 3.26(2.21,4.77) | 0.03(0.02,0.04) | 4.44(3.26,5.99) | 0.94(0.78,1.10) |
| Tonga | 1.09(0.77,1.45) | 2.18(1.55,2.92) | 1.65(1.16,2.40) | 3.11(2.19,4.54) | 1.10(0.94,1.27) |
| Trinidad and Tobago | 14.18(12.72,15.78) | 2.35(2.11,2.62) | 87.07(65.92,113.57) | 12.48(9.45,16.28) | 5.19(3.59,6.80) |
| Tunisia | 137.17(102.31,184.77) | 3.24(2.42,4.37) | 257.40(172.76,380.64) | 4.37(2.93,6.46) | 1.03(0.95,1.12) |
| Turkey | 8730.59(6378.75,11318.22) | 29.99(21.91,38.87) | 12648.82(9573.45,16387.68) | 30.16(22.83,39.08) | 0.23(-0.02,0.47) |
| Turkmenistan | 278.41(206.28,363.55) | 15.24(11.29,19.91) | 1200.02(771.30,1887.67) | 45.54(29.27,71.64) | 2.08(1.20,2.96) |
| Tuvalu | 0.12(0.08,0.17) | 2.57(1.76,3.65) | 0.22(0.15,0.31) | 3.35(2.34,4.76) | 0.89(0.85,0.93) |
| Uganda | 753.74(501.70,1069.51) | 8.85(5.89,12.56) | 2776.02(1831.04,4015.25) | 13.00(8.57,18.80) | 0.72(0.51,0.92) |
| Ukraine | 7059.29(5187.36,8763.62) | 28.92(21.25,35.91) | 6925.85(4593.84,9492.72) | 34.70(23.02,47.57) | 0.30(0.13,0.48) |
| United Arab Emirates | 126.82(82.85,195.05) | 10.48(6.85,16.12) | 610.25(417.81,827.82) | 8.79(6.02,11.92) | 0.24(-0.48,0.97) |
| United Kingdom | 8855.75(8433.88,9343.40) | 31.81(30.30,33.56) | 3947.82(3609.30,4393.04) | 11.85(10.84,13.19) | -2.83(-3.11,-2.54) |
| United Republic of Tanzania | 1353.27(948.89,1914.68) | 10.76(7.54,15.22) | 3896.78(2748.71,5306.34) | 13.71(9.67,18.67) | 0.88(0.77,0.99) |
| United States of America | 25645.75(24413.85,27254.81) | 20.67(19.68,21.96) | 29663.92(27512.14,32286.55) | 18.15(16.83,19.75) | -0.37(-0.53,-0.21) |
| United States Virgin Islands | 0.46(0.34,0.60) | 0.89(0.66,1.16) | 1.05(0.67,1.49) | 2.51(1.61,3.56) | 4.99(4.52,5.47) |
| Uruguay | 632.91(516.61,765.61) | 41.46(33.84,50.15) | 890.70(731.86,1066.86) | 54.57(44.84,65.36) | 0.83(0.65,1.02) |
| Uzbekistan | 891.39(613.88,1261.95) | 8.60(5.93,12.18) | 3206.49(2338.09,4396.65) | 18.77(13.68,25.73) | 2.54(2.16,2.92) |
| Vanuatu | 1.44(0.77,2.33) | 1.84(0.98,2.97) | 3.96(2.74,5.61) | 2.50(1.73,3.55) | 0.79(0.69,0.89) |
| Venezuela (Bolivarian Republic of) | 658.59(601.12,717.04) | 7.03(6.41,7.65) | 2485.80(1887.88,3247.34) | 19.30(14.66,25.21) | 3.91(3.06,4.77) |
| Viet Nam | 2567.35(1810.24,3515.41) | 7.75(5.46,10.61) | 4812.88(3314.37,6981.70) | 9.62(6.63,13.96) | 0.59(0.46,0.72) |
| Yemen | 73.33(37.27,124.87) | 1.06(0.54,1.80) | 506.02(297.24,795.70) | 2.98(1.75,4.69) | 4.14(3.68,4.61) |
| Zambia | 379.82(270.48,510.90) | 9.74(6.94,13.10) | 4346.74(2665.67,6725.14) | 44.98(27.58,69.59) | 6.44(5.36,7.53) |
| Zimbabwe | 264.17(198.03,346.06) | 5.21(3.91,6.83) | 881.54(578.29,1258.04) | 11.75(7.71,16.77) | 2.94(2.37,3.52) |

Abbreviations: EAPC, estimated annual percentage change; UI, uncertainty interval.

a EAPC is expressed as 95% confidence interval.

**Supplementary Table S4: Mortality from testicular cancer at the global and regional levels, 1990–2021.**

**Rate per 100 000(95%UI)**


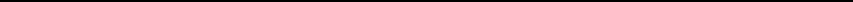

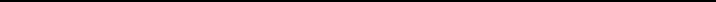

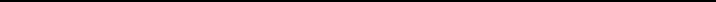

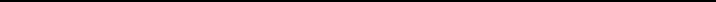


**1990 2021 1990-2021**

**Location** Number of deaths Mortality rate Number of deaths Mortality rate Cases change^b^  Rate change^b^ EAPC^a^

Global 7618.45(7191.60,8075.65) 0.28(0.27,0.30) 11388.30(10770.70,12055.62) 0.29(0.27,0.30) 49.48(36.41,63.63) 1.40(-7.47,10.99) 0.01(-0.13,0.15)

SDI

Low SDI 416.22(299.65,536.40) 0.16(0.12,0.21) 1010.75(811.92,1202.61) 0.18(0.15,0.22) 142.84(93.28,209.79) 9.60(-12.77,39.81) 0.22(0.04,0.39)

Low-middle SDI 1205.87(999.55,1422.64) 0.20(0.17,0.24) 2491.68(2207.51,2820.50) 0.26(0.23,0.29) 106.63(63.26,167.10) 26.51(-0.04,63.53) 0.82(0.71,0.94)

Middle SDI 1911.56(1786.98,2041.98) 0.22(0.20,0.23) 3799.87(3497.30,4103.53) 0.31(0.28,0.33) 98.78(79.15,119.03) 41.38(27.42,55.78) 1.07(0.93,1.21)

High-middle SDI 2137.13(2011.41,2272.80) 0.40(0.38,0.43) 2405.79(2230.21,2593.23) 0.37(0.34,0.40) 12.57(1.91,24.38) -8.70(-17.35,0.88) -0.48(-0.63,-0.34)

High SDI 1935.79(1899.32,1972.13) 0.45(0.44,0.46) 1667.74(1601.33,1738.47) 0.31(0.29,0.32) -13.85(-17.30,-10.48) -31.58(-34.32,-28.90) -1.11(-1.29,-0.92)

Regions

Andean Latin America 72.58(54.05,94.22) 0.38(0.29,0.50) 192.69(148.10,247.70) 0.58(0.45,0.75) 165.48(86.13,281.67) 51.32(6.09,117.54) 1.38(0.91,1.85)

Australasia 52.12(48.86,55.33) 0.52(0.49,0.55) 43.47(39.12,48.94) 0.28(0.26,0.32) -16.60(-24.99,-4.91) -45.20(-50.71,-37.52) -2.13(-2.34,-1.91)

Caribbean 10.14(9.16,11.40) 0.06(0.05,0.07) 58.87(50.58,69.25) 0.25(0.22,0.30) 480.27(380.10,595.93) 330.77(256.41,416.63) 4.30(3.36,5.24)

Central Asia 117.98(98.31,139.44) 0.35(0.29,0.41) 231.39(197.03,274.60) 0.49(0.41,0.58) 96.13(54.50,156.36) 40.01(10.30,83.01) 0.92(0.72,1.11)

Central Europe 601.45(569.19,642.02) 0.98(0.93,1.05) 510.08(466.99,555.05) 0.91(0.83,0.99) -15.19(-23.52,-6.62) -7.50(-16.58,1.86) -0.00(-0.17,0.17)

Central Latin America 389.23(374.34,402.85) 0.48(0.46,0.50) 1123.71(1018.42,1232.85) 0.91(0.83,1.00) 188.70(159.45,217.99) 90.03(70.77,109.30) 2.38(2.21,2.55)

Central Sub-Saharan Africa 39.21(27.27,51.98) 0.14(0.10,0.19) 122.48(84.84,168.41) 0.18(0.12,0.25) 212.36(112.24,362.14) 24.48(-15.42,84.16) 0.80(0.63,0.98)

East Asia 1077.76(888.65,1269.03) 0.17(0.14,0.20) 1285.67(1002.84,1621.15) 0.17(0.13,0.22) 19.29(-14.35,61.19) -0.65(-28.66,34.25) -0.64(-0.96,-0.32)

Eastern Europe 578.61(525.36,626.11) 0.55(0.50,0.59) 539.13(472.89,601.61) 0.56(0.49,0.63) -6.82(-19.70,9.13) 2.54(-11.63,20.10) -0.57(-0.92,-0.22)

Eastern Sub-Saharan Africa 154.37(107.67,202.00) 0.16(0.11,0.21) 516.81(403.07,643.95) 0.24(0.19,0.30) 234.77(151.42,347.39) 49.98(12.63,100.43) 1.38(1.16,1.60)

High-income Asia Pacific 171.80(164.72,178.63) 0.20(0.19,0.21) 123.93(116.44,130.36) 0.14(0.13,0.14) -27.87(-32.23,-23.51) -32.21(-36.31,-28.12) -1.49(-1.77,-1.22)

High-income North America 496.00(484.59,508.29) 0.36(0.35,0.37) 596.02(572.49,615.77) 0.33(0.31,0.34) 20.17(15.69,24.34) -9.16(-12.55,-6.01) -0.26(-0.42,-0.10)

North Africa and Middle East 227.57(183.41,283.23) 0.13(0.11,0.16) 466.68(403.12,539.13) 0.14(0.12,0.17) 105.07(58.03,167.39) 10.18(-15.10,43.66) 0.69(0.43,0.96)

Oceania 3.42(2.42,4.57) 0.10(0.07,0.13) 7.61(6.04,9.68) 0.11(0.08,0.13) 122.63(65.69,204.19) 5.02(-21.84,43.50) 0.39(0.16,0.61)

South Asia 1519.65(1244.61,1803.03) 0.27(0.22,0.32) 2752.85(2356.19,3175.64) 0.29(0.25,0.34) 81.15(38.57,137.02) 9.41(-16.31,43.15) 0.26(0.09,0.43)

Southeast Asia 358.80(316.68,412.21) 0.16(0.14,0.18) 818.26(676.98,1008.03) 0.23(0.19,0.29) 128.05(83.76,179.43) 50.80(21.51,84.78) 1.24(1.19,1.30)

Southern Latin America 249.65(220.17,280.43) 1.03(0.91,1.16) 384.61(344.09,424.12) 1.16(1.04,1.28) 54.06(35.57,75.71) 13.00(-0.56,28.89) 0.60(0.40,0.81)

Southern Sub-Saharan Africa 61.62(50.57,71.92) 0.24(0.20,0.28) 127.98(111.33,147.41) 0.33(0.28,0.38) 107.69(72.44,161.39) 34.69(11.83,69.51) 1.09(0.97,1.20)

Tropical Latin America 224.14(210.79,237.68) 0.30(0.28,0.32) 572.86(527.37,613.48) 0.52(0.47,0.55) 155.59(135.46,177.33) 73.42(59.76,88.17) 1.96(1.80,2.11)

Western Europe 1137.12(1103.20,1170.36) 0.61(0.59,0.63) 744.30(688.51,800.91) 0.35(0.32,0.37) -34.55(-38.59,-29.56) -42.99(-46.52,-38.65) -1.47(-1.69,-1.25)

Western Sub-Saharan Africa 75.21(61.02,89.28) 0.08(0.06,0.09) 168.90(133.93,211.29) 0.07(0.06,0.09) 124.57(75.43,190.83) -9.82(-29.56,16.79) -0.60(-0.76,-0.44)


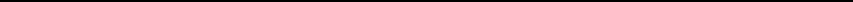


Abbreviations: EAPC, estimated annual percentage change; SDI, sociodemographic Index; UI, uncertainty interval.

^a^ EAPC is expressed as 95% confidence interval. ^b^ Change shows the percentage change.

**Supplementary Table S5: Mortality from testicular cancer by country and territory, 1990–2021.**

**Rate per 100 000 (95%UI)**


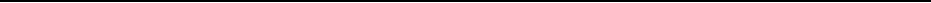

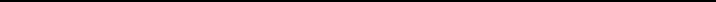

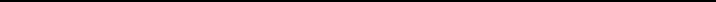


**1990 2021 1990-2021**

**location Number of deaths Mortality rate Number of deaths Mortality rate EAPC a**

| Afghanistan | 1.26(0.81,1.95) | 0.03(0.02,0.04) | 8.23(5.37,11.83) | 0.05(0.03,0.07) | 2.88(2.61,3.16) |
| --- | --- | --- | --- | --- | --- |
| Albania | 8.12(5.64,11.74) | 0.48(0.33,0.69) | 6.72(4.00,10.74) | 0.50(0.30,0.80) | 0.50(0.28,0.72) |
| Algeria | 6.43(4.68,8.51) | 0.05(0.04,0.07) | 11.08(8.00,15.47) | 0.05(0.04,0.07) | 0.09(-0.02,0.20) |
| American Samoa | 0.00(0.00,0.00) | 0.01(0.00,0.01) | 0.01(0.00,0.01) | 0.02(0.02,0.03) | 5.06(4.08,6.05) |
| Andorra | 0.12(0.07,0.18) | 0.40(0.26,0.61) | 0.11(0.07,0.17) | 0.26(0.17,0.39) | -1.23(-1.36,-1.10) |
| Angola | 8.34(5.50,11.85) | 0.16(0.11,0.23) | 29.52(20.38,41.33) | 0.19(0.13,0.26) | 0.70(0.53,0.88) |
| Antigua and Barbuda | 0.02(0.01,0.02) | 0.05(0.05,0.06) | 0.11(0.10,0.12) | 0.26(0.24,0.28) | 5.21(4.03,6.40) |
| Argentina | 155.20(129.60,183.78) | 0.96(0.80,1.13) | 238.80(210.06,268.98) | 1.08(0.95,1.21) | 0.68(0.50,0.86) |
| Armenia | 4.63(3.24,6.25) | 0.28(0.19,0.37) | 8.45(6.15,11.54) | 0.59(0.43,0.80) | 2.55(2.22,2.88) |

| Australia | 43.10(40.02,46.31) | 0.51(0.48,0.55) | | 35.03(31.47,39.56) | 0.27(0.25,0.31) | | -2.11(-2.33,-1.89) | |
| --- | --- | --- | --- | --- | --- | --- | --- | --- |
| Austria | 24.42(22.37,26.45) | 0.65(0.60,0.71) | | 14.09(12.22,16.01) | 0.32(0.28,0.36) | | -1.84(-2.04,-1.63) | |
| Azerbaijan | 6.73(3.88,11.49) | 0.19(0.11,0.32) | | 12.95(7.10,21.75) | 0.25(0.13,0.41) | | 1.38(1.18,1.57) | |
| Bahamas | 0.01(0.01,0.01) | 0.01(0.01,0.01) | | 0.11(0.09,0.14) | 0.06(0.05,0.08) | | 5.85(4.67,7.04) | |
| Bahrain | 0.24(0.17,0.31) | 0.08(0.06,0.11) | | 0.57(0.41,0.77) | 0.06(0.04,0.08) | | -0.59(-1.30,0.12) | |
| Bangladesh | 121.90(70.51,175.69) | 0.22(0.13,0.31) | | 198.98(125.91,288.26) | 0.25(0.16,0.36) | | 0.28(0.09,0.47) | |
| Barbados | 0.05(0.04,0.05) | 0.04(0.04,0.04) | | 0.19(0.15,0.24) | 0.13(0.10,0.17) | | 3.31(1.72,4.93) | |
| Belarus | 14.34(12.42,16.53) | 0.29(0.25,0.34) | | 19.98(15.85,25.23) | 0.46(0.37,0.58) | | 1.07(0.83,1.32) | |
| Belgium | 19.49(17.24,21.58) | 0.40(0.35,0.44) | | 13.55(11.72,15.62) | 0.24(0.21,0.28) | | -1.50(-1.74,-1.27) | |
| Belize | 0.02(0.02,0.02) | 0.02(0.02,0.02) | | 0.46(0.40,0.53) | 0.22(0.19,0.25) | | 6.97(5.32,8.64) | |
| Benin | 2.93(2.22,3.88) | 0.12(0.09,0.16) | | 5.63(4.07,7.62) | 0.08(0.06,0.11) | | -1.74(-1.98,-1.49) | |
| Bermuda | 0.01(0.01,0.01) | 0.03(0.03,0.03) | | 0.04(0.04,0.06) | 0.15(0.12,0.18) | | 4.96(4.05,5.89) | |
| Bhutan | 0.59(0.30,0.93) | 0.18(0.09,0.28) | | 0.95(0.56,1.50) | 0.24(0.14,0.38) | | 1.13(1.00,1.26) | |
| Bolivia (Plurinational State of) | 11.50(7.98,16.43) | 0.36(0.25,0.52) | | 42.49(25.97,63.92) | 0.72(0.44,1.08) | | 2.35(2.12,2.58) | |
| Bosnia and Herzegovina | 10.21(8.15,12.89) | 0.45(0.36,0.57) | | 6.97(5.03,9.32) | 0.43(0.31,0.58) | | -0.02(-0.24,0.21) | |
| Botswana | 1.41(0.92,2.02) | 0.22(0.15,0.32) | | 3.96(2.52,5.71) | 0.33(0.21,0.48) | | 1.17(1.06,1.28) | |
| Brazil | 218.60(205.52,232.06) | 0.30(0.28,0.32) | | 553.20(509.86,593.88) | 0.51(0.47,0.55) | | 1.92(1.76,2.09) | |
| Brunei Darussalam | 0.47(0.29,0.68) | 0.34(0.21,0.50) | | 0.73(0.48,1.02) | 0.31(0.20,0.43) | | -0.09(-0.25,0.07) | |
| Bulgaria | 64.98(57.87,73.42) | 1.52(1.35,1.72) | | 52.06(42.48,63.45) | 1.58(1.29,1.93) | | 0.63(0.32,0.94) | |
| Burkina Faso | 4.93(3.12,6.75) | 0.11(0.07,0.15) | | 8.80(5.85,12.19) | 0.08(0.05,0.11) | | -1.29(-1.57,-1.01) | |
| Burundi | 4.58(2.98,6.75) | 0.17(0.11,0.25) | | 12.49(7.62,19.99) | 0.19(0.12,0.30) | | 0.48(0.25,0.72) | |
| Cabo Verde | 0.08(0.06,0.11) | 0.05(0.03,0.06) | | 0.15(0.10,0.24) | 0.05(0.04,0.08) | | 0.53(0.40,0.66) | |
| Cambodia | 5.76(4.08,8.42) | 0.12(0.08,0.17) | | 18.31(12.31,25.95) | 0.22(0.15,0.31) | | 2.15(2.10,2.19) | |
| Cameroon | 7.48(5.36,10.18) | 0.15(0.10,0.20) | | 16.98(11.06,24.58) | 0.11(0.07,0.15) | | -1.41(-1.60,-1.23) | |
| Canada | 42.54(39.05,46.28) | 0.32(0.29,0.34) | | 52.33(47.23,58.16) | 0.28(0.26,0.31) | | -0.13(-0.43,0.17) | |
| Central African Republic | 2.00(1.29,3.12) | 0.15(0.10,0.23) | | 4.33(2.70,6.81) | 0.16(0.10,0.25) | | 0.24(0.14,0.35) | |
| Chad | 3.18(1.97,4.59) | 0.11(0.07,0.16) | | 7.08(4.54,10.51) | 0.08(0.05,0.12) | | -1.27(-1.50,-1.05) | |
| Chile | 81.58(72.39,92.68) | 1.25(1.11,1.42) | | 128.02(108.38,147.23) | 1.38(1.17,1.59) | | 0.40(0.07,0.73) | |
| China | 1047.51(858.23,1238.57) | 0.17(0.14,0.20) | | 1244.57(962.24,1579.64) | 0.17(0.13,0.22) | | -0.66(-0.98,-0.34) | |
| Colombia | 66.96(60.10,74.63) | 0.42(0.37,0.46) | | 140.19(115.67,167.17) | 0.58(0.48,0.70) | | 1.61(1.31,1.90) | |
| Comoros | 0.42(0.24,0.61) | 0.18(0.10,0.27) | | 1.21(0.82,1.73) | 0.32(0.22,0.47) | | 1.37(0.87,1.89) | |
| Congo | 2.23(1.40,3.16) | 0.19(0.12,0.27) | | 7.09(4.45,11.08) | 0.26(0.17,0.41) | | 1.12(1.02,1.23) | |
| Cook Islands | 0.01(0.00,0.01) | 0.06(0.04,0.08) | | 0.01(0.01,0.01) | 0.11(0.08,0.16) | | 2.36(2.23,2.48) | |
| Costa Rica | 5.04(4.50,5.63) | 0.33(0.30,0.37) | | 16.69(14.18,19.58) | 0.73(0.62,0.85) | | 2.69(2.44,2.94) | |
| Côte d'Ivoire | 5.70(3.98,8.02) | 0.09(0.06,0.13) | | 15.92(10.37,22.01) | 0.11(0.07,0.15) | | 0.40(0.20,0.60) | |
| Croatia | 18.26(15.38,21.42) | 0.77(0.65,0.91) | | 12.45(9.97,15.47) | 0.61(0.49,0.76) | | -0.33(-0.66,0.01) | |
| Cuba | 4.07(3.60,4.58) | 0.07(0.07,0.08) | | 27.35(22.52,32.46) | 0.49(0.40,0.58) | | 5.56(4.43,6.70) | |
| Cyprus | 1.48(1.13,1.90) | 0.38(0.29,0.49) | | 1.80(1.38,2.31) | 0.27(0.21,0.35) | | -1.54(-1.69,-1.38) | |
| Czechia | 57.59(50.66,65.53) | 1.15(1.01,1.31) | | 45.45(36.68,54.66) | 0.87(0.70,1.04) | | -0.41(-0.80,-0.02) | |
| Democratic People's Republic of Korea | 14.15(9.31,20.71) | 0.15(0.10,0.21) | | 19.84(12.74,30.25) | 0.15(0.10,0.23) | | -0.00(-0.13,0.13) | |
| Democratic Republic of the Congo | 25.25(16.60,35.09) | 0.13(0.09,0.19) | | 77.30(47.49,114.58) | 0.17(0.10,0.25) | | 0.85(0.61,1.08) | |
| Denmark | 17.51(15.95,19.22) | 0.69(0.63,0.76) | | 9.74(8.41,11.10) | 0.33(0.29,0.38) | | -2.54(-2.69,-2.38) | |
| Djibouti | 0.55(0.36,0.80) | 0.25(0.16,0.37) | | 2.57(1.59,4.00) | 0.38(0.23,0.59) | | 1.29(1.13,1.46) | |
| Dominica | 0.01(0.01,0.01) | 0.03(0.02,0.03) | | 0.05(0.03,0.06) | 0.14(0.10,0.19) | | 5.28(4.57,6.00) | |
| Dominican Republic | 1.01(0.79,1.31) | 0.03(0.02,0.04) | | 2.25(1.48,3.22) | 0.04(0.03,0.06) | | 1.28(0.66,1.91) | |
| Ecuador | 4.39(3.92,4.86) | 0.09(0.08,0.10) | | 52.06(41.36,64.59) | 0.58(0.46,0.72) | | 5.73(3.63,7.88) | |
| Egypt | 13.60(10.63,18.07) | 0.05(0.04,0.06) | | 40.31(30.37,54.43) | 0.07(0.06,0.10) | | 1.72(1.49,1.95) | |
| El Salvador | 5.68(4.75,6.76) | 0.22(0.18,0.26) | | 12.31(9.45,15.61) | 0.41(0.31,0.52) | | 2.37(2.25,2.49) | |
| Equatorial Guinea | 0.31(0.21,0.48) | 0.15(0.10,0.24) | | 1.86(1.12,3.00) | 0.23(0.14,0.37) | | 1.52(1.34,1.70) | |
| Eritrea | 2.93(2.03,4.10) | 0.17(0.12,0.24) | | 9.59(5.93,14.12) | 0.29(0.18,0.42) | | 1.69(1.62,1.77) | |
| Estonia | 4.25(3.60,4.95) | 0.58(0.49,0.68) | | 2.95(2.44,3.53) | 0.48(0.40,0.57) | | -1.41(-1.77,-1.06) | |
| Eswatini | 0.56(0.38,0.79) | 0.15(0.10,0.21) | | 1.31(0.84,1.85) | 0.23(0.15,0.33) | | 1.51(1.36,1.66) | |
| Ethiopia | 43.88(19.54,75.78) | | 0.17(0.08,0.30) | 130.55(65.03,186.13) | | 0.24(0.12,0.34) | | 1.05(0.85,1.26) |
| Federated States of Micronesia | 0.03(0.02,0.04) | 0.06(0.04,0.08) | | 0.04(0.03,0.06) | 0.08(0.06,0.11) | | 1.01(0.99,1.04) | |
| Fiji | 1.68(1.23,2.30) | 0.44(0.32,0.60) | | 3.04(2.18,4.13) | 0.65(0.46,0.88) | | 1.76(1.29,2.24) | |
| Finland | 8.40(7.55,9.33) | 0.35(0.31,0.38) | | 5.18(4.42,6.02) | 0.19(0.16,0.22) | | -1.55(-1.80,-1.31) | |
| France | 167.83(155.90,182.38) | 0.60(0.55,0.65) | | 117.95(100.51,137.01) | 0.37(0.31,0.43) | | -1.21(-1.42,-1.01) | |
| Gabon | 1.07(0.73,1.62) | 0.22(0.15,0.33) | | 2.37(1.58,3.64) | 0.27(0.18,0.42) | | 0.52(0.42,0.61) | |
| Gambia | 0.80(0.58,1.08) | 0.16(0.12,0.22) | | 2.35(1.55,3.61) | 0.20(0.13,0.31) | | 0.18(-0.18,0.55) | |
| Georgia | 32.71(22.00,50.68) | 1.25(0.84,1.94) | | 39.39(30.90,49.73) | 2.28(1.79,2.88) | | 2.05(1.46,2.65) | |
| Germany | 356.62(338.52,377.34) | 0.92(0.88,0.98) | | 206.53(182.23,234.37) | 0.49(0.43,0.55) | | -1.65(-2.01,-1.28) | |
| Ghana | 9.84(6.50,15.21) | 0.13(0.09,0.20) | | 19.17(11.77,31.47) | 0.12(0.07,0.19) | | -0.87(-1.06,-0.68) | |
| Greece | 29.22(27.27,31.51) | 0.57(0.53,0.62) | | 25.67(23.64,27.90) | 0.52(0.48,0.56) | | -0.04(-0.19,0.12) | |
| Greenland | 0.39(0.29,0.52) | 1.29(0.96,1.72) | | 0.13(0.09,0.18) | 0.45(0.31,0.62) | | -3.06(-3.66,-2.45) | |
| Grenada | 0.03(0.03,0.03) | 0.07(0.06,0.07) | | 0.27(0.23,0.31) | 0.52(0.44,0.60) | | 6.54(5.36,7.72) | |
| Guam | 0.03(0.02,0.03) | 0.04(0.03,0.05) | | 0.09(0.07,0.10) | 0.11(0.09,0.13) | | 4.78(4.11,5.45) | |
| Guatemala | 10.71(10.01,11.50) | 0.26(0.24,0.28) | | 45.82(39.28,52.73) | 0.60(0.51,0.69) | | 3.25(2.86,3.64) | |
| Guinea | 6.94(4.52,9.71) | 0.24(0.15,0.33) | | 19.02(12.23,27.82) | 0.29(0.19,0.43) | | 0.54(0.46,0.61) | |
| Guinea-Bissau | 0.68(0.48,0.94) | 0.14(0.10,0.19) | | 1.14(0.78,1.66) | 0.11(0.08,0.16) | | -1.00(-1.17,-0.84) | |
| Guyana | 0.16(0.13,0.19) | 0.04(0.03,0.05) | | 1.16(0.88,1.50) | 0.31(0.23,0.40) | | 6.46(5.08,7.86) | |
| Haiti | 1.94(1.23,2.90) | 0.06(0.04,0.09) | | 8.05(4.80,12.27) | 0.13(0.08,0.19) | | 2.80(2.58,3.03) | |
| Honduras | 3.16(2.26,4.49) | 0.14(0.10,0.19) | | 8.99(5.27,14.02) | 0.18(0.11,0.29) | | 0.96(0.89,1.03) | |
| Hungary | 68.73(62.36,75.57) | 1.38(1.25,1.51) | | 47.19(39.67,55.77) | 1.03(0.87,1.22) | | -0.86(-1.11,-0.61) | |
| Iceland | 0.29(0.26,0.32) | 0.23(0.20,0.25) | | 0.20(0.17,0.24) | 0.11(0.10,0.13) | | -1.51(-1.76,-1.25) | |
| India | 1136.31(934.20,1351.28) | 0.26(0.21,0.30) | | 1823.43(1562.03,2122.54) | 0.25(0.22,0.29) | | -0.06(-0.29,0.16) | |
| Indonesia | 133.44(105.43,169.31) | 0.14(0.11,0.18) | | 340.32(224.37,502.49) | 0.24(0.16,0.36) | | 1.56(1.46,1.66) | |
| Iran (Islamic Republic of) | 14.19(11.16,18.05) | 0.05(0.04,0.06) | | 75.65(65.95,86.88) | 0.17(0.15,0.20) | | 6.19(5.24,7.15) | |
| Iraq | 7.44(5.02,10.61) | 0.08(0.05,0.11) | | 21.89(14.81,31.65) | 0.10(0.07,0.15) | | 1.00(0.93,1.06) | |
| Ireland | 8.53(7.69,9.52) | 0.47(0.43,0.53) | | 6.45(5.61,7.36) | 0.26(0.23,0.30) | | -1.47(-1.64,-1.30) | |
| Israel | 5.33(4.71,6.07) | 0.22(0.19,0.25) | | 6.44(5.61,7.31) | 0.13(0.12,0.15) | | -1.43(-1.73,-1.13) | |
| Italy | 136.23(129.08,143.20) | 0.49(0.47,0.52) | | 139.41(125.07,153.11) | 0.48(0.43,0.53) | | 0.56(0.21,0.91) | |
| Jamaica | 0.33(0.28,0.37) | 0.03(0.02,0.03) | | 2.60(1.95,3.37) | 0.19(0.14,0.24) | | 5.55(4.18,6.93) | |
| Japan | 145.57(140.80,150.49) | 0.24(0.23,0.24) | | 107.17(101.27,111.66) | 0.17(0.16,0.18) | | -1.20(-1.51,-0.90) | |
| Jordan | 2.00(1.41,2.91) | 0.10(0.07,0.15) | | 10.39(7.19,14.44) | 0.16(0.11,0.22) | | 1.85(1.52,2.18) | |
| Kazakhstan | 35.42(25.14,50.24) | 0.45(0.32,0.63) | | 50.33(34.70,68.44) | 0.55(0.38,0.74) | | 0.39(0.11,0.67) | |
| Kenya | 3.29(1.96,4.58) | 0.03(0.02,0.04) | | 12.02(8.99,15.68) | 0.05(0.04,0.06) | | 1.77(1.68,1.85) | |
| Kiribati | 0.02(0.02,0.03) | 0.06(0.04,0.09) | | 0.05(0.03,0.07) | 0.08(0.06,0.12) | | 1.12(1.06,1.17) | |
| Kuwait | 0.58(0.49,0.66) | 0.06(0.05,0.07) | | 1.79(1.40,2.26) | 0.07(0.06,0.09) | | -0.61(-2.78,1.61) | |
| Kyrgyzstan | 8.70(6.52,11.39) | 0.40(0.30,0.52) | | 11.85(8.76,15.48) | 0.35(0.26,0.46) | | -0.80(-1.17,-0.42) | |
| Lao People's Democratic Republic | 2.42(1.63,3.81) | 0.12(0.08,0.19) | | 6.27(4.24,9.10) | 0.17(0.11,0.25) | | 1.28(1.18,1.39) | |
| Latvia | 7.83(6.54,9.41) | 0.63(0.53,0.76) | | 8.76(6.84,11.08) | 1.02(0.79,1.29) | | 1.00(0.69,1.32) | |
| Lebanon | 1.38(0.94,1.98) | | 0.09(0.06,0.13) | 5.40(3.86,7.35) | | 0.20(0.14,0.27) | | 2.93(2.74,3.12) |
| Lesotho | 1.08(0.72,1.45) | 0.15(0.10,0.21) | | 2.39(1.65,3.32) | 0.26(0.18,0.36) | | 1.88(1.82,1.95) | |
| Liberia | 1.72(1.23,2.34) | 0.14(0.10,0.19) | | 2.67(1.57,4.17) | 0.10(0.06,0.15) | | -1.10(-1.31,-0.89) | |
| Libya | 0.86(0.61,1.19) | 0.04(0.03,0.05) | | 2.03(1.39,2.84) | 0.06(0.04,0.08) | | 1.52(1.38,1.66) | |
| Lithuania | 5.41(4.61,6.31) | 0.31(0.27,0.36) | | 7.58(6.37,8.79) | 0.60(0.51,0.70) | | 1.70(1.24,2.15) | |
| Luxembourg | 1.33(1.23,1.45) | 0.71(0.66,0.77) | | 0.80(0.72,0.89) | 0.25(0.22,0.27) | | -3.20(-3.39,-3.00) | |
| Madagascar | 9.70(7.46,12.69) | 0.16(0.13,0.21) | | 28.02(19.50,39.16) | 0.20(0.14,0.28) | | 0.47(0.31,0.64) | |
| Malawi | 14.56(10.03,19.48) | 0.30(0.21,0.40) | | 43.53(29.38,63.05) | 0.46(0.31,0.67) | | 1.34(1.27,1.41) | |
| Malaysia | 22.64(18.07,28.60) | 0.25(0.20,0.32) | | 53.86(43.00,66.85) | 0.33(0.26,0.41) | | 0.72(0.49,0.95) | |
| Maldives | 0.09(0.06,0.13) | 0.08(0.05,0.11) | | 0.38(0.28,0.52) | 0.12(0.09,0.16) | | 1.98(1.65,2.30) | |
| Mali | 8.32(6.22,10.62) | 0.20(0.15,0.25) | | 23.75(15.69,33.85) | 0.20(0.13,0.28) | | 0.06(-0.12,0.24) | |
| Malta | 0.97(0.87,1.10) | 0.53(0.47,0.60) | | 0.84(0.72,0.97) | 0.38(0.32,0.44) | | -0.68(-0.85,-0.51) | |
| Marshall Islands | 0.01(0.01,0.02) | 0.05(0.03,0.07) | | 0.02(0.01,0.03) | 0.08(0.05,0.10) | | 1.57(1.50,1.64) | |
| Mauritania | 1.88(1.27,2.65) | 0.19(0.13,0.26) | | 2.65(1.75,3.86) | 0.12(0.08,0.18) | | -1.90(-2.09,-1.71) | |
| Mauritius | 1.02(0.96,1.10) | 0.19(0.17,0.20) | | 3.08(2.86,3.32) | 0.49(0.46,0.53) | | 3.53(2.54,4.54) | |
| Mexico | 279.94(270.45,290.96) | 0.67(0.64,0.69) | | 830.26(733.71,934.57) | 1.32(1.16,1.48) | | 2.46(2.24,2.69) | |
| Monaco | 0.27(0.18,0.38) | 1.84(1.26,2.66) | | 0.25(0.17,0.35) | 1.34(0.91,1.90) | | -1.10(-1.23,-0.96) | |
| Mongolia | 2.79(1.89,3.87) | 0.26(0.17,0.36) | | 6.01(4.41,7.88) | 0.36(0.27,0.48) | | 1.40(1.24,1.57) | |
| Montenegro | 2.00(1.52,2.60) | 0.64(0.49,0.83) | | 2.66(1.95,3.47) | 0.87(0.64,1.14) | | 0.94(0.72,1.17) | |
| Morocco | 2.82(1.95,3.95) | 0.02(0.02,0.03) | | 4.59(3.03,7.25) | 0.02(0.02,0.04) | | 0.42(0.30,0.54) | |
| Mozambique | 10.72(6.95,15.06) | 0.17(0.11,0.24) | | 36.45(22.19,56.48) | 0.24(0.15,0.38) | | 1.48(1.38,1.58) | |
| Myanmar | 24.55(17.58,34.24) | 0.12(0.09,0.17) | | 42.69(29.67,59.30) | 0.16(0.11,0.22) | | 0.74(0.69,0.79) | |
| Namibia | 1.97(1.33,2.84) | 0.29(0.19,0.41) | | 4.52(2.86,6.85) | 0.38(0.24,0.58) | | 0.72(0.58,0.86) | |
| Nauru | 0.00(0.00,0.00) | 0.06(0.05,0.09) | | 0.00(0.00,0.01) | 0.07(0.05,0.10) | | 0.18(0.12,0.23) | |
| Nepal | 15.83(8.17,24.17) | 0.16(0.08,0.25) | | 25.77(15.49,37.91) | 0.17(0.10,0.26) | | 0.23(-0.00,0.47) | |
| Netherlands | 36.91(33.72,40.69) | 0.50(0.46,0.55) | | 28.63(25.18,32.73) | 0.34(0.29,0.38) | | -1.33(-1.51,-1.14) | |
| New Zealand | 9.02(7.95,10.21) | 0.53(0.47,0.60) | | 8.44(7.51,9.47) | 0.33(0.29,0.37) | | -2.16(-2.63,-1.70) | |
| Nicaragua | 3.36(2.33,4.77) | 0.18(0.12,0.25) | | 12.32(7.88,18.41) | 0.37(0.24,0.56) | | 2.91(2.65,3.18) | |
| Niger | 4.03(2.46,5.96) | 0.10(0.06,0.15) | | 7.51(3.79,12.54) | 0.06(0.03,0.10) | | -2.30(-2.60,-2.00) | |
| Nigeria | 5.89(3.98,8.25) | 0.01(0.01,0.02) | | 16.62(11.64,22.58) | 0.02(0.01,0.02) | | 0.62(0.44,0.80) | |
| Niue | 0.00(0.00,0.00) | 0.09(0.07,0.12) | | 0.00(0.00,0.00) | 0.11(0.09,0.15) | | 1.10(0.89,1.31) | |
| North Macedonia | 11.55(9.04,14.52) | 1.15(0.90,1.45) | | 12.09(8.98,15.61) | 1.09(0.81,1.41) | | 0.11(-0.02,0.24) | |
| Northern Mariana Islands | 0.01(0.01,0.02) | 0.04(0.03,0.06) | | 0.02(0.02,0.03) | 0.08(0.07,0.10) | | 2.76(1.87,3.65) | |
| Norway | 9.18(8.56,9.80) | 0.44(0.41,0.47) | | 7.10(6.44,7.85) | 0.26(0.24,0.29) | | -2.29(-2.94,-1.65) | |
| Oman | 0.26(0.16,0.38) | 0.02(0.01,0.03) | | 0.72(0.52,0.97) | 0.02(0.02,0.03) | | 1.29(0.87,1.71) | |
| Pakistan | 245.01(184.29,309.81) | 0.42(0.32,0.53) | | 703.72(495.85,958.87) | 0.58(0.41,0.80) | | 0.91(0.78,1.04) | |
| Palau | 0.00(0.00,0.00) | 0.00(0.00,0.00) | | 0.00(0.00,0.00) | 0.00(0.00,0.00) | | 0.16(0.03,0.29) | |
| Palestine | 0.99(0.68,1.45) | 0.10(0.07,0.14) | | 2.74(2.12,3.61) | 0.10(0.08,0.14) | | 0.38(-0.02,0.77) | |
| Panama | 2.21(2.02,2.43) | 0.18(0.17,0.20) | | 9.04(7.34,10.73) | 0.42(0.34,0.50) | | 2.66(2.27,3.06) | |
| Papua New Guinea | 0.82(0.37,1.43) | 0.04(0.02,0.07) | | 2.96(1.80,4.41) | 0.05(0.03,0.08) | | 1.11(1.00,1.22) | |
| Paraguay | 5.53(4.32,7.07) | 0.27(0.21,0.35) | | 19.66(13.81,28.51) | 0.54(0.38,0.79) | | 2.85(2.58,3.12) | |
| Peru | 56.69(41.00,75.95) | 0.53(0.38,0.71) | | 98.15(67.59,138.01) | 0.54(0.37,0.76) | | 0.08(-0.13,0.29) | |
| Philippines | 59.28(50.48,67.81) | 0.19(0.16,0.21) | | 131.28(105.25,161.48) | 0.23(0.18,0.28) | | 0.61(0.51,0.71) | |
| Poland | 190.68(182.81,198.41) | 1.03(0.98,1.07) | | 187.59(167.83,205.98) | 1.01(0.91,1.11) | | 0.13(-0.17,0.43) | |
| Portugal | 25.35(22.85,28.00) | 0.52(0.47,0.57) | | 15.92(13.67,18.39) | 0.32(0.27,0.36) | | -1.61(-1.83,-1.39) | |
| Puerto Rico | 1.60(1.41,1.80) | 0.09(0.08,0.10) | | 10.53(8.70,12.80) | 0.67(0.56,0.82) | | 5.39(4.34,6.44) | |
| Qatar | 0.04(0.03,0.06) | 0.01(0.01,0.02) | | 0.72(0.49,0.99) | 0.03(0.02,0.05) | | 5.23(4.05,6.43) | |
| Republic of Korea | 22.94(18.38,27.86) | 0.10(0.08,0.13) | | 13.33(9.72,18.07) | 0.05(0.04,0.07) | | -2.75(-3.20,-2.29) | |
| Republic of Moldova | 12.19(11.20,13.28) | 0.57(0.53,0.63) | | 10.31(9.14,11.60) | 0.60(0.53,0.68) | | 0.25(-0.00,0.51) | |
| Romania | 88.34(80.81,97.07) | 0.77(0.70,0.84) | | 66.19(55.51,78.09) | 0.72(0.60,0.85) | | 0.08(-0.10,0.26) | |
| Russian Federation | 386.17(367.48,406.71) | 0.55(0.52,0.58) | | 339.72(307.90,369.31) | 0.50(0.46,0.55) | | -1.09(-1.58,-0.60) | |
| Rwanda | 6.78(4.58,10.10) | 0.19(0.13,0.29) | | 16.75(11.16,24.49) | 0.26(0.17,0.38) | | 1.03(0.77,1.28) | |
| Saint Kitts and Nevis | 0.02(0.01,0.02) | 0.08(0.07,0.09) | | 0.12(0.10,0.16) | 0.42(0.35,0.53) | | 4.72(3.63,5.83) | |
| Saint Lucia | 0.05(0.05,0.05) | 0.08(0.07,0.08) | | 0.52(0.43,0.61) | 0.58(0.48,0.69) | | 6.19(4.96,7.44) | |
| Saint Vincent and the Grenadines | 0.02(0.02,0.03) | 0.04(0.04,0.05) | | 0.22(0.20,0.25) | 0.38(0.34,0.43) | | 6.41(5.18,7.65) | |
| Samoa | 0.45(0.30,0.67) | 0.51(0.33,0.76) | | 0.67(0.43,0.98) | 0.61(0.39,0.89) | | 0.59(0.56,0.61) | |
| San Marino | 0.03(0.02,0.04) | 0.26(0.18,0.35) | | 0.03(0.02,0.04) | 0.17(0.10,0.25) | | -0.45(-0.84,-0.06) | |
| Sao Tome and Principe | 0.06(0.04,0.08) | 0.10(0.07,0.14) | | 0.12(0.07,0.19) | 0.11(0.07,0.17) | | -0.14(-0.37,0.10) | |
| Saudi Arabia | 5.26(3.34,7.85) | 0.06(0.04,0.09) | | 21.35(13.74,33.37) | 0.10(0.06,0.15) | | 1.91(1.67,2.15) | |
| Senegal | 5.81(4.23,7.73) | 0.16(0.11,0.21) | | 10.50(7.19,15.53) | 0.13(0.09,0.19) | | -0.81(-0.98,-0.64) | |
| Serbia | 40.23(25.52,64.29) | 0.85(0.54,1.36) | | 35.41(22.35,51.97) | 0.80(0.50,1.17) | | -0.03(-0.25,0.19) | |
| Seychelles | 0.10(0.08,0.13) | 0.27(0.21,0.37) | | 0.15(0.11,0.19) | 0.28(0.20,0.35) | | 0.42(0.26,0.57) | |
| Sierra Leone | 2.51(1.73,3.44) | 0.12(0.08,0.17) | | 4.14(2.55,5.88) | 0.09(0.06,0.13) | | -1.16(-1.42,-0.90) | |
| Singapore | 2.83(2.47,3.29) | 0.18(0.16,0.21) | | 2.70(2.25,3.27) | 0.09(0.08,0.11) | | -2.61(-3.13,-2.08) | |
| Slovakia | 24.72(17.22,34.36) | 0.96(0.67,1.33) | | 23.04(15.01,33.40) | 0.87(0.57,1.26) | | -0.04(-0.15,0.07) | |
| Slovenia | 6.42(5.76,7.10) | 0.67(0.60,0.74) | | 4.84(4.00,5.85) | 0.47(0.39,0.57) | | -1.28(-1.64,-0.93) | |
| Solomon Islands | 0.08(0.04,0.13) | 0.04(0.02,0.07) | | 0.23(0.15,0.34) | 0.07(0.04,0.10) | | 1.48(1.42,1.55) | |
| Somalia | 5.87(2.87,9.82) | 0.14(0.07,0.24) | | 16.71(7.03,30.06) | 0.15(0.06,0.27) | | 0.33(0.27,0.39) | |
| South Africa | 51.46(41.93,59.79) | 0.29(0.23,0.33) | | 99.68(86.28,114.76) | 0.36(0.31,0.41) | | 0.85(0.75,0.94) | |
| South Sudan | 4.87(2.94,7.69) | 0.16(0.09,0.25) | | 7.89(4.19,12.39) | 0.16(0.09,0.26) | | 0.13(-0.06,0.32) | |
| Spain | 71.06(65.36,77.25) | 0.37(0.34,0.41) | | 45.57(39.25,51.79) | 0.20(0.18,0.23) | | -1.81(-1.94,-1.69) | |
| Sri Lanka | 12.64(9.91,16.18) | 0.15(0.12,0.19) | | 17.31(11.51,24.54) | 0.16(0.11,0.23) | | 0.06(-0.10,0.22) | |
| Sudan | 3.06(2.10,4.42) | 0.03(0.02,0.04) | | 16.31(10.71,23.90) | 0.07(0.05,0.11) | | 3.49(3.24,3.75) | |
| Suriname | 0.17(0.12,0.21) | 0.09(0.06,0.11) | | 0.86(0.61,1.20) | 0.30(0.21,0.42) | | 3.77(2.83,4.70) | |
| Sweden | 14.76(13.22,16.59) | 0.35(0.31,0.39) | | 8.23(6.90,9.71) | 0.16(0.13,0.19) | | -1.43(-1.95,-0.92) | |
| Switzerland | 19.95(18.15,21.96) | 0.59(0.54,0.65) | | 9.48(7.96,11.37) | 0.21(0.18,0.26) | | -3.33(-3.48,-3.19) | |
| Syrian Arab Republic | 2.81(1.91,4.00) | 0.04(0.03,0.06) | | 3.81(2.63,5.51) | 0.06(0.04,0.08) | | 0.95(0.77,1.13) | |
| Taiwan (Province of China) | 16.10(14.63,17.58) | 0.15(0.14,0.17) | | 21.26(18.76,24.03) | 0.18(0.16,0.21) | | 0.30(-0.22,0.82) | |
| Tajikistan | 0.42(0.27,0.61) | 0.02(0.01,0.02) | | 0.72(0.39,1.18) | 0.01(0.01,0.02) | | -0.25(-0.44,-0.05) | |
| Thailand | 47.21(35.24,62.35) | 0.17(0.13,0.22) | | 105.19(76.68,137.49) | 0.33(0.24,0.43) | | 2.00(1.86,2.14) | |
| Timor-Leste | 0.43(0.28,0.63) | 0.11(0.07,0.16) | | 1.00(0.68,1.43) | 0.14(0.10,0.20) | | 0.85(0.53,1.16) | |
| Togo | 2.44(1.79,3.25) | 0.14(0.10,0.18) | | 4.69(2.86,6.81) | 0.11(0.07,0.17) | | -1.21(-1.46,-0.95) | |
| Tokelau | 0.00(0.00,0.00) | 0.09(0.06,0.12) | | 0.00(0.00,0.00) | 0.13(0.10,0.17) | | 1.53(1.22,1.85) | |
| Tonga | 0.03(0.02,0.03) | 0.05(0.04,0.07) | | 0.04(0.03,0.06) | 0.08(0.06,0.12) | | 1.55(1.41,1.70) | |
| Trinidad and Tobago | 0.29(0.27,0.32) | 0.05(0.04,0.05) | | 1.95(1.50,2.52) | 0.28(0.21,0.36) | | 5.30(3.78,6.85) | |
| Tunisia | 2.39(1.79,3.16) | 0.06(0.04,0.07) | | 4.19(2.79,5.99) | 0.07(0.05,0.10) | | 0.83(0.75,0.92) | |
| Turkey | 158.33(117.09,202.78) | 0.54(0.40,0.70) | | 214.76(165.16,271.67) | 0.51(0.39,0.65) | | -0.04(-0.28,0.20) | |
| Turkmenistan | 5.92(4.48,7.51) | 0.32(0.25,0.41) | | 27.25(17.96,42.33) | 1.03(0.68,1.61) | | 2.28(1.42,3.16) | |
| Tuvalu | 0.00(0.00,0.00) | 0.06(0.04,0.08) | | 0.01(0.00,0.01) | 0.08(0.06,0.11) | | 1.26(1.09,1.43) | |
| Uganda | 13.94(9.38,19.81) | 0.16(0.11,0.23) | | 48.90(32.95,70.84) | 0.23(0.15,0.33) | | 0.59(0.39,0.80) | |
| Ukraine | 148.42(108.82,182.87) | 0.61(0.45,0.75) | | 149.82(98.91,205.34) | 0.75(0.50,1.03) | | 0.43(0.26,0.59) | |
| United Arab Emirates | 2.05(1.36,3.12) | 0.17(0.11,0.26) | | 9.79(6.86,13.41) | 0.14(0.10,0.19) | | 0.12(-0.48,0.74) | |
| United Kingdom | 180.90(177.57,184.07) | 0.65(0.64,0.66) | | 79.69(76.49,82.23) | 0.24(0.23,0.25) | | -2.90(-3.23,-2.57) | |
| United Republic of Tanzania | 25.14(17.83,35.26) | 0.20(0.14,0.28) | | 70.80(50.34,96.96) | 0.25(0.18,0.34) | | 0.81(0.70,0.92) | |
| United States of America | 453.07(442.29,463.56) | 0.37(0.36,0.37) | | 543.56(522.87,562.93) | 0.33(0.32,0.34) | | -0.27(-0.43,-0.11) | |
| United States Virgin Islands | 0.01(0.01,0.01) | 0.02(0.01,0.02) | | 0.02(0.01,0.03) | 0.05(0.03,0.07) | | 4.54(4.15,4.93) | |
| Uruguay | 12.85(10.78,15.26) | 0.84(0.71,1.00) | | 17.76(14.85,20.79) | 1.09(0.91,1.27) | | 0.77(0.60,0.93) | |
| Uzbekistan | 20.67(13.71,28.98) | 0.20(0.13,0.28) | | 74.43(54.26,102.65) | 0.44(0.32,0.60) | | 2.58(2.18,2.98) | |
| Vanuatu | 0.03(0.02,0.05) | 0.04(0.02,0.06) | | 0.09(0.06,0.12) | 0.05(0.04,0.08) | | 1.00(0.91,1.09) | |
| Venezuela (Bolivarian Republic of) | 12.16(11.20,13.11) | 0.13(0.12,0.14) | | 48.10(36.73,61.67) | 0.37(0.29,0.48) | | 3.87(3.11,4.65) | |
| Viet Nam | 48.69(35.01,66.19) | 0.15(0.11,0.20) | | 97.27(68.18,137.08) | 0.19(0.14,0.27) | | 0.83(0.72,0.94) | |
| Yemen | 1.47(0.77,2.44) | 0.02(0.01,0.04) | | 9.93(6.06,15.17) | 0.06(0.04,0.09) | | 4.05(3.64,4.46) | |
| Zambia | 7.04(5.10,9.33) | 0.18(0.13,0.24) | | 78.88(48.75,120.16) | 0.82(0.50,1.24) | | 6.37(5.29,7.46) | |
| Zimbabwe | 5.15(3.93,6.67) | 0.10(0.08,0.13) | | 16.12(10.80,22.74) | 0.21(0.14,0.30) | | 2.64(2.17,3.12) | |

Abbreviations: EAPC, estimated annual percentage change; UI, uncertainty interval.

a EAPC is expressed as 95% confidence interval.

**Supplementary Table S6: Decomposition analysis of testicular cancer incidence rates globally, across five SDI regions and 21 GBD regions.**

| **Location** | **Overll difference** | **Aging** | **Population** | **Epidemiological change** | **Percent change**  **of aging** | **Percent change of population** | **Percent change of**  **epidemiological change** |
| --- | --- | --- | --- | --- | --- | --- | --- |
| Andean Latin America | 820.2 | 146.31 | 177.69 | 496.2 | 17.84 | 21.66 | 60.5 |
| Australasia | 522.35 | -36.26 | 378.82 | 179.79 | -6.94 | 72.52 | 34.42 |
| Caribbean | 282.86 | 0.4 | 58.41 | 224.05 | 0.14 | 20.65 | 79.21 |
| Central Asia | 501.79 | 53.17 | 200.69 | 247.93 | 10.6 | 40 | 49.41 |
| Central Europe | 2503.4 | -1988.99 | 1529.62 | 2962.77 | -79.45 | 61.1 | 118.35 |
| Central Latin America | 5384.71 | 330.85 | 1222.52 | 3831.34 | 6.14 | 22.7 | 71.15 |
| Central Sub-Saharan Africa | 171.34 | 68.99 | 46.56 | 55.79 | 40.26 | 27.18 | 32.56 |
| East Asia | 5093.37 | -292.69 | 1651.32 | 3734.75 | -5.75 | 32.42 | 73.33 |
| Eastern Europe | 2165.03 | -1664.68 | 1363.78 | 2465.92 | -76.89 | 62.99 | 113.9 |
| Eastern Sub-Saharan Africa | 848.2 | 287.86 | 212.09 | 348.25 | 33.94 | 25.01 | 41.06 |
| Global | 52673.89 | 3617.91 | 23880.83 | 25175.15 | 6.87 | 45.34 | 47.79 |
| High SDI | 11970.2 | -6276.52 | 10884.88 | 7361.84 | -52.43 | 90.93 | 61.5 |
| High-income Asia Pacific | 469.73 | -1251.59 | 1044.25 | 677.06 | -266.45 | 222.31 | 144.14 |
| High-income North America | 5933.06 | -2366.38 | 4102.88 | 4196.55 | -39.88 | 69.15 | 70.73 |
| High-middle SDI | 16954.47 | -3299.81 | 6801.75 | 13452.54 | -19.46 | 40.12 | 79.35 |
| Low SDI | 1590.31 | 608.79 | 483 | 498.52 | 38.28 | 30.37 | 31.35 |
| Low-middle SDI | 5530.01 | 1112.34 | 1640.36 | 2777.32 | 20.11 | 29.66 | 50.22 |
| Middle SDI | 16567.61 | -19.37 | 4380.49 | 12206.49 | -0.12 | 26.44 | 73.68 |
| North Africa and Middle East | 10299.26 | 2207.51 | 2194.27 | 5897.48 | 21.43 | 21.31 | 57.26 |
| Oceania | 9.57 | 4.84 | 4.17 | 0.55 | 50.6 | 43.62 | 5.78 |
| South Asia | 5873.26 | 1398.78 | 1887.69 | 2586.79 | 23.82 | 32.14 | 44.04 |
| Southeast Asia | 2398.32 | 351.23 | 682.8 | 1364.29 | 14.64 | 28.47 | 56.89 |
| Southern Latin America | 2707.88 | -1.1 | 778.26 | 1930.72 | -0.04 | 28.74 | 71.3 |
| Southern Sub-Saharan Africa | 198.51 | 48.51 | 74.56 | 75.44 | 24.44 | 37.56 | 38 |
| Tropical Latin America | 2361.61 | 114.14 | 595.11 | 1652.36 | 4.83 | 25.2 | 69.97 |
| Western Europe | 3886.08 | -5213.64 | 5736.51 | 3363.21 | -134.16 | 147.62 | 86.55 |
| Western Sub-Saharan Africa | 243.37 | 111.8 | 82.61 | 48.96 | 45.94 | 33.94 | 20.12 |

**Supplementary Table S7: Decomposition analysis of testicular cancer mortality rates globally, across five SDI regions and 21 GBD regions.**

| **Location** | **Overll difference** | **Aging** | **Population** | **Epidemiological change** | **Percent change**  **of aging** | **Percent change of population** | **Percent change of**  **epidemiological change** |
| --- | --- | --- | --- | --- | --- | --- | --- |
| Andean Latin America | 120.11 | 37.49 | 59.39 | 23.23 | 31.21 | 49.45 | 19.34 |
| Australasia | -8.65 | 2.64 | 24.97 | -36.26 | -30.49 | -288.6 | 419.09 |
| Caribbean | 48.72 | 1.39 | 14.37 | 32.96 | 2.85 | 29.5 | 67.64 |
| Central Asia | 113.41 | 9.76 | 81.73 | 21.92 | 8.6 | 72.07 | 19.33 |
| Central Europe | -91.37 | -228.16 | 282.13 | -145.34 | 249.72 | -308.79 | 159.07 |
| Central Latin America | 734.48 | 75.86 | 340.32 | 318.31 | 10.33 | 46.33 | 43.34 |
| Central Sub-Saharan Africa | 83.27 | 34.82 | 35.56 | 12.89 | 41.82 | 42.71 | 15.48 |
| East Asia | 207.91 | 109.74 | 585.77 | -487.59 | 52.78 | 281.74 | -234.52 |
| Eastern Europe | -39.48 | -249.04 | 284.53 | -74.97 | 630.76 | -720.65 | 189.89 |
| Eastern Sub-Saharan Africa | 362.43 | 128.89 | 145.5 | 88.04 | 35.56 | 40.14 | 24.29 |
| Global | 3769.86 | 562.01 | 4621.55 | -1413.7 | 14.91 | 122.59 | -37.5 |
| High SDI | -268.05 | -204.16 | 921.02 | -984.91 | 76.16 | -343.6 | 367.44 |
| High-income Asia Pacific | -47.87 | -29.39 | 75.85 | -94.33 | 61.4 | -158.43 | 197.03 |
| High-income North America | 100.02 | -93.83 | 269.98 | -76.13 | -93.81 | 269.93 | -76.12 |
| High-middle SDI | 268.66 | -130.31 | 1136.58 | -737.6 | -48.5 | 423.05 | -274.55 |
| Low SDI | 594.54 | 261.53 | 326.18 | 6.82 | 43.99 | 54.86 | 1.15 |
| Low-middle SDI | 1285.81 | 336 | 860.55 | 89.27 | 26.13 | 66.93 | 6.94 |
| Middle SDI | 1888.31 | 157.2 | 1346.82 | 384.29 | 8.32 | 71.32 | 20.35 |
| North Africa and Middle East | 239.1 | 130.68 | 163.03 | -54.61 | 54.65 | 68.19 | -22.84 |
| Oceania | 4.19 | 2.09 | 2.56 | -0.46 | 49.95 | 61.07 | -11.03 |
| South Asia | 1233.2 | 444.39 | 1014.69 | -225.88 | 36.04 | 82.28 | -18.32 |
| Southeast Asia | 459.46 | 106.71 | 269.65 | 83.1 | 23.23 | 58.69 | 18.09 |
| Southern Latin America | 134.96 | -12.25 | 152.85 | -5.64 | -9.07 | 113.25 | -4.18 |
| Southern Sub-Saharan Africa | 66.36 | 15.34 | 44.05 | 6.98 | 23.11 | 66.38 | 10.51 |
| Tropical Latin America | 348.73 | 38.28 | 182.08 | 128.37 | 10.98 | 52.21 | 36.81 |
| Western Europe | -392.82 | -188.24 | 487.29 | -691.86 | 47.92 | -124.05 | 176.13 |
| Western Sub-Saharan Africa | 93.69 | 48.71 | 56.72 | -11.74 | 51.99 | 60.54 | -12.53 |

**Supplementary Table S8: Decomposition analysis of testicular cancer DALYs rates globally, across five SDI regions and 21 GBD regions.**

| **Location** | **Overll difference** | **Aging** | **Population** | **Epidemiological change** | **Percent change**  **of aging** | **Percent change of population** | **Percent change of**  **epidemiological change** |
| --- | --- | --- | --- | --- | --- | --- | --- |
| Andean Latin America | 5520.67 | 1960.94 | 2385.05 | 1174.68 | 35.52 | 43.2 | 21.28 |
| Australasia | -404.69 | 9.28 | 1069.06 | -1483.03 | -2.29 | -264.17 | 366.46 |
| Caribbean | 1866.58 | 17.53 | 490.74 | 1358.3 | 0.94 | 26.29 | 72.77 |
| Central Asia | 4144.39 | 754.34 | 2729.82 | 660.22 | 18.2 | 65.87 | 15.93 |
| Central Europe | -6759.71 | -11789.47 | 10410.12 | -5380.35 | 174.41 | -154 | 79.59 |
| Central Latin America | 39800.52 | 4354.43 | 14857.87 | 20588.21 | 10.94 | 37.33 | 51.73 |
| Central Sub-Saharan Africa | 4251.42 | 2158.21 | 1441.53 | 651.68 | 50.76 | 33.91 | 15.33 |
| East Asia | -4457.17 | -4612.11 | 19374.01 | -19219.07 | 103.48 | -434.67 | 431.19 |
| Eastern Europe | -2548.07 | -11861.73 | 10394.33 | -1080.67 | 465.52 | -407.93 | 42.41 |
| Eastern Sub-Saharan Africa | 20279.96 | 8730.34 | 6391.5 | 5158.11 | 43.05 | 31.52 | 25.43 |
| Global | 171104.5 | 27824.67 | 183278.49 | -39998.66 | 16.26 | 107.11 | -23.38 |
| High SDI | -18458.09 | -16699.01 | 36751.66 | -38510.74 | 90.47 | -199.11 | 208.64 |
| High-income Asia Pacific | -3827.14 | -3039.81 | 3137.56 | -3924.89 | 79.43 | -81.98 | 102.55 |
| High-income North America | 4769.23 | -5832.47 | 11921.31 | -1319.61 | -122.29 | 249.96 | -27.67 |
| High-middle SDI | 5538.37 | -14218.03 | 42186.92 | -22430.52 | -256.72 | 761.72 | -405 |
| Low SDI | 32650.87 | 17769.04 | 13997.12 | 884.71 | 54.42 | 42.87 | 2.71 |
| Low-middle SDI | 66320.27 | 23892.88 | 36010.45 | 6416.95 | 36.03 | 54.3 | 9.68 |
| Middle SDI | 85077.58 | 1740.19 | 53251.44 | 30085.95 | 2.05 | 62.59 | 35.36 |
| North Africa and Middle East | 14815.45 | 7903.35 | 7396.94 | -484.83 | 53.35 | 49.93 | -3.27 |
| Oceania | 175.54 | 107.27 | 95.22 | -26.95 | 61.11 | 54.25 | -15.35 |
| South Asia | 63435.5 | 30764.79 | 42771.37 | -10100.66 | 48.5 | 67.42 | -15.92 |
| Southeast Asia | 19808.96 | 5217.91 | 10379.27 | 4211.79 | 26.34 | 52.4 | 21.26 |
| Southern Latin America | 7216.26 | 131.69 | 6357.43 | 727.14 | 1.82 | 88.1 | 10.08 |
| Southern Sub-Saharan Africa | 2879.21 | 951.91 | 1508.5 | 418.81 | 33.06 | 52.39 | 14.55 |
| Tropical Latin America | 17473.97 | 1734.91 | 7506.5 | 8232.57 | 9.93 | 42.96 | 47.11 |
| Western Europe | -22509.44 | -13557.35 | 18349.96 | -27302.05 | 60.23 | -81.52 | 121.29 |
| Western Sub-Saharan Africa | 5173.05 | 3340.24 | 2423.88 | -591.07 | 64.57 | 46.86 | -11.43 |

**Supplementary Figure S1: Testicular cancer mortality in 204 countries and territories in 2021.** A, Number of death cases. B, Mortality rate. C, Estimated annual percentage change (EAPC) in mortality rate from 1990 to 2021.

**
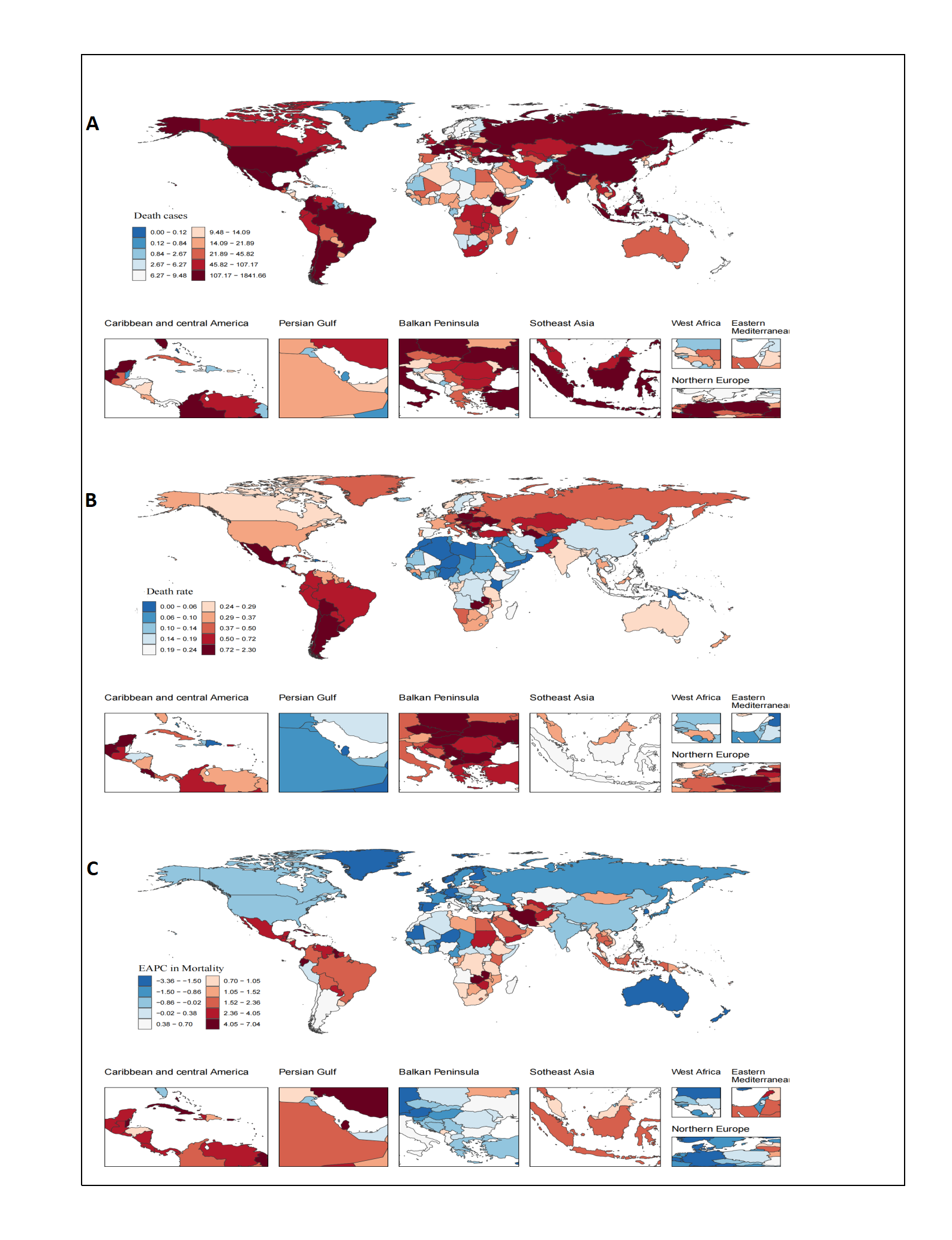
**

**Supplementary Figure S2: Testicular cancer DALYs in 204 countries and territories in 2021.** A, Number of DALYs cases. B, **DALYs rate**. C, Estimated annual percentage change (EAPC) in DALYs rate from 1990 to 2021.


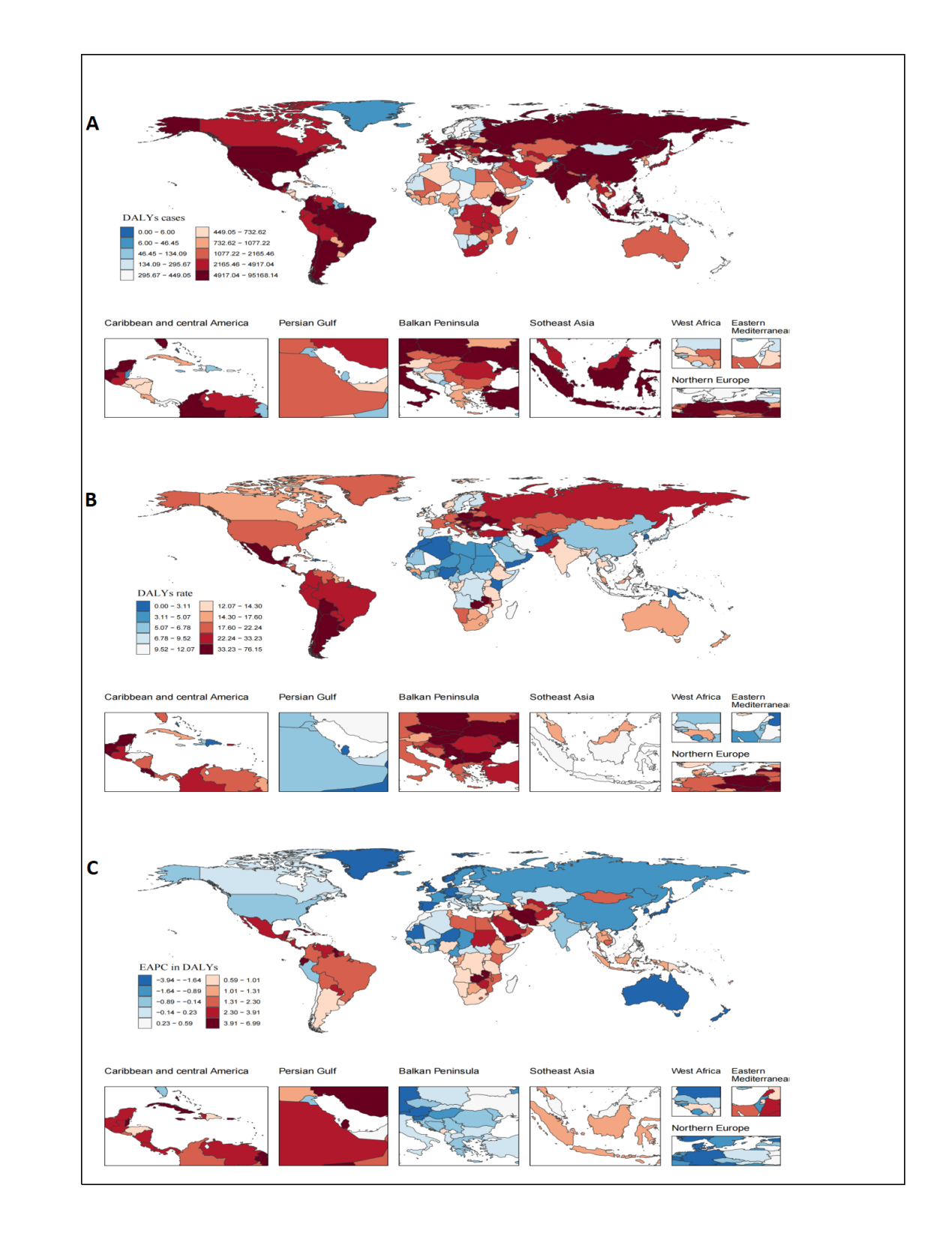


**Supplementary Figure S3: Age-specific proportions of testicular cancer incidence, mortality, and DALYs rates in 1990.** A, Incidence rate. B, Mortality rate. C, DALYs rate.


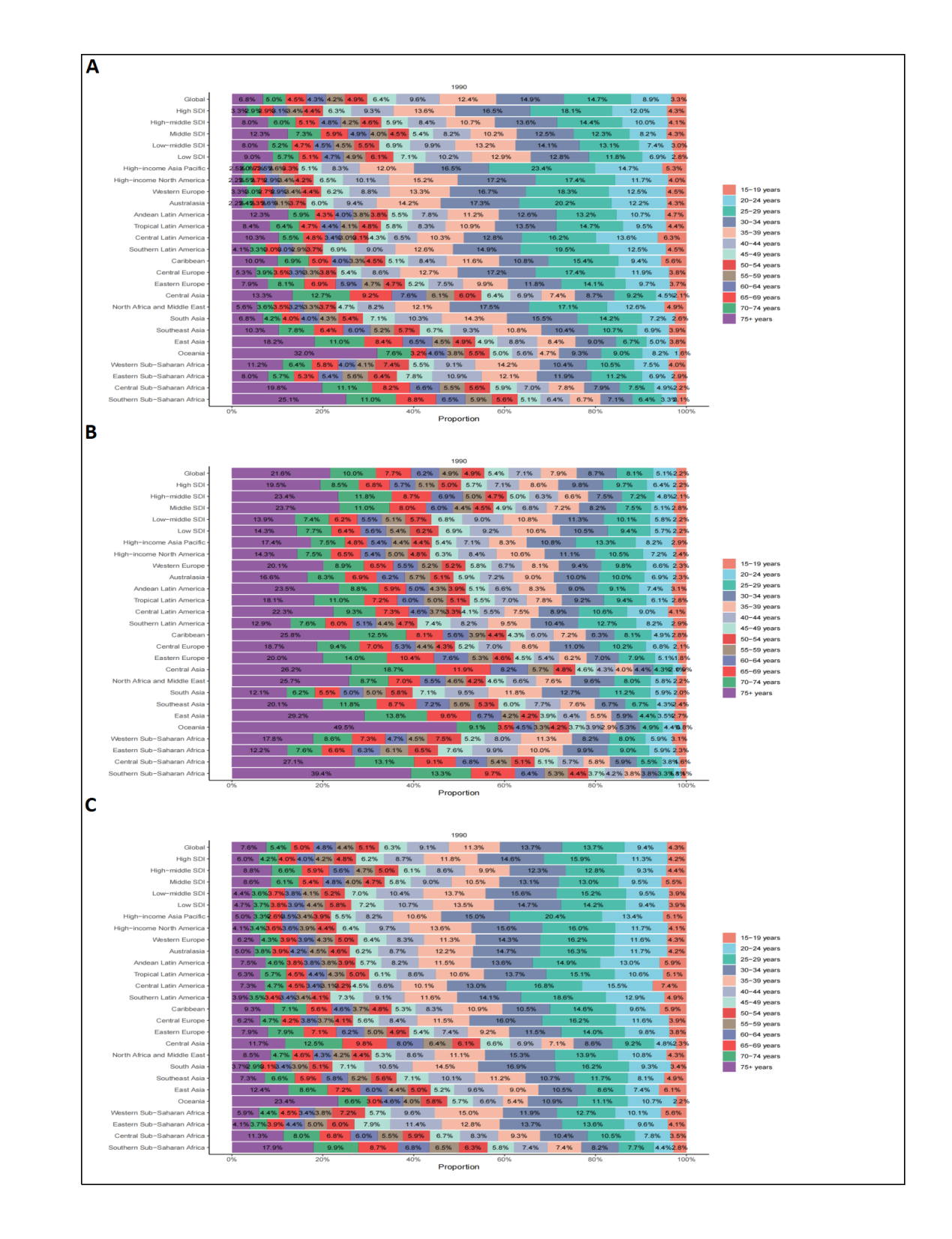


**Supplementary Figure S4: Association between EAPC, disease burden, and SDI in 2021.** A, Incidence rate. B, Mortality rate. C, DALYs rate.


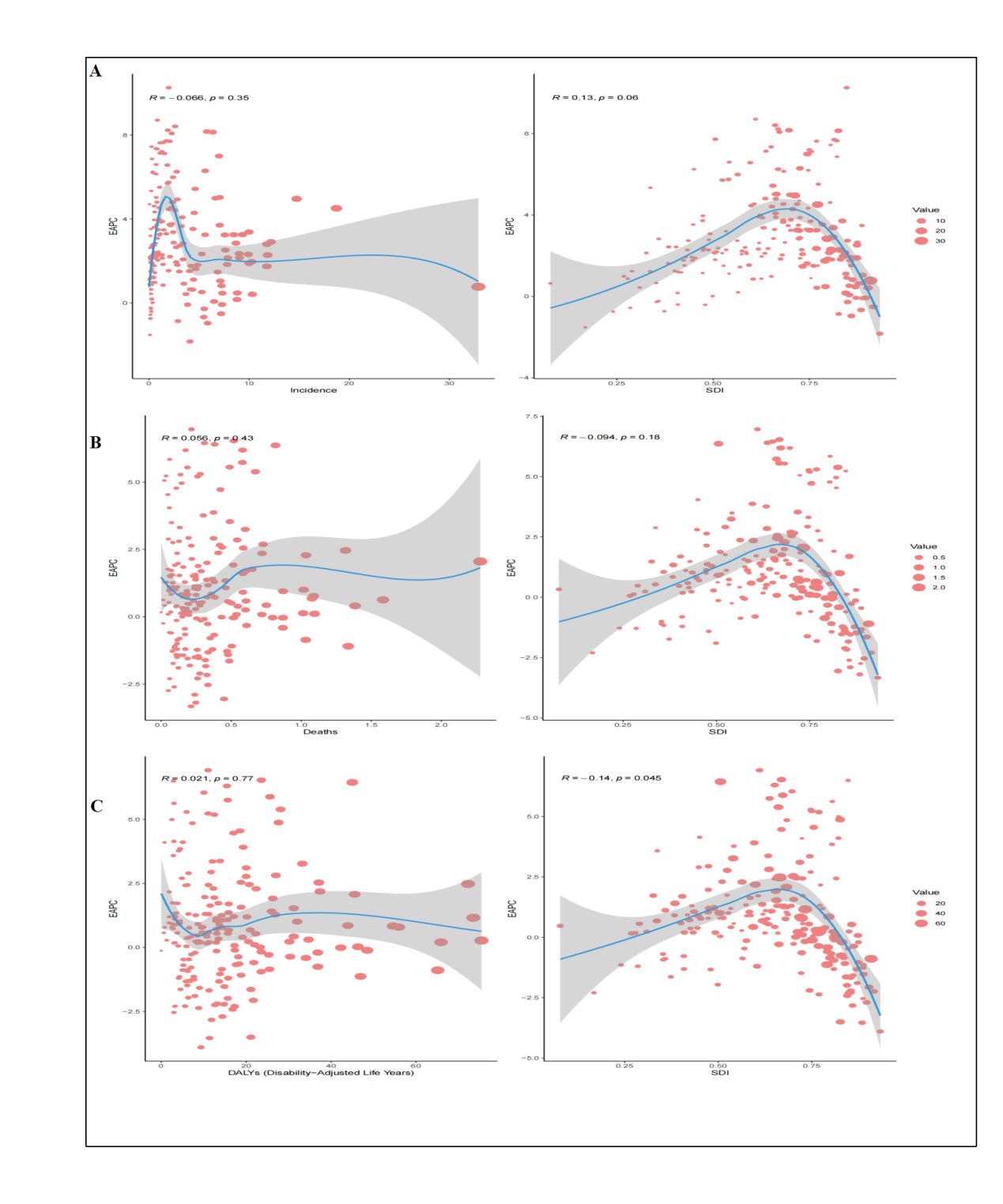


**Supplementary Figure S5:** Temporal trends in testicular cancer across different age groups globally, 1990–2021. A, Number of incidence cases. B, Incidence rate. C, Number of death cases. D, Mortality rate. E, Number of DALYs. F, DALYs rate.


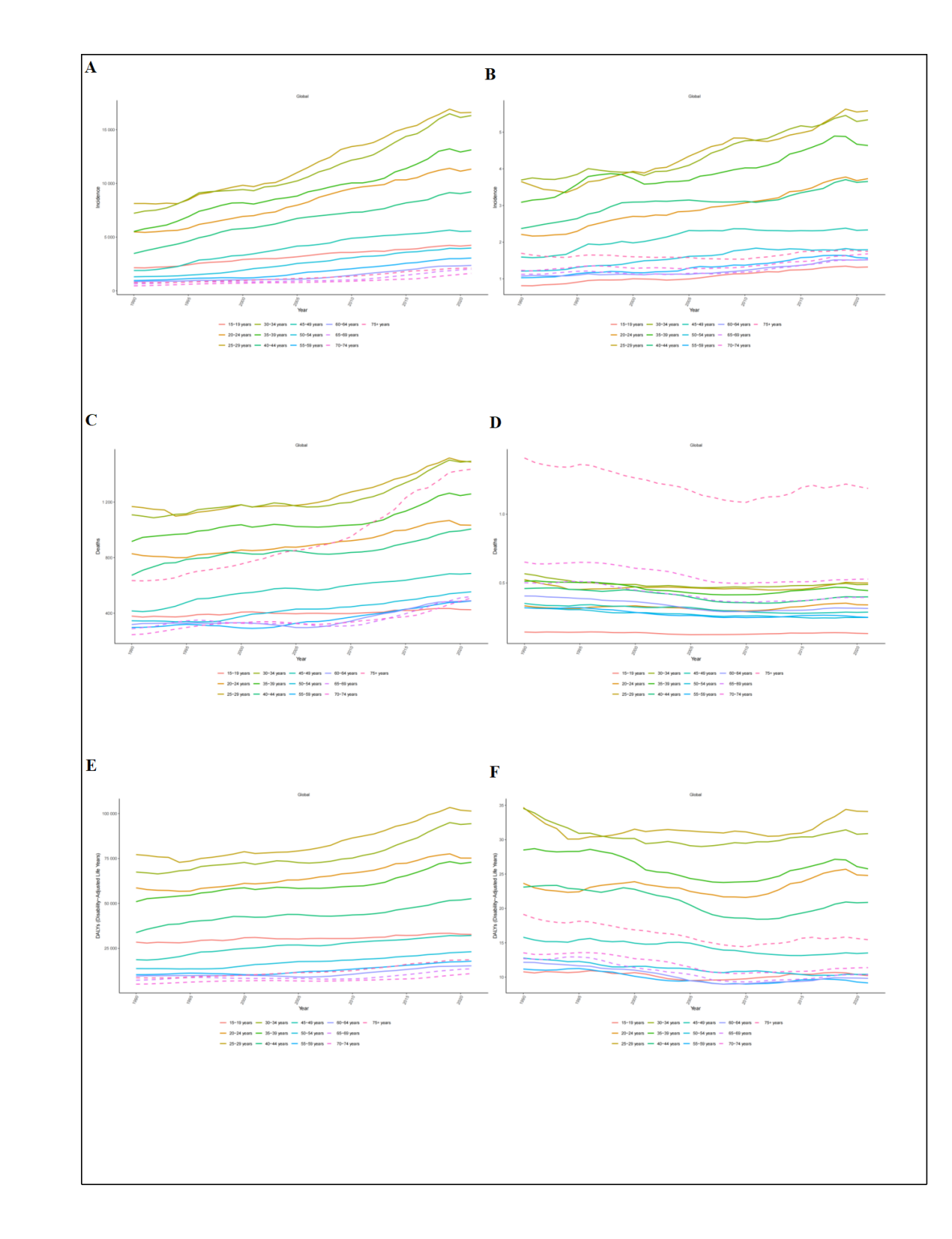

Supplement: Supplementary file 1 [file DataSheet1.docx]
